# Fisetin-loaded nanoparticles as a novel approach for cholesterol regulation in hypercholesterolemia: targeting the ASGR1-mediated mTORC1/AMPK pathway

**DOI:** 10.1186/s12951-026-04181-z

**Published:** 2026-02-27

**Authors:** Ziyang Zhang, Xi Chen, Xiaoshuang Hu, Junrui Liang, Huiru Huang, Wenxin Lu, Miaomiao Zhu, Mengxue Fang, Longfei Yin, Wen Li, Shenshen Zhang

**Affiliations:** 1 College of Public Health, Zhengzhou University, Zhongyuan District, Zhengzhou, 450007 China; 2 Food Laboratory of Zhongyuan, Yancheng District, Luohe, 462300 China

**Keywords:** Hypercholesterolemia, Fisetin, Cholesterol efflux, Asialoglycoprotein receptor 1, Carboxymethyl chitosan, Β-cyclodextrin, Fisetin nanoparticle

## Abstract

Hypercholesterolemia is a major risk factor for the development of atherosclerotic cardiovascular disease. The flavonoid fisetin shows potential cholesterol-regulating effects. Nevertheless, poor solubility and low bioavailability limit its clinical application. This study investigates fisetin’s effects on hypercholesterolemia and underlying mechanisms. Using a hypercholesterolemic mouse model, we observe that fisetin significantly attenuates hepatic steatosis, decreases total cholesterol (TC), triglycerides (TG), and low-density lipoprotein cholesterol (LDL-C) levels, and alleviates oxidative stress. Consistent results are observed in cellular experiments. Fisetin administration markedly modulates hepatic proteins involved in cholesterol excretion (ABCA1, ABCG5, ABCG8, CYP7A1, and LXRα) and cholesterol metabolism (HMGCR, LDLR, and SR-B1), thereby maintaining cholesterol homeostasis. Mechanistically, fisetin targets asialoglycoprotein receptor 1 (ASGR1), modulating the mTORC1/AMPK-BRCA1/BARD1 pathway. Notably, ASGR1 overexpression counteracts fisetin’s cholesterol-lowering effects. Additionally, carboxymethyl chitosan (CMCS)-modified β-cyclodextrin (β-CD)-based fisetin nanoparticles notably reduce lipid accumulation, TC and TG levels, even at concentrations one-fifth of free fisetin. In summary, fisetin effectively modulates cholesterol metabolism and represents a promising therapeutic approach for managing hypercholesterolemia. The fisetin nanoparticles offer an innovative strategy to overcome fisetin’s clinical limitations, enhancing its bioavailability and therapeutic efficacy.

**Graphical abstract:**

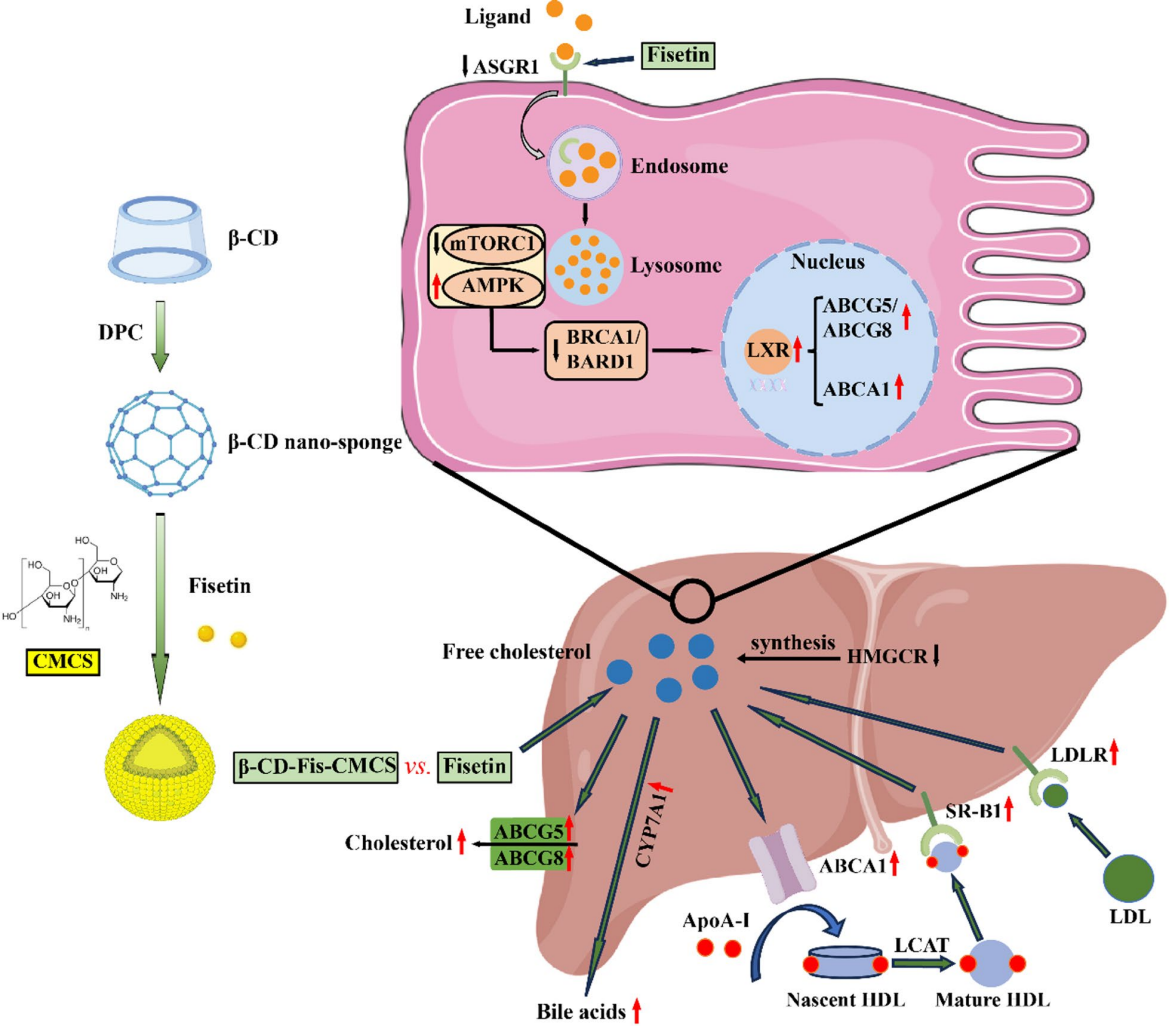

**Supplementary Information:**

The online version contains supplementary material available at 10.1186/s12951-026-04181-z.

## Introduction

Atherosclerotic cardiovascular disease (ASCVD), characterized by the formation of dense deposits within the arterial walls, is a leading cause of morbidity and mortality worldwide [1]. Extensive evidence has established that hypercholesterolemia, characterized by high concentrations of cholesterol-rich low-density lipoproteins (LDL) and their oxidatively damaged forms (oxLDL), is a major risk factor in ASCVD [2, 3]. Therefore, effective prevention and management of hypercholesterolemia are crucial for reducing the incidence and mortality of ASCVD.

Reverse cholesterol transport (RCT) is a central pathway for maintaining cholesterol homeostasis, and its dysregulation is pivotal to hypercholesterolemia [4]. Cholesterol excretion constitutes a critical step in RCT. ATP-binding cassette transporter A1 (ABCA1), a crucial receptor in the initial stages of RCT, mediates cholesterol efflux from hepatocytes and its loading into lipid-poor apolipoprotein A-I, thereby facilitating the formation of nascent high-density lipoprotein (HDL) particles. Cholesterol within these particles is esterified by lecithin cholesterol acyltransferase and is subsequently taken up by the liver [5]. In the liver, bile acid synthesis serves as a key mechanism for the removal of surplus cholesterol. Cholesterol 7α-hydroxylase (CYP7A1) converts cholesterol into bile acids, which are subsequently excreted into the bile [6]. Moreover, multiple transporters located on the bile canalicular membrane mediate cholesterol secretion into bile, thus facilitating cholesterol efflux, notably the heterodimeric ABCG5 and ABCG8 [7]. Therefore, the RCT pathway is vital for maintaining cholesterol homeostasis.

Asialoglycoprotein receptor 1 (ASGR1), predominantly expressed on hepatocytes, has emerged as a key regulator of cholesterol metabolism and LDL clearance [8]. Recent research has identified that ASGR1 variants with functional loss correlate with lower cholesterol levels and decreased cardiovascular disease risk [9]. Zhang et al. found that ASGR1 deficiency promotes cholesterol efflux and inhibits atherosclerosis in Western diet-fed ApoE^−/−^ mice [10]. Thus, ASGR1 holds potential as a regulator of hypercholesterolemia development through its role in promoting cholesterol efflux.

Currently, statins, which are the first-line agents for hypolipidemic therapy, primarily act by inhibiting hydroxy-methyl-glutaryl-coenzyme A reductase (HMGCR) to reduce LDL cholesterol (LDL-C) levels. The implementation of statins, however, is complicated by the risk of treatment-emergent adverse events, notably myopathic symptoms, heightened diabetes risk, and central nervous system adverse effects [11, 12]. Therefore, the search for novel therapeutic agents is essential for the management of hypercholesterolemia. Fisetin is a natural compound, abundant in fruits and vegetables, that possesses multiple biological activities [13]. Fisetin exhibits a favorable safety profile in mouse models, demonstrating efficacy at dosages between 20 and 200 mg/kg without inducing adverse effects [14, 15]. Evidence from multiple studies indicates that fisetin can lower cholesterol levels. For example, Li et al. demonstrated that fisetin improves atherosclerosis by regulating the expression of PCSK9 and LOX-1 [16]. However, it remains unclear whether fisetin modulates cholesterol metabolism *via* ASGR1 to inhibit the progression of hypercholesterolemia. Therefore, further investigation is warranted to elucidate how fisetin alleviates hypercholesterolemia and the underlying mechanisms involved.

Poor solubility, short half-life, and low bioavailability of fisetin severely limit its development and clinical application [13]. Nanodrug delivery systems, particularly nanoparticle encapsulation, have demonstrated notable success in addressing drug solubility and oral bioavailability [17]. Notably, sugars, especially cyclodextrin (CD) microparticles, are extensively utilized due to their facile preparation, tunable mechanical properties, excellent biocompatibility, and non-toxic nature. The unique structure of CD, characterized by a hydrophobic inner cavity and a hydrophilic outer surface rich in hydroxyl groups, makes it an ideal carrier for controlled drug release [18]. Carboxymethyl chitosan (CMCS), prized for its wide source and favorable molecular properties, facilitates liver targeting, cellular transfection, and modulation of efflux pumps. Additionally, CMCS is widely employed in drug delivery systems owing to its sustained-release capability, enhanced bioavailability, and minimal adverse effects [19, 20]. Previous studies have demonstrated that loading anthocyanin-3-glucoside onto β-CD-epichlorohydrin-CMCS complexes can significantly improve its stability against thermal and photodegradation [21]. Nevertheless, no study has investigated CMCS-modified CD to overcome the clinical limitations of fisetin. Consequently, additional investigations are warranted to explore the preparation method of fisetin nanoparticles and identify the optimal CD, as well as to elucidate its potential in addressing the challenges of fisetin.

Hence, this study aims to evaluate the cholesterol-lowering potential of fisetin against hypercholesterolemia and to uncover the underlying mechanisms. Additionally, we investigated whether encapsulating fisetin in CMCS modified β-CD could enhance its bioavailability and cholesterol-lowering efficacy. These findings provide strong evidence for fisetin’s potential as a therapeutic option against hypercholesterolemia.

## Methods and materials

### Reagents

Fisetin (≥ 98%) was sourced from Yuanye (Shanghai, China). 25-Hydroxycholesterol (≥ 98%) and Cholesterol (≥ 98%) were obtained from Sigma-Aldrich (St. Louis, MO, USA). Simvastatin and Filipin III were bought from Meilunbio (Dalian, China). Assay kits for total cholesterol (TC), malondialdehyde (MDA), triglyceride (TG), superoxide dismutase (SOD), LDL-C and HDL-C were purchased from Jiancheng Bio (Nanjing, China). Overexpression-ASGR1 (OE-ASGR1) plasmid was obtained from Sangon Biotech (Shanghai, China).

### Animal experiments

Male C57BL/6J mice, aged 8 weeks, were purchased from the SPF Biotechnology Co., Ltd. (Certificate: SCXK(Yu) 2023-0013). Mice were housed under standardized conditions featuring a 12 h light/dark cycle, a stable temperature of 20 ± 2℃, and a relative humidity of 50 ± 5%. Food and water were available without restriction. The study protocol was reviewed and approved by the Ethics Committee of Zhengzhou University (Protocol No. ZZUIRB2022-140) on September 15, 2022.

Sixty-four mice were randomly allocated into eight groups (*n* = 8 per group): Control group, Model group, high fat and high cholesterol (HFHC) diet supplemented with fisetin (Fis, 12.5 mg/kg), HFHC diet supplemented with fisetin (25 mg/kg), HFHC diet supplemented with Simvastatin (Sim, 5 mg/kg), HFHC diet supplemented with β-CD-CMCS, HFHC diet supplemented with β-CD-Fis-CMCS (5 mg/kg of fisetin), HFHC diet supplemented with β-CD-Fis-CMCS (10 mg/kg of fisetin). The dosage of fisetin adhered to our previous studies [22]. While the control group was fed a standard chow diet throughout the study, all other groups received an HFHC diet (Diet formulation, D12109C). All chows were obtained from HFK Bio (Beijing, China). After 4 weeks, each group was orally administered fisetin, β-CD-CMCS, β-CD-Fis-CMCS or simvastatin for 8 weeks. Following the intervention, blood samples were collected *via* cardiac puncture after a 12-h fast. The median lobe of the liver was fixed, and the remaining liver tissue was stored at -80℃.

### Oil red O staining

Following 24 h fixation in 4% paraformaldehyde and overnight dehydration, the samples were embedded in OCT medium in a cryostat and sectioned into 5 μm slices at -20℃. Following the manufacturer’s instructions, the Oil Red O working solution was prepared. The frozen sections were stained with Oil Red O working solution for 8–10 min, rinsed twice with 60% isopropanol, and then rinsed with distilled water, and counterstained with hematoxylin for 3–5 min. Finally, the sections were imaged using a microscope system. The positive area of Oil Red O staining was defined as the presence of deep crimson, granular lipid droplets.

### Lipid levels and oxidative stress analysis

The levels of LDL-C, HDL-C, TC, TG, MDA and SOD were measured using commercial assay kits.

### Cell culture

The AML12 cells, a murine liver cell line, sourced from the Chinese Academy of Sciences (CRL-2254), were cultured in DMEM/F12 medium (Gibco, NY, USA) supplemented with 10% fetal bovine serum (FBS) at 37℃ and 5% CO_2_. To establish the HC model, the cells were treated with specified concentrations of cholesterol and 25-hydroxycholesterol. Simultaneously, the cells were administered fisetin, β-CD-Fis-CMCS, β-CD-CMCS or simvastatin at the indicated concentrations.

### Cell transfection

AML12 cells were transfected with either the pcDNA3.1(+) plasmid or negative control constructs using I’maFect Ⅱ (Yimeiang, Beijing, China) according to the manufacturer’s protocol, followed by 24-h incubation.

### Cell counting kit-8 (CCK-8) assay

The AML12 cells were incubated with fisetin at 0–5 µg/mL concentrations. Following a 24-h treatment, cells were incubated with CCK-8 working solution for 30 min at 37 °C without light, and the absorbance at 450 nm was then measured by a microplate reader (Molecular Devices, CA, USA).

### Filipin Ⅲ staining

After 48-h treatment with fisetin and simvastatin, the AML12 cells were fixed with 4% paraformaldehyde for 1 h, stained with Filipin Ⅲ working solution for 2 h, and then examined under a fluorescence microscope (Nikon, Tokyo, Japan).

### Molecular Docking

The molecular structure of fisetin and the protein structure of ASGR1 (ID: P34927) were sourced from the PubChem website and UniProt database, respectively. Molecular docking simulations were conducted using AutoDockTools, and the resulting binding affinities were calculated to assess binding energy. Results were visualized using PyMOL software, following the methodology of Ashraf et al. [23].

### Cellular thermal shift assay (CETSA)

The binding of fisetin to ASGR1 was verified by CETSA. After lysis for 30 min, the cells lysates were centrifuged at 4℃, 12,000 g for 15 min, and the supernatant was collected. Subsequently, the supernatant was incubated with either fisetin (2 µg/mL) or control (absolute ethanol) for 1 h. Fisetin and control group were equally then aliquoted into eight EP tubes and subjected to heating at different temperatures (37℃, 42℃, 47℃, 52℃, 57℃, 62℃, 67℃ and 72℃) for 5 min, followed by cooling at room temperature for 3 min. Subsequently, each sample was centrifuged at 4℃, 20,000 g for 20 min and collected the supernatant. Western blotting was performed to detect ASGR1 expression.

### Immunofluorescence

AML12 cells were treated with indicated drugs and then fixed with ice-cold methanol for 10 min. Following permeabilization with 0.1% Triton X-100 for 10 min, the cells were blocked with 5% BSA for 1 h. Next, the cells were incubated with primary antibodies against ASGR1 (Proteintech, Wuhan, China, 11739-1-AP, 1:200) and Cluster of Differentiation 63 (CD63) (Abmart, Shanghai, China, M051014, 1:200) for 2 h. Subsequently, the cells were incubated with FITC-conjugated Goat Anti-Mouse IgG (Abbkine, Wuhan, China, A22110, 1:500) and TRITC-conjugated Goat Anti-Rabbit IgG (Zenbio, Chengdu, China, 511202, 1:500) for 1.5 h. The nuclei were then stained with 1 µg/mL DAPI for 10 min. Finally, fluorescent images were acquired using a fluorescence microscope (Nikon, Tokyo, Japan).

### Real-time quantitative PCR analysis

Total RNA was extracted from liver tissue and AML12 cells using an RNA extraction reagent (Vazyme, Nanjing, China). Complementary DNA was synthesized from the RNA with one-step RT-gDNA digestion SuperMix (Yeasen, Shanghai, China). Quantitative PCR was performed using TransScript Green two-step qRT-PCR Supermix (Yeasen, Shanghai, China) on a real-time PCR system. The relative gene expression levels were calculated using the 2^−ΔΔCt^ method. The primer sequences are listed in Table 1.

**Table 1:** Primer sequences for quantitative PCR

Gene	Forward	Reverse
*m-Abca1*	CGTTTCCGGGAAGTGTCCTA	GCTAGAGATGACAAGGAGGATGGA
*m-Abcg5*	AGGGCCTCACATCAACAGAG	GCTGACGCTGTAGGACACAT
*m-Abcg8*	ATACCCTGGAGGTCTCATAGCA	ACGTCGAGTAGTGAGGCTCTC
*m-Lxrα*	AGGGATAGGGTTGGAGTCAGC	CGTTGTAATGGAAGCCAGAGG
*m-Asgr1*	CTCACCCACAAAAAGGTCCG	GAATGCTGAGGCTCGAGGAG
*m-Cyp7a1*	GGGATTGCTGTGGTAGTGAGC	GGTATGGAATCAACCCGTTGTC
*m-Hmgcr*	AGCTTGCCCGAATTGTATGTG	TCTGTTGTGAACCATGTGACTTC
*m-Ldlr*	TTCAGTCCCAGGCAGCGTAT	TTGATCTTGGCGGGTGTT
*m-Srb1*	TGTACTGCCTAACATCTTGGTCC	ACTGTGCGGTTCATAAAAGCA
*m-Gapdh*	ACGGCAAATTCAACGGCACAG	AGACTCCACGACATACTCAGCAC

### Western blot analysis

Total protein was extracted from samples using RIPA lysis buffer. Subsequently, Western blot assessed corresponding protein expression. Antibodies are involved in this paper as follows: anti-HMGCR antibody (HuaBio, Hangzhou, China, ET1702-41, 1:5000), anti-LDLR antibody (Santa Cruz, Dallas, USA, sc-18823, 1:1000), anti-CYP7A1 antibody (ABclonal, Wuhan, China, A10615, 1:1000), anti-SR-B1 antibody (Proteintech, 21277-1-AP, 1:1000), anti-ABCA1 antibody (Proteintech, 26564-1-AP, 1:1000), anti-ABCG5 antibody (BOSTER, Wuhan, China, A01503-2, 1:1000), anti-ABCG8 antibody (HuaBio, ER1903-17, 1:1000), anti-LXRα antibody (Proteintech, 14351-1-AP, 1:4000), anti-ASGR1 antibody (Proteintech, 11739-1-AP, 1:10,000), anti-p70 S6 Kinase antibody (Abways, Shanghai, China, CY5365, 1:2000), anti-phospho-p70 S6 kinase (T389) antibody (HuaBio, HA721803, 1:2000), anti-AMPKα antibody (Abmart, TA6423, 1:1000), anti-phospho-AMPKα (Thr172) antibody (Abmart, T55608, 1:1000), anti-phospho-mTOR antibody (Abmart, T55996, 1:1000), anti-mTOR antibody (Abmart, T55306, 1:1000), anti-BRCA1 antibody (Immunoway, Plano, USA, YT0517, 1:1000) and anti-BARD1 antibody (Santa Cruz, sc-74559, 1:500).

### Preparation of 2-HP-β-CD/β-CD-Fis-CMCS and FITC@β-CD-CMCS

The 2-HP-β-CD/β-CD nano-sponge was synthesized via a solvent-free melt method. Briefly, 2-HP-β-CD/β-CD and diphenyl carbonate (DPC) were mixed at molar ratios of 1:2, 1:4 and 1:6. The mixture was placed in a beaker on a heating magnetic stirrer and stirred at 90℃ for 5 h. The resulting product was ground and washed repeatedly with ultrapure water and acetone. It was then extracted with anhydrous ethanol for 24 h. The phenol byproducts were removed by rotary evaporator, and the final product was dried at 60℃ for 72 h. For fluorescence labeling, 1 mg of FITC was dissolved in 10 mL of ultrapure water containing 10 mg of β-CD-CMCS, and the solution was stirred for 10 h. The resulting orange FITC@β-CD-CMCS was washed twice with methanol and water. The 2-HP-β-CD/β-CD nano-sponge and fisetin were weighed and dissolved in a CMCS solution containing 20% (v/v) ethanol. The mixture was ultrasonicated for 10 min and then stirred without light for 24 h. The next day, this mixture was centrifuged at 2000 r/min for 10 min. The supernatant was collected and lyophilized to obtain the final product.

### Characterization of Fisetin and β-CD-Fis-CMCS

The maximum absorption wavelength of fisetin was determined by performing a full-wavelength ultraviolet scan of a standard fisetin solution from 230 to 500 nm. To establish the standard curve, a series of fisetin standard solution (0, 0.05, 0.1, 0.2, 0.5, 1.0, 2.0 mg/mL) were precisely prepared and their concentrations were analyzed using high-performance liquid chromatography (HPLC). The size and zeta potential of β-CD-Fis-CMCS were measured using dynamic light scattering at 25℃. The morphologies of nano-sponge and β-CD-Fis-CMCS were observed employing scanning electron microscope (SEM) and transmission electron microscope (TEM). For SEM sample preparation, the freeze-dried samples were evenly spread on the conductive silicon substrate and sputter-coated with a thin layer of gold using a sputter coater. Subsequently, the samples were observed under an SEM at an accelerating voltage of 3.0 kV. For TEM sample preparation, the diluted β-CD-Fis-CMCS suspension was first ultrasonicated for 5 min. An 8 µL aliquot was deposited onto the copper grid. Subsequently, negative staining was performed with phosphotungstic acid solution for 2 min and then the grid was dried with an infrared lamp for 20 min.

### Determination of encapsulation efficiency and drug loading

Firstly, β-CD-Fis-CMCS was weighed and dissolved in a methanol, and then the solution was sonicated for 10 min to dissociate the nano-sponge complex and release the encapsulated fisetin. Finally, the resulting solution was appropriately diluted and analyzed at 450 nm using HPLC. The encapsulation efficiency and drug loading were calculated using the following formulas.

$$\begin{aligned}\mathrm{EE}\%&\left(\text{encapsulation efficiency}\right) =\frac{\text{Total number of Fis loaded}}{\text{Total number of Fis added}}\times 100\%\end{aligned}$$

$$\begin{aligned}\mathrm{DL}\%\:\left(\text{drug loading}\right)&=\frac{\text{Total number of Fis loaded}}{\text{Total number of Fisetin nanoparticles}}\times 100\%\end{aligned}$$

### Kinetic modelling for the in vitro release of 2-HP-β-CD/β-CD-Fis-CMCS

Phosphate-Buffered Saline (PBS, PH 7.4) was prepared to simulate colonic fluid. 20 mg of 2-HP-β-CD/β-CD-Fis-CMCS were dissolved to 10 mL of PBS, oscillated in water bath shaker at 37℃ and 100 rpm. Each experimental group was run in triplicate. Samples were withdrawn at predetermined time intervals (2, 4, 6, 8, 10, 12, 24, 36, 48 and 72 h), and centrifuged at 4℃ and 14,000 rpm for 30 min. Each time a sample was withdrawn, an equal volume of fresh PBS was added to maintain a constant total volume. Then, the 100 µL supernatant was measured using HPLC at 364 nm to calculate the cumulative release of fisetin. Different kinetic models (namely the zero-order model, first-order model, Higuchi model, Weibull model Hixson-Crowell model and Ritger-peppas model, respectively) were adapted to ascertain the fisetin rate-controlling processes and release mechanism in vitro (Table 2).

**Table 2:** Dynamic model and formulation

Model	Formula
Zero-order model	Y = kt + C
First-order model	ln(1-Y) = kt + C
Higuchi model	Y = kt^1/2^ + C
Weibull model	Y = A(1-e^− m(t−τ)^t0^)
Hixson-Crowell model	(1-Y)^1/3^ = -kt + C
Ritger-peppas model	Y = kt^n^

Y is the amount of fisetin released at time; t, k is the release rate constant, C is the constant; A is the fixed value 100; t0 is the scale parameter, τ is the position parameter, representing the lag time before fisetin dissolution or release; m is the shape parameter that determines the shape of the fitted curve; n is a characteristic parameter that characterizes the release mechanism.

### Statistical analysis

SPSS 25.0 was used to display the statistical analysis and tested one-way analysis of variance. Data were exhibited as the mean ± SD, and all experiments were repeated three times unless otherwise noted. When *p* < 0.05, differences were deemed statistically significant.

## Results

### Fisetin alleviated liver steatosis in mice

**Fig. 1:**
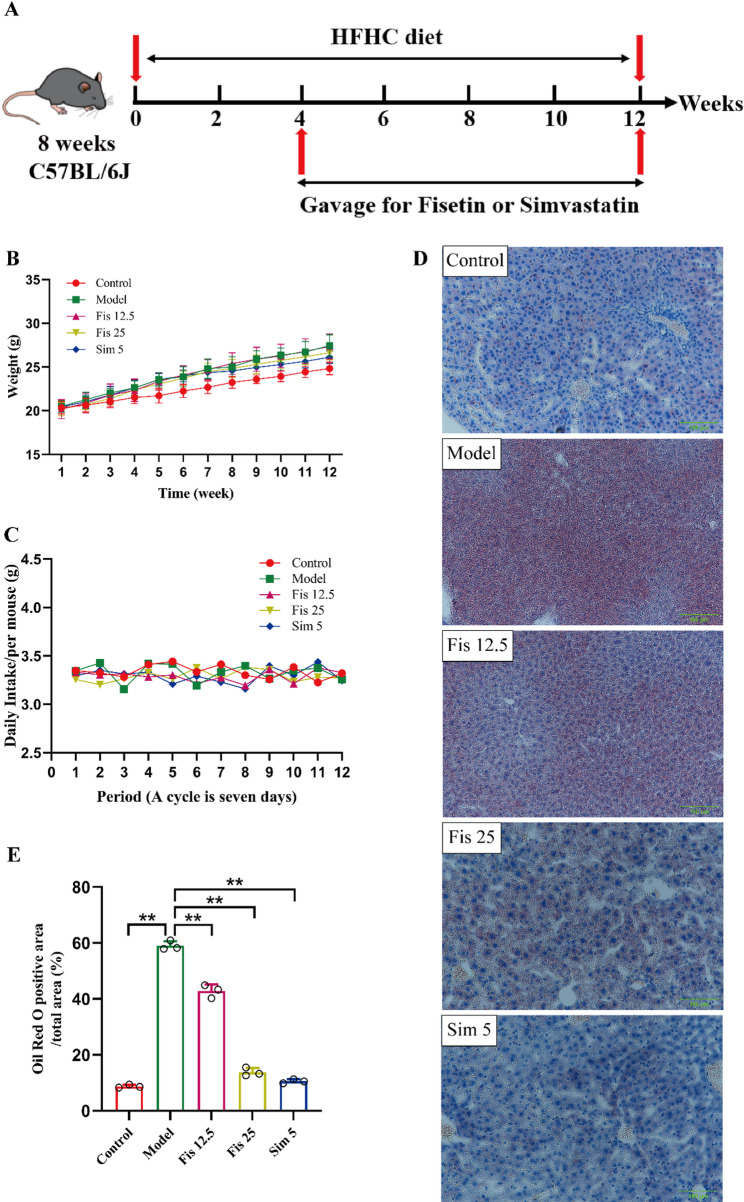
Fisetin ameliorated hepatic steatosis in HFHC-fed C57BL/6J mice. (**A**) Flow chart of mice experiments. Measurements and calculations were made for relative body weight and food intake. (**B**) Body weight. (**C**) Daily food intake per mouse throughout the intervention period. (**D**) Representative images of ORO staining in the liver (magnification, 200 ×) (**E**) Quantitative analysis of ORO-positive area in mice liver. Data are mean ± SD, *n* = 8 or 3. * *p* < 0.05, ** *p* < 0.01

HFHC-fed C57BL/6J mice were employed to model hypercholesterolemia (Fig. 1A). Body weight increased steadily throughout the experiment. Relative to the controls, mice in the experimental groups gained significantly more weight (All *p*-values < 0.05) (Fig. 1B). The amount of food consumed by each mouse remained stable, showing only slight variations (Fig. 1C). After 12 weeks of HFHC treatment, ORO staining revealed significant lipid accumulation in the model group (*p* < 0.0001). Fisetin treatment notably reduced hepatic lipid accumulation by approximately 28% (12.5 mg/kg, *p* < 0.0001) and 77% (25 mg/kg, *p* < 0.0001) compared with HFHC-fed mice (Fig. 1D, E).

### Fisetin alleviated dyslipidemia and hepatic oxidative stress in C57BL/6J mice

**Fig. 2:**
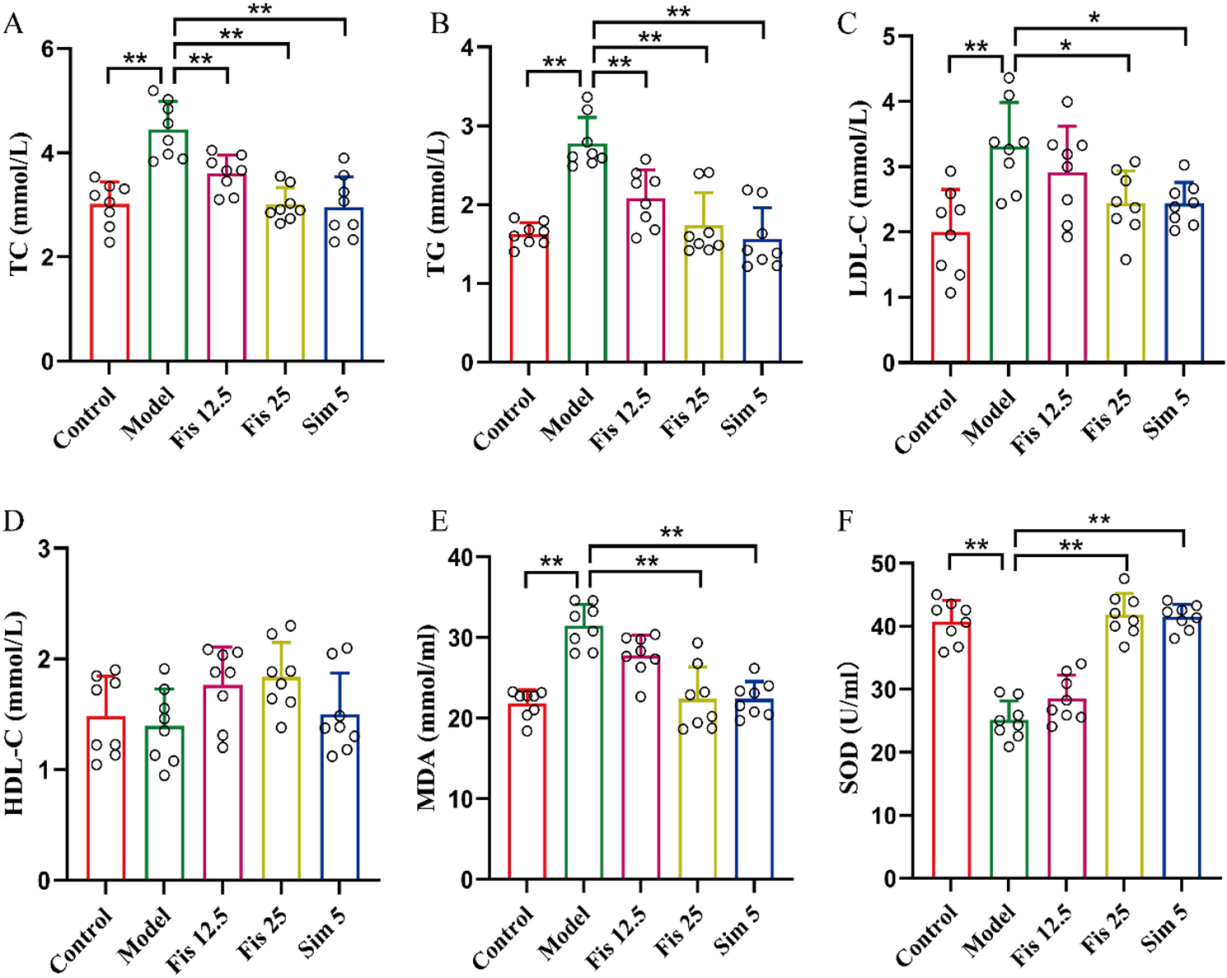
Fisetin regulated lipid metabolism and oxidative stress in mice. (**A**) Levels of serum TC. (**B**) Levels of serum TG. (**C**) Levels of serum LDL-C. (**D**) Levels of serum HDL-C. (**E**) Serum MDA levels. (**F**) Serum SOD activity. Data are mean ± SD, *n* = 8. * *p* < 0.05, ** *p* < 0.01

Mice fed the HFHC diet for 12 weeks exhibited significantly higher serum levels of TC, TG, and LDL-C than the controls (all *p* < 0.001). Fisetin administration significantly reversed these changes (Fig. 2A-C). However, no significant differences in serum HDL-C were found between groups (All *p*-values > 0.10) (Fig. 2D). Oxidative stress is also prominent features of hypercholesterolemia. In the model group, MDA content was increased by 44%, while SOD activity was decreased by 38% relative to controls (*p* < 0.0001). Intervention of fisetin markedly reversed the trends (Fig. 2E, F). These findings suggest that fisetin could lower lipid levels and alleviate oxidative stress, potentially helping to prevent hypercholesterolemia.

### Fisetin modulated hepatic cholesterol metabolism through RCT pathway

**Fig. 3:**
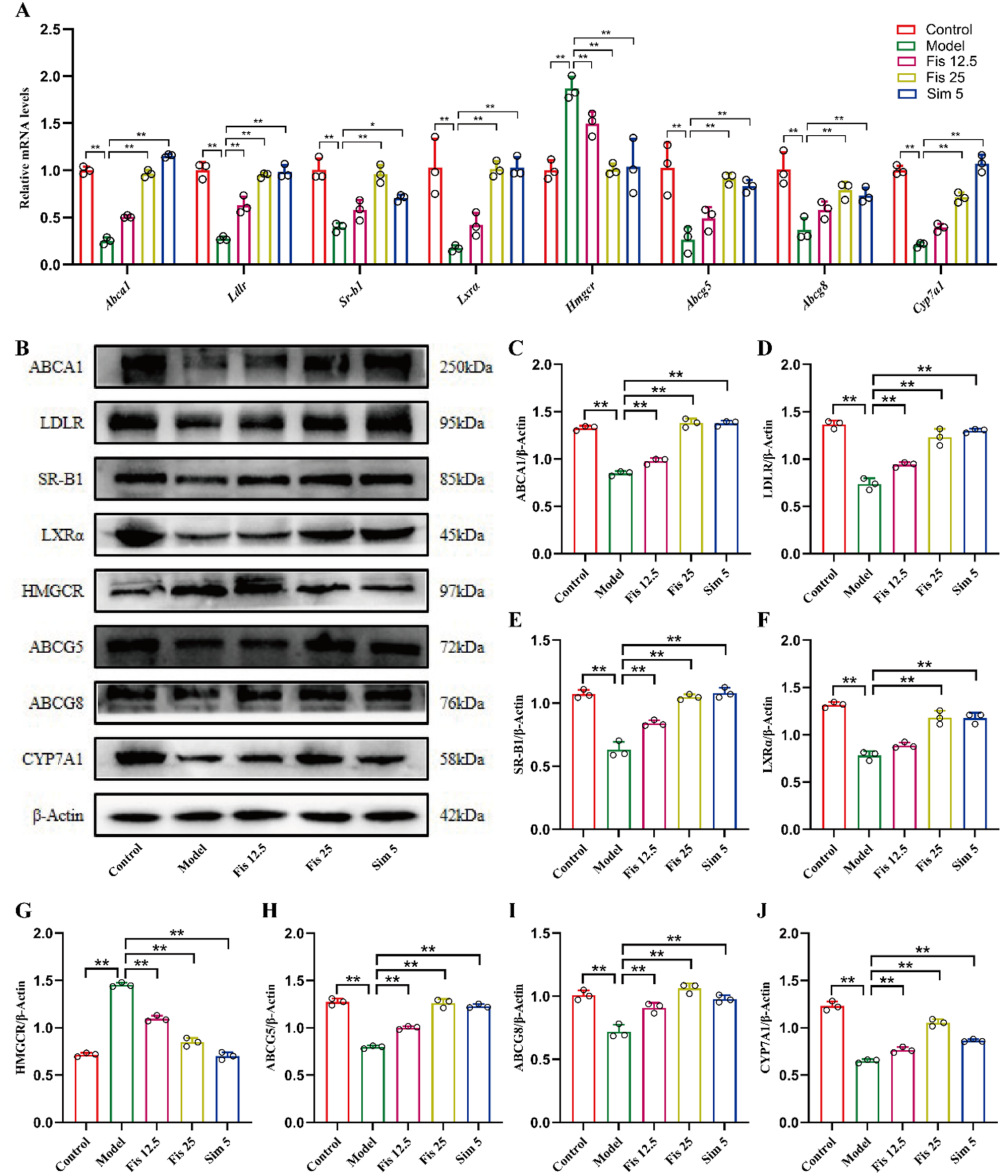
Fisetin regulated liver cholesterol metabolism in mice fed HFHC diet. (**A**) Quantification of hepatic mRNA levels for key cholesterol metabolism genes. (**B-J**) Western blot analysis of corresponding proteins in liver tissues. Data are mean ± SD, *n* = 3. * *p* < 0.05, ** *p* < 0.01

The RCT pathway represents a critical mechanism through which the liver maintains systemic cholesterol homeostasis. Following fisetin administration, key regulators of the RCT pathway, such as *Abca1*, *Ldlr* and *Sr-b1*, were upregulated by 282%, 248% and 142%, respectively (25 mg/kg, all *p* < 0.0001) relative to the experimental group (Fig. 3A). Consistently, fisetin treatment rescued the HFHC-induced inhibition of ABCA1, LDLR and SR-B1 protein expression (Fig. 3B-E). In addition, several key molecules involved in cholesterol metabolism, including *Lxrα*, *Hmgcr*, *Abcg5*, *Abcg8* and *Cyp7a1*, exhibited altered expression in the model group. Relative to controls, *Lxrα*, *Abcg5*, *Abcg8* and *Cyp7a1* were downregulated (*p* < 0.001), while *Hmgcr* was upregulated (*p* < 0.001). Administration of fisetin significantly reversed these changes (Fig. 3A). Moreover, in line with the findings on gene expression, fisetin not only restored the HFHC-mediated suppression of LXRα, ABCG5, ABCG8 and CYP7A1 protein levels but also inhibited the concomitant upregulation of HMGCR (All the *p*-values are lower than 0.0001) (Fig. 3B, F-J). These results indicate that fisetin may effectively activate the RCT pathway and stimulate cholesterol efflux.

### Fisetin modulated HC-induced AML12 cells cholesterol metabolism disorder

**Fig. 4:**
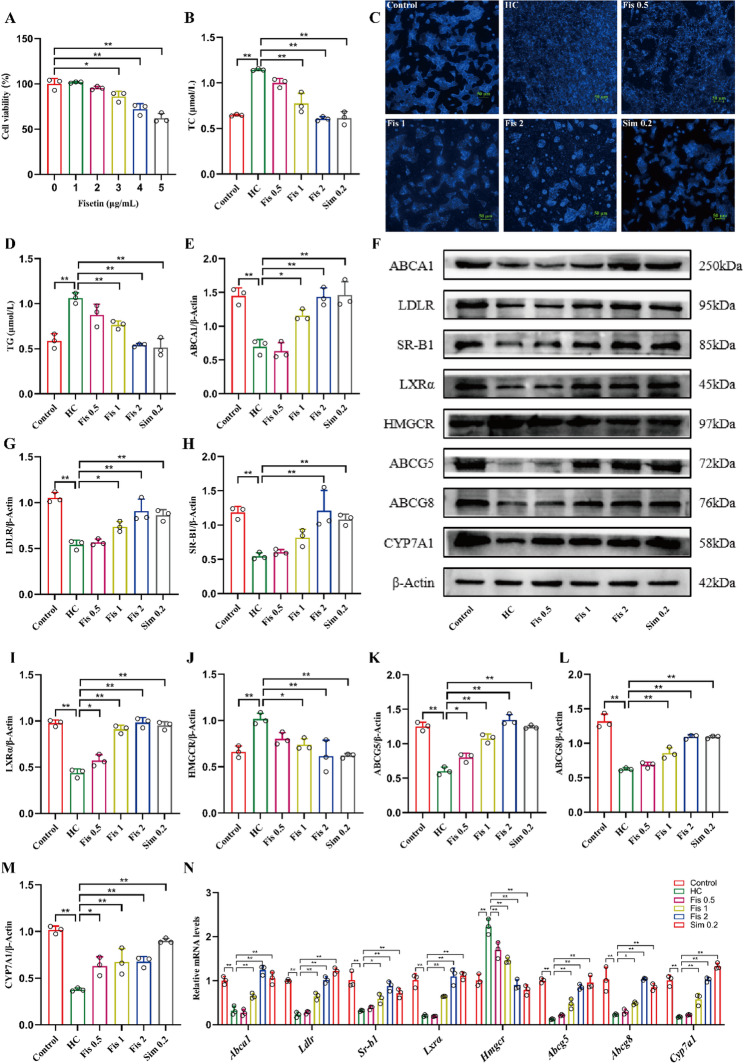
Effect of fisetin on cholesterol metabolism in HC-induced AML12 cells. (**A**) The cell viability of AML12 cells. (**B**) TC levels. (**C**) Filipin Ⅲ staining. Scale bar (50 μm), magnification (200 ×). (**D**) TG levels. (**E-M**) Western blot analysis of key regulators of cholesterol homeostasis in AML12 cells. (**N**) Quantification of mRNA levels for key cholesterol metabolism genes. Data are mean ± SD, *n* = 3. * *p* < 0.05, ** *p* < 0.01

The cholesterol-lowering effect of fisetin was assessed in AML12 cells in vitro. The cells were treated with fisetin (0–5 µg/mL) for 24 h. The findings revealed that fisetin was non-cytotoxic in AML12 cells at doses under 2 µg/mL (Fig. 4A). Therefore, fisetin at 0.5, 1, and 2 µg/mL was used in subsequent experiments. Subsequently, AML12 cells were co-treated with cholesterol (20 µg/mL) + 25-hydroxycholesterol (2 µg/mL) and varying concentrations of fisetin for 48 h, followed by measurement of TC and TG levels. The model group exhibited a marked elevation in TC and TG levels relative to controls (*p* < 0.0001). Conversely, treatment with fisetin successfully reduced the elevation of both TC and TG (Fig. 4B, D). Moreover, Filipin III staining showed severe cholesterol accumulation in the HC-induced AML12 cells, whereas fisetin administration alleviated this pathological condition (Fig. 4C). In addition, the protein expression levels of ABCA1, LDLR, SR-B1, LXRα, ABCG5, ABCG8 and CYP7A1 were significantly increased, whereas HMGCR levels were remarkably decreased with fisetin treatment (*p* < 0.01), relative to the HC group (Fig. 4E-M). Consistently, the similar results were observed across gene expression levels (Fig. 4N). Altogether, these findings indicate that fisetin alleviates HC-induced cholesterol metabolism disorder in vitro.

### ASGR1 as a molecular target for Fisetin in ameliorating hypercholesterolemia

**Fig. 5:**
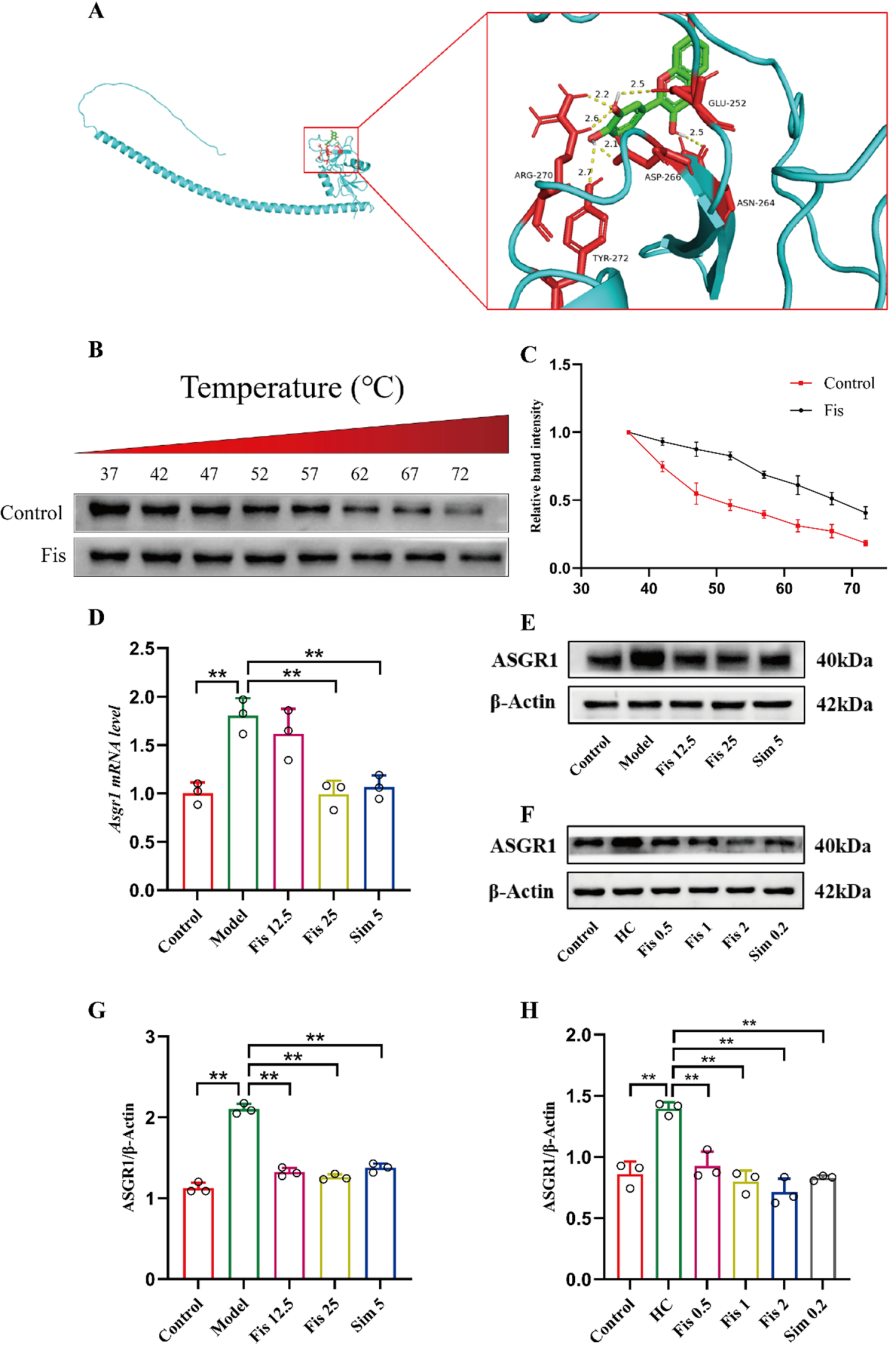
ASGR1 as the molecular target underlying the hypocholesterolemic mechanism of fisetin. (**A**) Molecular docking of fisetin with ASGR1. (**B-C**) CETSA. (**D**) Hepatic ASGR1 mRNA levels in mice. (**E-H**) Representative bands and quantitative statistical plots of ASGR1 protein expression in mice liver tissue and AML12 cells. Data are mean ± SD, *n* = 3. * *p* < 0.05, ** *p* < 0.01

Research has recently confirmed that ASGR1 is involved in regulating hepatic cholesterol metabolism and promoting cholesterol efflux. Therefore, additional experiments were conducted to investigate whether fisetin alleviates hypercholesterolemia by regulating ASGR1 expression and promoting cholesterol efflux. Molecular docking analysis revealed a low-affinity binding of fisetin to ASGR1. The predicted binding pose involved interactions with residues ARG-270, TYR-272, ASP-266, ASN-264 and GLU-252 (Fig. 5A). The binding energy of fisetin to ASGR1 was − 7.1 ± 0.4 kcal/mol (Table 3). Moreover, the CETSA results demonstrated that administration of fisetin increased protein expression of ASGR1 under the same temperature condition relative to controls (Fig. 5B, C). These data suggest that fisetin directly bind to ASGR1, thus confirming ASGR1 as a fisetin target. Meanwhile, fisetin treatment evidently reversed the model group upregulation of ASGR1, normalizing its mRNA and protein levels in liver tissue and AML12 cells (*p* < 0.0001) (Fig. 5D-H). These findings suggest that fisetin may regulate cholesterol efflux by targeting ASGR1.

**Table 3:** Molecular Docking results of Fisetin to ASGR1

Macromolecule	Binding energy	pKi	Number of hydrogen bonds	RMSD
ASGR1	-7.00 ± 0.17 kcal/mol	5.13 ± 0.13	6	0.001

RMSD, Root Mean Square Deviation

### Fisetin modulated cholesterol excretion through ASGR1-mTORC1/AMPK-BRCA1/BARD1 pathway

**Fig. 6:**
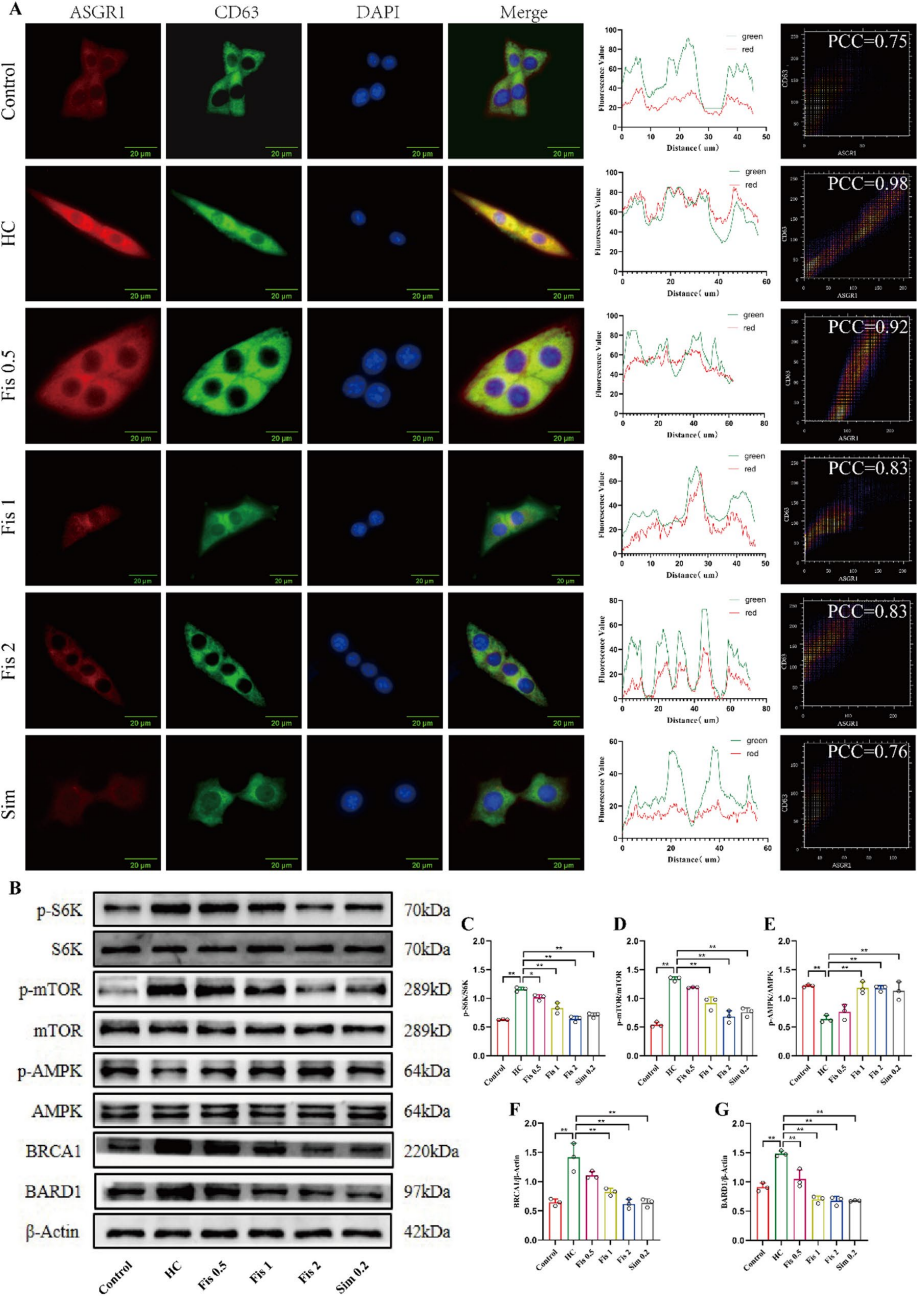
Fisetin reduced the co-localization between ASGR1 and lysosomes and regulated mTORC1/AMPK-BRCA1/BARD1 pathway. (**A**) Representative immunofluorescence images and statistical results of ASGR1 and CD63 protein expression in AML12 cells. Scale bar (20 μm), magnification (400 ×). (**B-G**) Western blot analysis of p-mTOR, p-S6K, p-AMPK, BRCA1 and BARD1 in AML12 cells. Data are mean ± SD, *n* = 3. * *p* < 0.05, ** *p* < 0.01

**Fig. 7:**
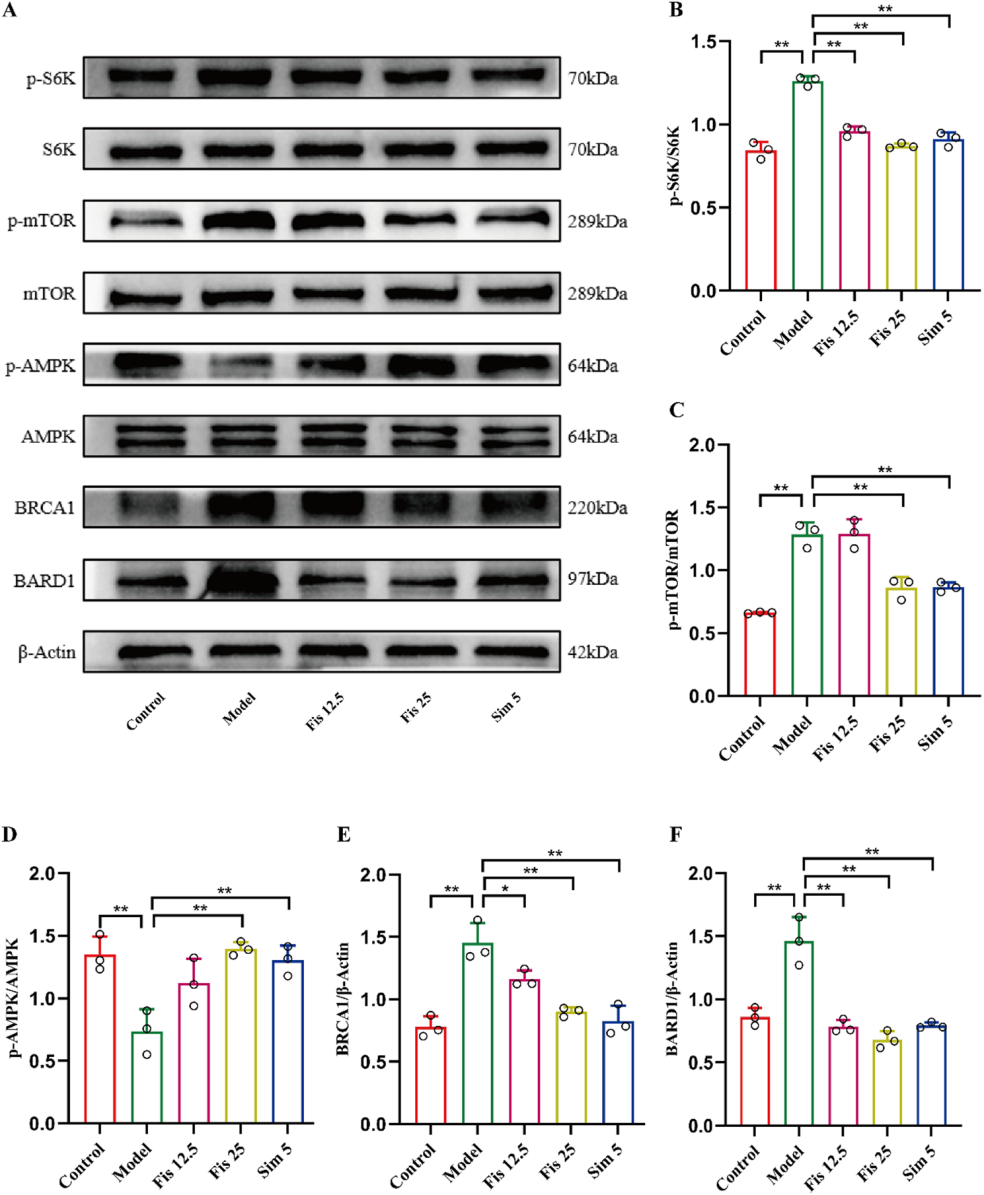
Fisetin regulated mTORC1/AMPK-BRCA1/BARD1 pathway in mice. (**A-F**) Western blot analysis of p-mTOR, p-S6K, p-AMPK, BRCA1 and BARD1 in mice. Data are mean ± SD, *n* = 3. * *p* < 0.05, ** *p* < 0.01

ASGR1 mediates the endocytosis of multiple asialoglycoproteins and delivers them to lysosomes for subsequent degradation [24]. In this study, the co-localization between ASGR1 and lysosomes was examined in fisetin-treated AML12 cells. The overlap of ASGR1 (red) and CD63 (green) in the HC-induced AML12 cells (Pearson correlation coefficient (PCC) = 0.98) was remarkably increased versus controls (PCC = 0.75). Fisetin administration remarkably reduced the overlap of fluorescence (0.5 µg/mL, PCC = 0.92, 1 µg/mL, PCC = 0.83, 2 µg/mL, PCC = 0.83, respectively). Moreover, fisetin treatment reversed the HC-induced enhancement of red fluorescence in AML12 (Fig. 6A). These findings suggest that fisetin can reduce ASGR1 activity, thereby regulating asialoglycoprotein degradation. Nutrients liberated by lysosomal degradation can activate mTORC1 and inhibit AMPK [25, 26]. As expected, HC-stimulated AML12 cells exhibited elevated phosphorylation levels of mTOR and S6K relative to controls (*p* < 0.0001). In contrast, AMPK phosphorylation was decreased (*p* = 0.0001) under the same conditions. However, fisetin treatment in AML12 cells resulted in significant reversal of these changes, with phosphorylation levels of mTOR, S6K and AMPK showing marked improvements (*p* < 0.01) (Fig. 6B-E). Additionally, fisetin treatment downregulated the HC-induced upregulation of BRCA1 and BARD1 protein expression (Fig. 6B, F-G). Similar results were observed in the mouse model. The HFHC diet significantly upregulated the phosphorylation levels of mTOR and S6K and the protein levels of BRCA1 and BARD1 in mouse liver relative to controls (all *p* < 0.05). Conversely, AMPK phosphorylation was significantly downregulated (*p* < 0.0001) in mouse liver (Fig. 7A-F). These findings illustrate that ASGR1 is crucial to fisetin’s ability to enhance cholesterol excretion, potentially through the mTORC1/AMPK-BRCA1/BARD1 pathway.

### Overexpression of ASGR1 abrogated Fisetin against HC-induced cholesterol efflux in AML12 cells

**Fig. 8:**
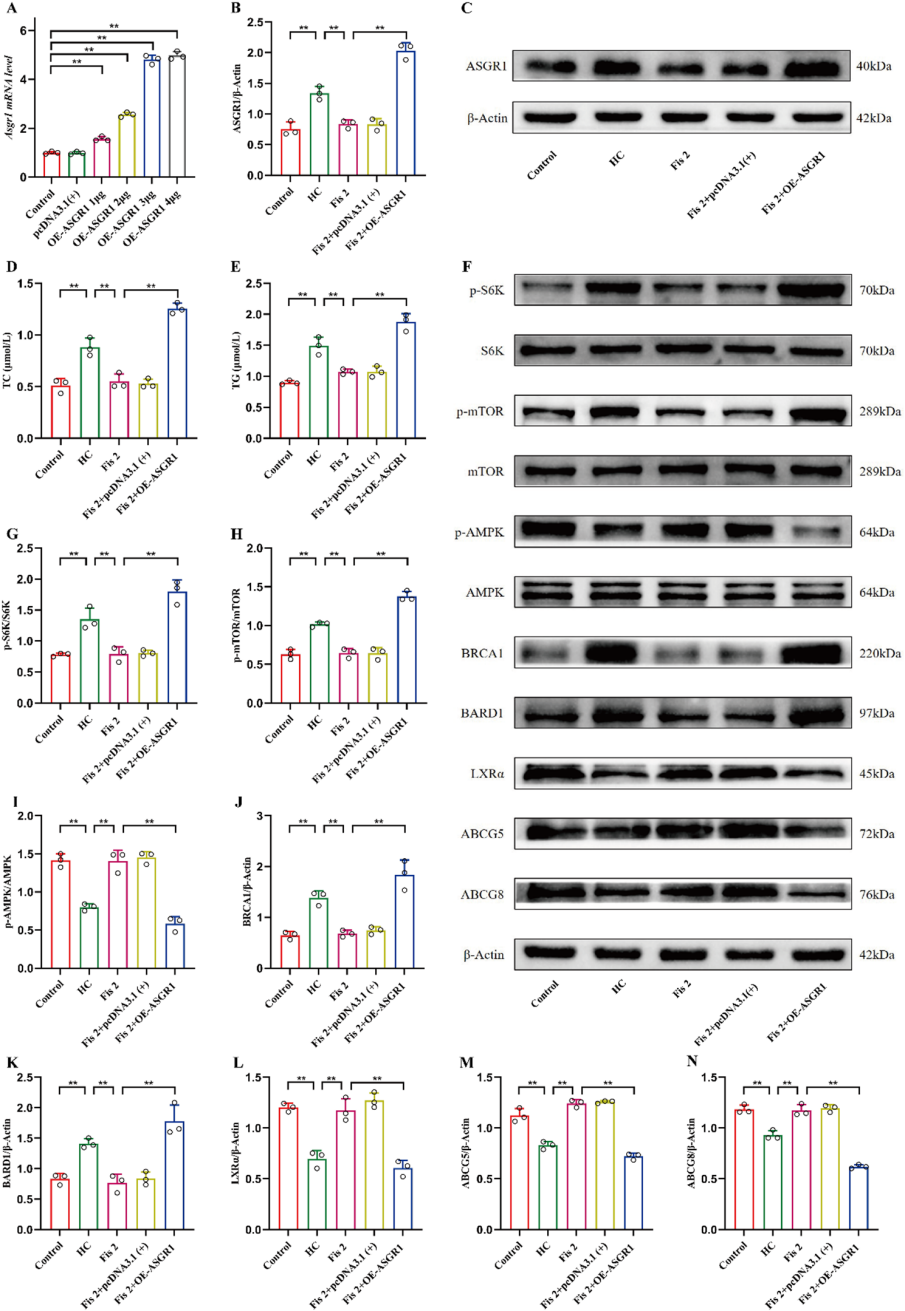
Overexpression of ASGR1 abrogated the protective effects of fisetin on AML12 cells. (**A**) mRNA expression levels of ASGR1. (**B-C**) Western blot analysis of ASGR1. (**D**) Levels of serum TC. (**E**) Levels of serum TG. (**F-N**) Western blot analysis of p-S6K, p-mTOR, p-AMPK, BRCA1, BARD1, LXRα, ABCG5 and ABCG8 in AML12 cells. Data are mean ± SD, *n* = 3. * *p* < 0.05, ** *p* < 0.01

The OE-ASGR1 plasmid was used to investigate the relationship between mTORC1/AMPK-BRCA1/BARD1 pathway and cholesterol metabolism in HC-stimulated AML12 cells. qPCR assay indicated that ASGR1 mRNA levels were significantly increased by 1–4 µg ASGR1 plasmid relative to controls (All the *p*-values are lower than 0.0001), there was no significant difference between 3 µg and 4 µg (*p* = 0.52), and the pcDNA3.1(+) group showed no notable change (*p* > 0.99) (Fig. 8A). Hence, subsequent experiments were performed using 3 µg of ASGR1 plasmid. Compared to the HC + Fis group, the HC + Fis+OE-ASGR1 group showed a reversal of the fisetin-mediated down-regulation of ASGR1 protein (*p* < 0.0001) (Fig. 8B-C). Similarly, OE-ASGR1 treatment effectively counteracted the inhibitory effect of fisetin on the increase in TC and TG levels induced by HC (Fig. 8D-E). Furthermore, the fisetin-inhibited mTORC1/AMPK-BRCA1/BARD1 pathway was reactivated in the Fis + OE-ASGR1 group. The levels of phosphorylated-mTOR, phosphorylated-S6K, BRCA1 and BARD1 were notably upregulated (all *p* < 0.0001), while phosphorylated-AMPK was downregulated in the Fis + OE-ASGR1 group (*p* < 0.0001) (Fig. 8F-K). Additionally, OE-ASGR1 treatment reversed the fisetin-induced upregulation of LXRα, ABCG5, and ABCG8 proteins (*p* < 0.01) (Fig. 8F, L-N). These results further confirm fisetin alleviate HC-induced cholesterol metabolism disorder through the ASGR1-mTORC1/AMPK-BRCA1/BARD1 pathway, along with a potential stimulation of cholesterol efflux.

### Synthesis and characterization of β-CD-Fis-CMCS

**Fig. 9:**
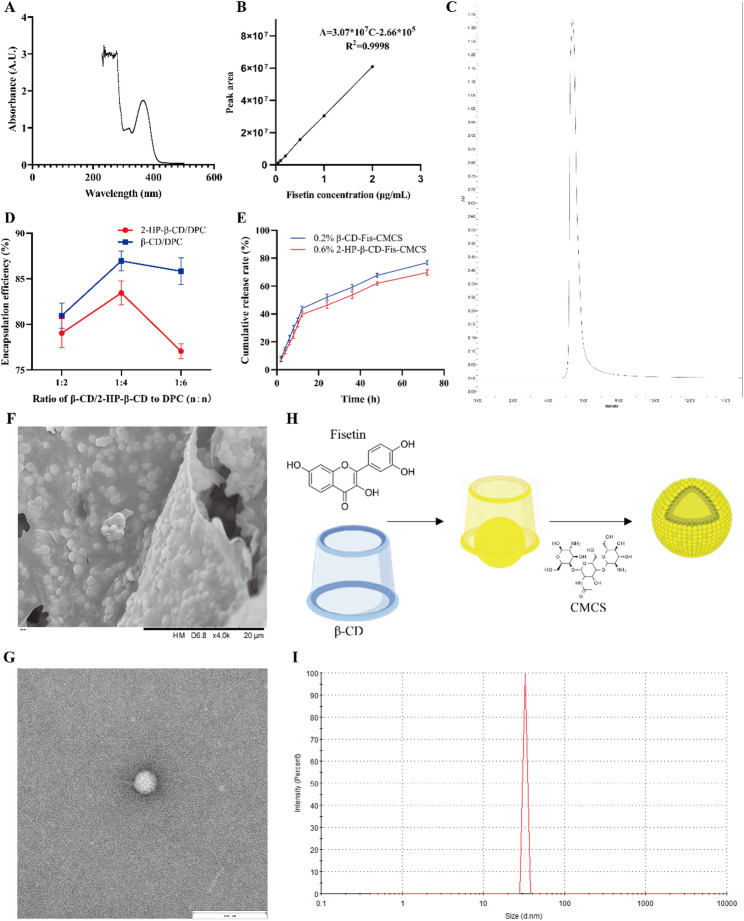
Fabrication and characterization of β-CD-Fis-CMCS. (**A**) UV–vis absorption spectra of fisetin. (**B**) Standard curve for fisetin. (**C**) HPLC of fisetin. (**D**) Line plots of nanoparticles encapsulation efficiency at different molar ratios. (**E**) The cumulative release curves of fisetin nanoparticles. (**F**) SEM images of β-CD-Fis-CMCS. Bar = 20 μm. (**G**) TEM images of β-CD-Fis-CMCS. Bar = 100 nm. (**H**) Schematic preparation process of β-CD-Fis-CMCS. (**I**) Dynamic light scattering analysis of β-CD-Fis-CMCS. Data are mean ± SD, *n* = 3. * *p* < 0.05, ** *p* < 0.01

To address the poor solubility, short half-life, and low bioavailability of fisetin, nanoparticles loaded with fisetin were developed. A full-spectrum UV scan was conducted to determine the maximum absorption wavelength of fisetin. The results indicated that fisetin exhibits peak absorption at 238 nm and 364 nm. However, the 238 nm wavelength is near the terminal absorption range, where potential interferences, such as solvent effects, may occur. Therefore, the maximum UV absorption wavelength of fisetin was determined to be 364 nm (Fig. 9A). Subsequently, an in vitro standard curve for fisetin was constructed (Fig. 9B, C). 2-HP-β-CD and β-CD, as high-quality nanocarriers, exhibit distinct characteristics [27]. To identify the optimal nanocarrier for fisetin, a series of experiments were conducted. Initially, the effects of varying molar ratios of 2-HP-β-CD/β-CD and DPC on the encapsulation efficiency and drug loading capacity of the nanoparticles were investigated. The results showed that the highest encapsulation efficiency and drug loading were achieved at a molar ratio of 1:4 (Fig. 9D; Table 4). Subsequently, the influence of varying CMCS concentrations on the particle size and zeta potential of the nanoparticles was evaluated. As shown in Table 5, the zeta potential reached its minimum when the CMCS concentration was 0.2% in β-CD. In contrast, for 2-HP-β-CD, the zeta potential decreased continuously with increasing CMCS concentration, reaching its minimum at 0.6%. Based on the combined analysis of particle size, zeta potential, and the polydispersity index (PDI), the optimal CMCS concentrations for 2-HP-β-CD and β-CD were determined to be 0.6% and 0.2%, respectively. Then, the release kinetics of fisetin from various fisetin nanoparticles were studied under conditions mimicking simulated small intestinal fluid. The cumulative drug release profiles for both formulations exhibited a rapid increase during the initial 12 h. However, over the subsequent 60-h period, the cumulative release rate of 0.2% β-CD-Fis-CMCS was significantly higher than that of 0.6% 2-HP-β-CD-Fis-CMCS (Fig. 9E). Therefore, β-CD was identified as the optimal nanocarrier for fisetin. The preparation procedure of the β-CD-Fis-CMCS is illustrated in Fig. 9H. Subsequently, the microscopic morphology of β-CD-Fis-CMCS was observed. SEM images revealed a homogeneous distribution of nanoparticles (Fig. 9F). TEM analysis showed that the β-CD-Fis-CMCS exhibited a spherical morphology with an average diameter of 40 ± 16 nm (Fig. 9G). Dynamic light scattering measurements indicated that the hydrodynamic diameter of fisetin nanoparticles peaked at 32 nm (Fig. 9I).

**Table 4:** Encapsulation efficiency and drug loading at various molar ratios

β-CD: DPC	EE (%)	DL (%)	2-HP-β-CD: DPC	EE (%)	DL (%)
1:2	80.96	18.89	1:2	79.03	20.49
1:4	86.96	19.70	1:4	83.44	21.47
1:6	85.83	19.38	1:6	77.06	19.27

**Table 5:** Particle size and zeta potential at various CMCS concentration

CMCS concentration (β-CD, w/v)	Particle size (nm)	Zeta potential (mV)	PDI	CMCS concentration (2-HP-β-CD, w/v)		Particle size (nm)	Zeta potential (mV)	PDI
0	78.6	-21.9	0.18	0	83.7		-21.7	0.14
0.1%	85.1	-24.7	0.15	0.1%	80.6		-25.4	0.18
0.2%	43.5	-32.7	0.14	0.2%	81.3		-26.9	0.16
0.4%	57.3	-28.4	0.19	0.4%	75.4		-29.6	0.19
0.6%	81.4	-26.1	0.17	0.6%	56.8		-33.3	0.11

### Biodistribution of β-CD-CMCS in mice

**Fig. 10:**
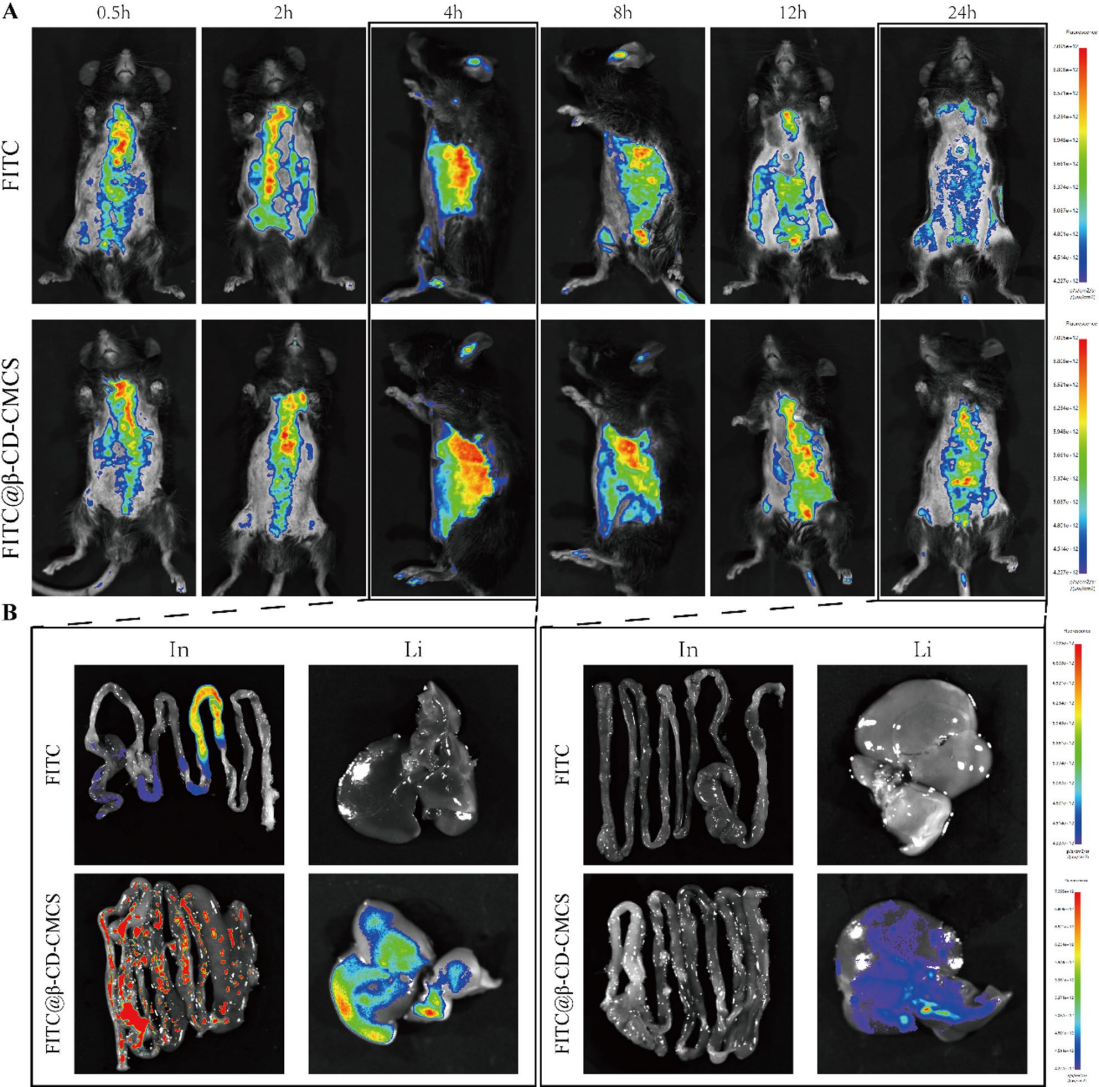
Biodistribution of β-CD-CMCS were evaluated by fluorescence imaging in vivo. (**A**) Representative time-dependent fluorescence images of healthy mice after administration of FITC@β-CD-CMCS or free FITC dye. (**B**) Fluorescence imaging in the intestine (In) and liver (Li) at 4 h and 24 h ex vivo

We evaluated the biodistribution and degradation of β-CD-CMCS in comparison to free FITC dye before treatment with β-CD-Fis-CMCS. FITC@β-CD-CMCS and free FITC dye were gavaged to healthy C57BL/6J mice. In the free FITC group, fluorescence was predominantly observed along the digestive tract, with intensity gradually diminishing over time and becoming undetectable after 24 h. In contrast, FITC@β-CD-CMCS rapidly accumulated in the liver and digestive tract within 2–4 h, with fluorescence signals persisting beyond 24 h (Fig. 10A). Quantitative fluorescence analysis of major organs, particularly the liver, further demonstrated the effective liver-targeting capability of β-CD-CMCS (Fig. 10B).

### β-CD-Fis-CMCS regulated cholesterol metabolism in the HC-induced AML12 cells

**Fig. 11:**
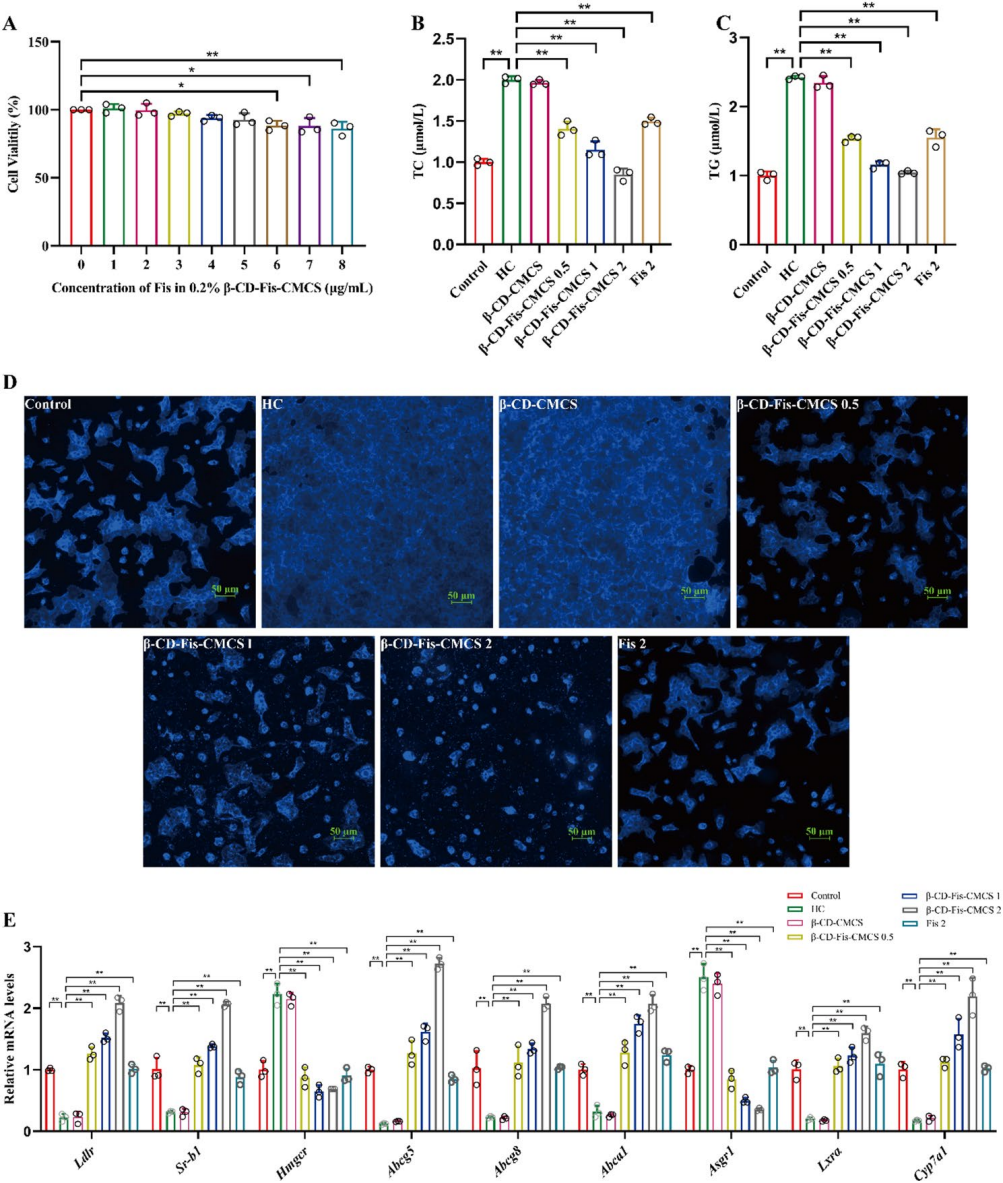
Effect of β-CD-Fis-CMCS cholesterol metabolism in HC-induced AML12 cells. (**A**) The cell viability of AML12 cells. (**B**) TC levels. (**C**) TG levels. (**D**) Filipin Ⅲ staining. Scale bar (50 μm), magnification (200 ×). (**E**) Quantification of mRNA levels for key cholesterol metabolism genes. Data are mean ± SD, *n* = 3. * *p* < 0.05, ** *p* < 0.01

The toxicity of β-CD-Fis-CMCS in AML12 cells was evaluated. CCK-8 assay results demonstrated that β-CD-Fis-CMCS did not significantly reduce cell viability at concentrations below 6 µg/mL (*p* > 0.05) (Fig. 11A). Subsequently, β-CD-Fis-CMCS was applied for further investigation into their effects on cholesterol metabolism. Both the HC and HC + β-CD-CMCS groups had markedly higher levels of TC and TG than the control group (*p* < 0.0001), with no significant differences between them (*p* > 0.05). However, treatment with β-CD-Fis-CMCS markedly reduced TC and TG levels (Fig. 11B, C). Additionally, cholesterol accumulation was significantly more pronounced in the HC and HC + β-CD-CMCS groups compared to the HC + β-CD-Fis-CMCS group (Fig. 11D). As shown in Fig. 11E, compared to the control group, *Ldlr*, *Sr-b1*, *Abcg5*, *Abcg8*, *Abca1*,* Lxrα* and *Cyp7a1* mRNA expression levels were markedly downregulated in the experimental group, while *Hmgcr* and *Asgr1* were remarkably upregulated (*p* < 0.0001). However, β-CD-Fis-CMCS administration successfully restored the expression of these genes to normal levels. Notably, as illustrated in Fig. 11B-E, the treatment effect of the β-CD-Fis-CMCS (0.5 µg/mL of fisetin) was comparable to, or even surpassed, that of fisetin (2 µg/mL). Together, β-CD-Fis-CMCS effectively mitigates the cytotoxic effects of fisetin and significantly alleviates HC-induced cholesterol metabolism disorders even at one-fourth the concentration of free fisetin.

### β-CD-Fis-CMCS enhanced the efficacy of Fisetin in enhancing hepatic cholesterol efflux in mice

**Fig. 12:**
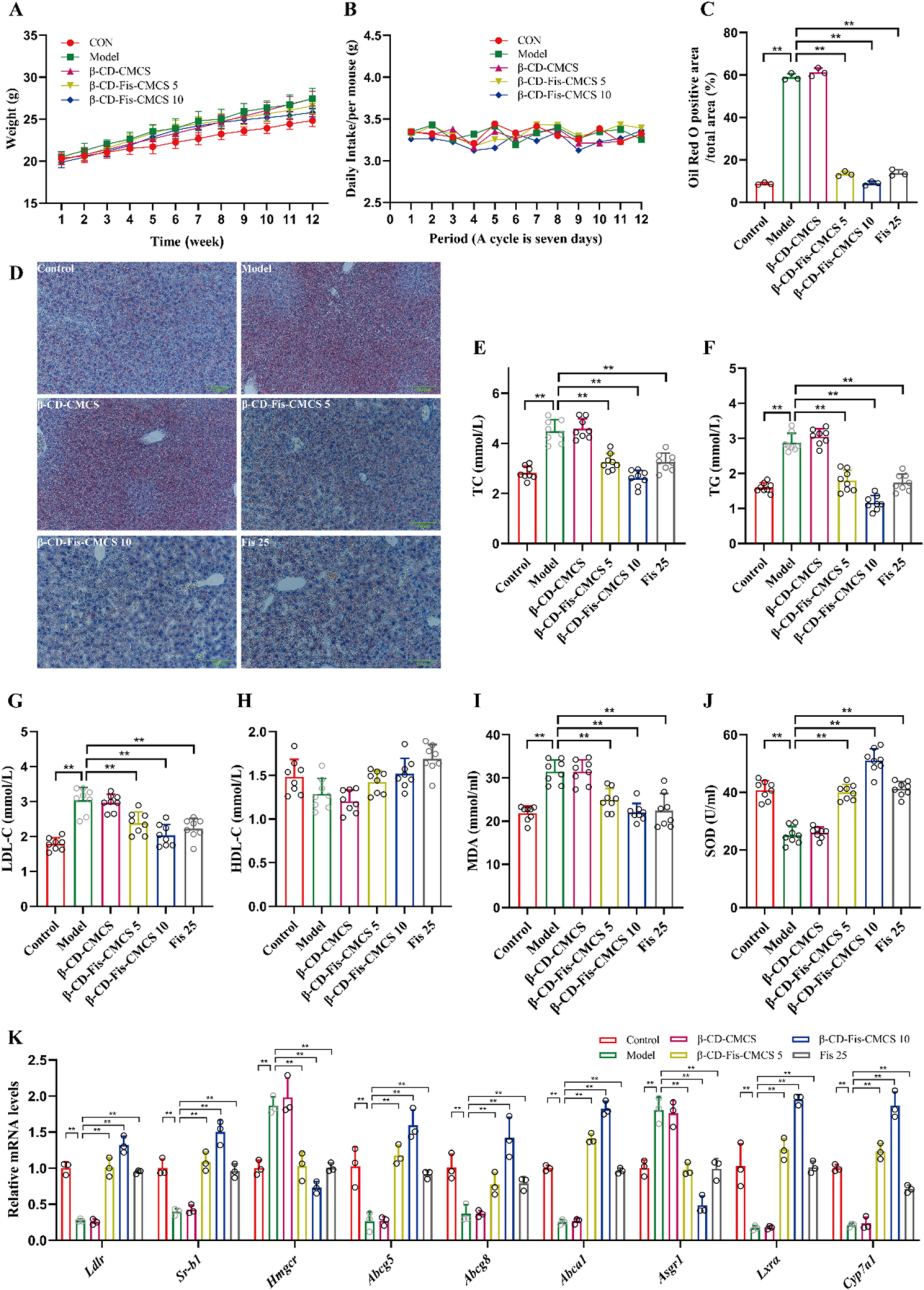
β-CD-Fis-CMCS alleviated hepatic cholesterol accumulation in HFHC-induced mice. (**A**) Body weight. (**B**) Daily food intake per mouse throughout the intervention period. (**C**) Quantitative analysis of ORO-positive area in C57BL/6J mice liver. (**D**) Representative images of ORO staining in liver tissues. (magnification, 200 ×). (**E**) Levels of serum TC. (**F**) Levels of serum TG. (**G**) Levels of serum LDL-C. (**H**) Levels of serum HDL-C. (**I**) Serum MDA levels. (**J**) Serum SOD activity. (**K**) Quantification of mRNA levels for key cholesterol metabolism genes. Data are mean ± SD, *n* = 8 or 3. * *p* < 0.05, ** *p* < 0.01

Consistent with the modeling method described in methods and materials, mice were gavaged with β-CD-Fis-CMCS containing 5 mg/kg and 10 mg/kg of fisetin. Throughout the experiment, body weight in all groups increased steadily, and daily food intake remained stable (Fig. 12A, B). Following 8 weeks of β-CD-Fis-CMCS treatment, HFHC-induced lipid accumulation was significantly alleviated (Fig. 12C, D). After 12 weeks of HFHC diet feeding, serum TC, TG and LDL-C levels were significantly increased relative to the control group (*p* < 0.01). Notably, β-CD-Fis-CMCS treatment significantly reversed these changes (*p* < 0.0001), while no significant differences were observed in serum HDL-C levels across all groups (*p* > 0.05) (Fig. 12E-H). Relative to controls, the experimental group exhibited elevated MDA levels alongside suppressed SOD activity (*p* < 0.0001). β-CD-Fis-CMCS treatment effectively attenuated these HFHC-induced alterations (Fig. 12I-J). Key genes involved in cholesterol metabolism were modulated by β-CD-Fis-CMCS treatment. Specifically, *Ldlr*, *Sr-b1*, *Abcg5*, *Abcg8*, *Abca1*, *Lxrα* and *Cyp7a1* were significantly increased, whereas *Hmgcr* and *Asgr1* were decreased relative to the model group (all *p* < 0.0001) (Fig. 12K). In Fig. 12C-K, no statistically significant difference was observed between β-CD-Fis-CMCS (5 mg/kg of fisetin) and fisetin (25 mg/kg). These results demonstrate that β-CD-Fis-CMCS effectively reduce hepatic lipid accumulation in HFHC-fed mice. The therapeutic efficacy of β-CD-Fis-CMCS was maintained even at one-fifth the dose of fisetin, highlighting its potential as a powerful intervention for hypercholesterolemia.

## Discussion

ASCVD is a major cause of morbidity and mortality globally [28]. Extensive research has identified hypercholesterolemia as a critical risk factor driving the onset and progression of ASCVD [29]. Despite their broad use in reducing cardiovascular events, statins are limited by side effects, prompting the search for alternative therapies [30]. Natural compounds, such as fisetin, have garnered increasing attention due to their abundant availability and low toxicity profiles. This study demonstrates that treatment with fisetin and β-CD-Fis-CMCS significantly reduced hepatic lipid accumulation, lowered lipid levels, and attenuated lipid peroxidation in C57BL/6J mice and AML12 hepatocyte cells. Notably, β-CD-Fis-CMCS exhibited reduced cytotoxicity alongside superior therapeutic efficacy compared to free fisetin. Mechanistic investigations revealed that fisetin modulates cholesterol metabolism by acting on ASGR1 and activating the mTORC1/AMPK-BRCA1/BARD1 signaling axis, which may ultimately promote hepatobiliary cholesterol excretion. These findings offer novel insights into the therapeutic potential of fisetin for hypercholesterolemia management.

Fisetin, a natural flavonoid present in various fruits and vegetables, possesses antilipemic, antioxidant and anticancer activities. We previously identified that fisetin mitigates hypercholesterolemia by enhancing cholesterol secretion through the transintestinal cholesterol excretion (TICE) pathway [31]. However, the precise mechanisms of fisetin in regulating cholesterol metabolism, particularly through the RCT pathway, remain to be fully elucidated. In this study, C57BL/6J mice and AML12 cells were utilized to evaluate the efficacy of fisetin in preventing hypercholesterolemia. Our findings revealed substantial lipid accumulation and elevated lipid levels in both HFHC-induced C57BL/6J mice and HC-treated AML12 cells. Fisetin treatment significantly inhibited lipid deposition, ameliorated disrupted lipid metabolism, and reduced TC, TG and LDL-C levels. Additionally, fisetin appeared to slightly increase HDL-C levels, but no significant difference was observed between groups, which may be attributed to individual variability among the mice. Collectively, these findings support the therapeutic potential of fisetin against lipid metabolic disorders and hypercholesterolemia. Oxidative stress is crucial to the pathogenesis of hypercholesterolemia, making antioxidant therapy a promising strategy for its management [32]. For instance, antarctic krill oil has been shown to alleviate HFD-induced hypercholesterolemia by mitigating oxidative stress and modulating related molecular pathways [33]. In our study, the model group exhibited elevated MDA levels and reduced SOD activity, indicative of oxidative damage. Notably, fisetin treatment effectively suppressed MDA levels while enhancing SOD activity. This antioxidant capacity of fisetin likely contributes to its protective effects against hypercholesterolemia.

The liver plays a central role in cholesterol metabolism and maintains cholesterol homeostasis primarily by taking up cholesterol from LDL particles *via* LDLR-mediated endocytosis [34]. Degradation of LDLR within hepatocyte lysosomes results in elevated circulating LDL-C levels [35]. As expected, fisetin treatment significantly upregulated LDLR expression. SR-B1 mediates the selective uptake of HDL-derived cholesteryl esters by the liver. Research has demonstrated that flavonoids can modulate lipid metabolism by regulating SR-B1 expression [17, 36]. In this study, fisetin intervention significantly upregulated both gene and protein expression levels of SR-B1, thereby promoting RCT and reducing cholesterol accumulation relative to the model group. HMGCR serves as a critical enzyme for cholesterol biosynthesis. Fisetin has been shown to significantly downregulate hepatic HMGCR at both gene and protein expression levels, thus inhibiting hepatic cholesterol synthesis. Cholesterol excretion is crucial for the liver’s ability to eliminate free cholesterol. CYP7A1 converts hepatic cholesterol to bile acids, which is a major route of cholesterol excretion. Impaired CYP7A1 function is associated with elevated plasma cholesterol levels [37]. Several studies have demonstrated that phytochemicals can alleviate hypercholesterolemia by upregulating CYP7A1 activity, thereby enhancing bile acid synthesis and facilitating cholesterol clearance [38, 39]. Consistent with these findings, fisetin intervention significantly increased CYP7A1 expression, likely by facilitating cholesterol excretion and thereby reducing cholesterol levels. As a nuclear receptor protein, LXR facilitates cholesterol efflux and is a crucial therapeutic target for atherosclerosis [40]. Li et al. reported that compounds like qingre sanjie formula activated LXRα expression and enhanced the expression of the downstream targets, CYP7A1 and ABCG5/8, thereby facilitating cholesterol excretion [41]. Similarly, fisetin was found to significantly increase both protein and gene expression levels of LXRα compared to the model group. Furthermore, hepatobiliary cholesterol secretion into bile is the primary pathway for cholesterol elimination. ABCA1, ABCG5, and ABCG8 are critical ABC transporters involved in cholesterol regulation. ABCA1 mediates efflux of cholesterol, loading into lipid-poor ApoA-I to form HDL, which facilitates the RCT process [42]. ABCG5 and ABCG8 are key transporters for transferring cholesterol from hepatocytes into bile. Previous studies have demonstrated that upregulation of *Abcg5* and *Abcg8* gene expression enhances cholesterol secretion and bile formation [43]. Additionally, Lien et al. revealed that hawthorn leaf flavonoids mitigate HFHC-induced atherosclerosis by upregulating ABCG5/ABCG8 expression, thus promoting cholesterol excretion [44]. In line with these findings, fisetin markedly increased ABCA1, ABCG5 and ABCG8 protein and gene levels both in vitro and in vivo. Therefore, by ameliorating cholesterol metabolism and potentially facilitating cholesterol elimination through modulating the RCT pathway, fisetin may exert a protective effect against hypercholesterolemia.

As a transmembrane protein expressed on the surface of hepatocytes, ASGR1 mediates the endocytosis and lysosomal degradation of asialoglycoprotein circulating in the bloodstream [45]. This process is crucial for various pathophysiological mechanisms [46–48]. Numerous studies have revealed that deficiency of ASGR1 is linked to a marked reduction in lipid levels [49, 50]. Notably, Svecla et al. reported that ASGR1 deficiency significantly lowered plasma lipid concentrations and attenuated the development of ASCVD in hypercholesterolemic ApoE^-/-^ mice [51]. Coincidentally, our findings suggest that ASGR1 could be a potential target of fisetin. Molecular docking and CETSA assays indicated a potential interaction between fisetin and ASGR1. Further experimental evidence showed that fisetin effectively suppressed both the protein and gene expression levels of ASGR1. Immunofluorescence analysis further revealed that HC treatment significantly increased the co-localization of ASGR1 with lysosomal marker CD63 in AML12 cells, whereas fisetin treatment markedly diminished this co-localization. Concurrently, fisetin administration markedly reduced ASGR1 fluorescence intensity relative to the HC group. These results strongly demonstrate that ASGR1 is a key molecular target through which fisetin promotes cholesterol metabolism and efflux. mTORC1 and AMPK sense lysosomal degradation of nutrients and regulate lipid metabolism, working in opposition through multiple mechanisms [52, 53]. Inhibition of the mTORC1/AMPK axis enhanced the activity of the BRCA1/BARD1 ubiquitin ligase (E3) complex, thus promoting cholesterol efflux [54]. Consistent with these findings, our study demonstrated that fisetin treatment significantly enhanced AMPK phosphorylation while inhibiting the activation of mTORC1 and the BRCA1/BARD1 complex. This suggests that fisetin may facilitate cholesterol efflux by modulating the ASGR1-mTORC1/AMPK-BRCA1/BARD1 signaling pathway. To further validate this mechanism, AML12 cells were pretreated with an OE-ASGR1 plasmid, which abrogated the fisetin-induced downregulation of ASGR1 and consequently inhibited the ASGR1-mTORC1/AMPK-BRCA1/BARD1 pathway. Furthermore, OE-ASGR1 reversed the effects of fisetin on HC-induced reductions in TC and TG. These results underscore the critical role of the ASGR1-mTORC1/AMPK-BRCA1/BARD1 axis in mediating fisetin’s beneficial effects on cholesterol metabolism regulation. Together, these findings not only provide novel insights into the molecular mechanisms underlying fisetin-mediated alleviation of hypercholesterolemia, but also establish a theoretical foundation for the development of targeted therapeutic strategies and preventive interventions.

Our studies have identified fisetin as a promising candidate for promoting cholesterol efflux and preventing hypercholesterolemia. However, poor water solubility and low bioavailability severely limit its clinical application [55]. To overcome these challenges, novel drug delivery systems are being developed to improve fisetin’s solubility and bioavailability [56]. The carrier material, as the core component of nanoparticles, determines the physicochemical properties, biocompatibility, and functional performance of the delivery system [57]. Natural carrier materials such as sugars, lipids, and proteins have garnered significant attention in biomedicine due to their safety, non-toxicity, biocompatibility, and biodegradability [58, 59]. β-CD, a cyclic oligomer composed of seven D(+)-pyranose glucose units, features a hydrophobic inner cavity that can encapsulate poorly soluble drugs, thereby enhancing their solubility and stability [60]. For instance, encapsulating piperine in β-CD-based nanosponges has been shown to improve both loading efficiency and bioactivity [61]. Similarly, oral colon-targeted chitosan/chondroitin sulfate nanoparticles loaded with fisetin have demonstrated significantly enhanced bioavailability and therapeutic efficacy [62]. CMCS, a water-soluble derivative of chitosan with excellent biocompatibility and biodegradability, exhibits unique biological properties that make it a highly promising material for drug delivery, bioimaging, biosensors, and gene therapy [63, 64]. In this study, to address the clinical challenges associated with fisetin, we optimized the formulation of fisetin nanoparticles. The resulting β-CD-Fis-CMCS exhibited favorable physicochemical characteristics, excellent biocompatibility, and a sustained release effect, which prolonged the in vivo retention time of fisetin. Both in vitro and in vivo experiments revealed that β-CD-Fis-CMCS significantly enhanced cholesterol-lowering efficacy compared to free fisetin treatment. Overall, the development of β-CD-Fis-CMCS nanoparticles significantly enhances the therapeutic potential of fisetin, offering a promising strategy for improving cholesterol-lowering efficacy and overcoming the clinical limitations of free fisetin.

There are still some limitations in the present study. (1) Limitations of the experimental model. While mouse cell models have provided valuable insights into the mechanisms of lipid metabolism, physiological differences between mice and humans may limit the direct applicability of the results. Specifically, mice lack the expression of cholesterol ester transporter (CETP). Therefore, the application of CETP transgenic mouse models, as well as higher-level experimental models such as human-derived stem cells and organoids, is preferable for achieving more accurate insights. Future studies can optimize this experimental model for data with higher clinical applicability. (2) Lack of liver-targeted agent conjugation for fisetin nanoparticles. Multivalent N-acetylgalactosamine (GalNAc) has been extensively employed for nanoparticle liver targeting due to its high affinity for asialoglycoprotein receptor [65, 66]. Therefore, the GalNAc conjugated fisetin nanoparticles may lead to more efficient uptake of fesetin by the liver. Future studies can optimize this experimental design for better therapeutic effects. (3) Limitations of the in vitro release model. This study examined the release characteristics of synthesized β-CD-Fis-CMCS in artificial colonic fluid (PBS with pH 7.4), demonstrating sustained drug release. However, actual colonic fluid contains additional components, such as bile salts and bile acids. Therefore, more accurate results may be achieved by employing a more physiologically relevant simulation of colonic fluid in future studies. Moreover, the physiological environments of the stomach and small intestine differ from that of the colon [67], potentially impacting the stability and release profile of nanoparticles. Therefore, future studies should consider integrating the in vitro release results of β-CD-Fis-CMCS in artificial gastric and small intestinal fluids for a more comprehensive understanding of the release profile of β-CD-Fis-CMCS within the body.

## Conclusions

In this study, we constructed a liver-targeted β-CD-Fis-CMCS and systematically demonstrated its enhanced cholesterol-lowering effects in a hypercholesterolemic mouse model. The results revealed that β-CD-Fis-CMCS effectively targeted hepatocytes, markedly alleviating hepatic lipid accumulation and oxidative stress. The underlying mechanism primarily involves the downregulation of ASGR1 expression, which inhibits the mTORC1/AMPK-BRCA1/BARD1 signaling axis, potentially promoting hepatic cholesterol efflux.

## Supplementary Information

Supplementary Material 1.

## Data Availability

Data is provided within the manuscript.

## Abbreviations

TC: Total cholesterol
TG: Triglycerides
LDL-C: Low-density lipoprotein cholesterol
ASGR1: Asialoglycoprotein receptor 1
CMCS: Carboxymethyl chitosan
β-CD: β-cyclodextrin
ASCVD: Atherosclerotic cardiovascular disease
RCT: Reverse cholesterol transport
ABCA1: ATP-binding cassette transporter A1
HDL: High-density lipoprotein
CYP7A1: Cholesterol 7α-hydroxylase
HMGCR: Hydroxy-methyl-glutaryl-coenzyme A reductase
MDA: Malondialdehyde
SOD: Superoxide dismutase
HFHC: High fat and high cholesterol
FBS: Fetal bovine serum
CCK-8: Cell counting kit-8
CETSA: Cellular thermal shift assay
CD63: Cluster of Differentiation 63
DPC: Diphenyl carbonate
HPLC: High-performance liquid chromatography
SEM: Scanning electron microscope
TEM: Transmission electron microscope
PCC: Pearson correlation coefficient
TICE: Transintestinal cholesterol excretion
CETP: Cholesterol transfer protein
