# Supplementary material for: Fisetin-loaded nanoparticles as a novel approach for cholesterol regulation in hypercholesterolemia: targeting the ASGR1-mediated mTORC1/AMPK pathway
Source: J Nanobiotechnology. 2026 Feb 27;24:312. doi: 10.1186/s12951-026-04181-z (PMC13050004; doi:10.1186/s12951-026-04181-z)

Fig. 3B

ABCA1
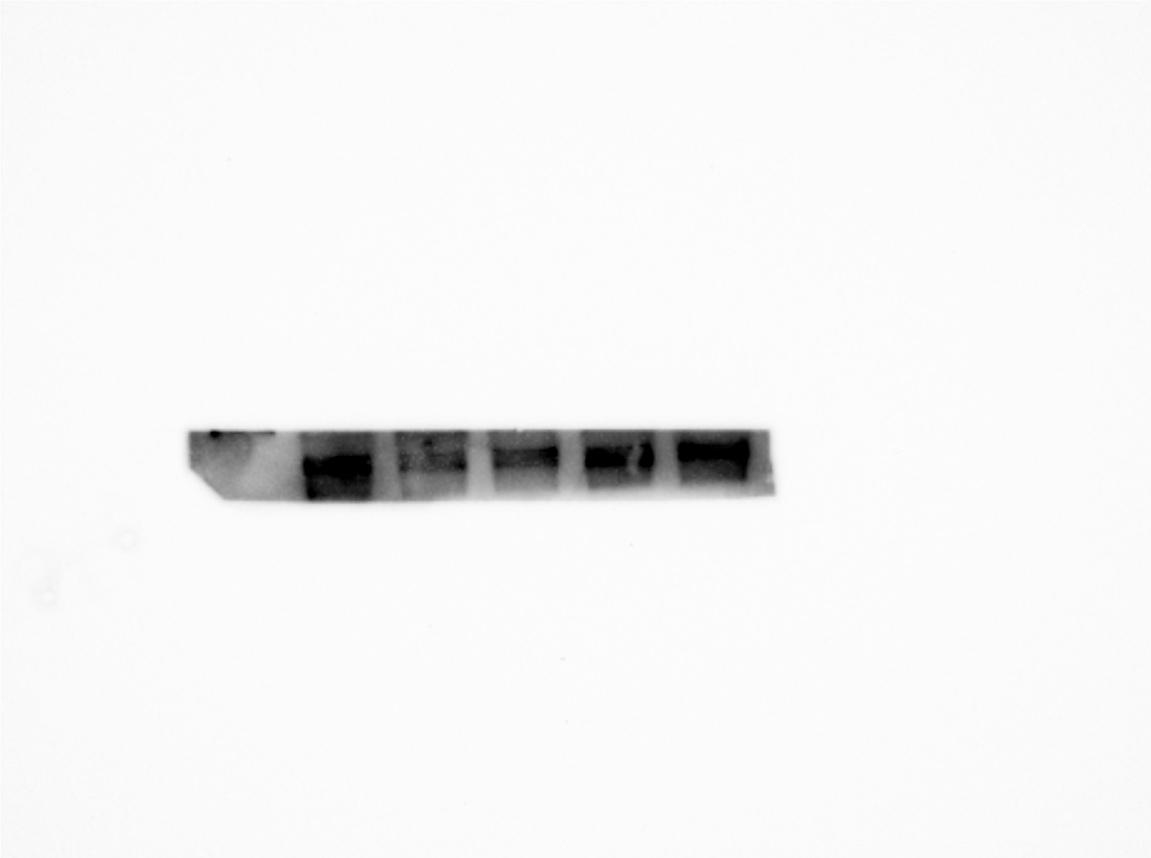


LDLR
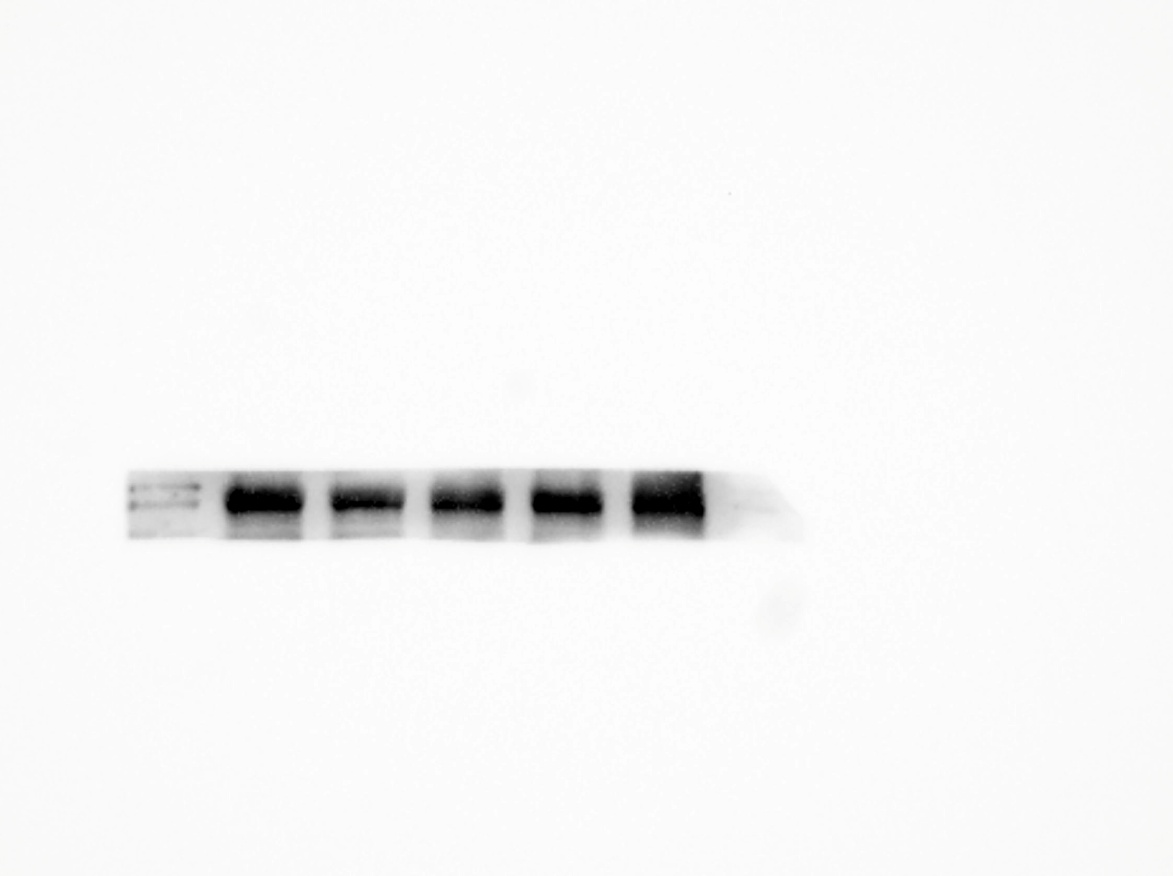


SR-B1
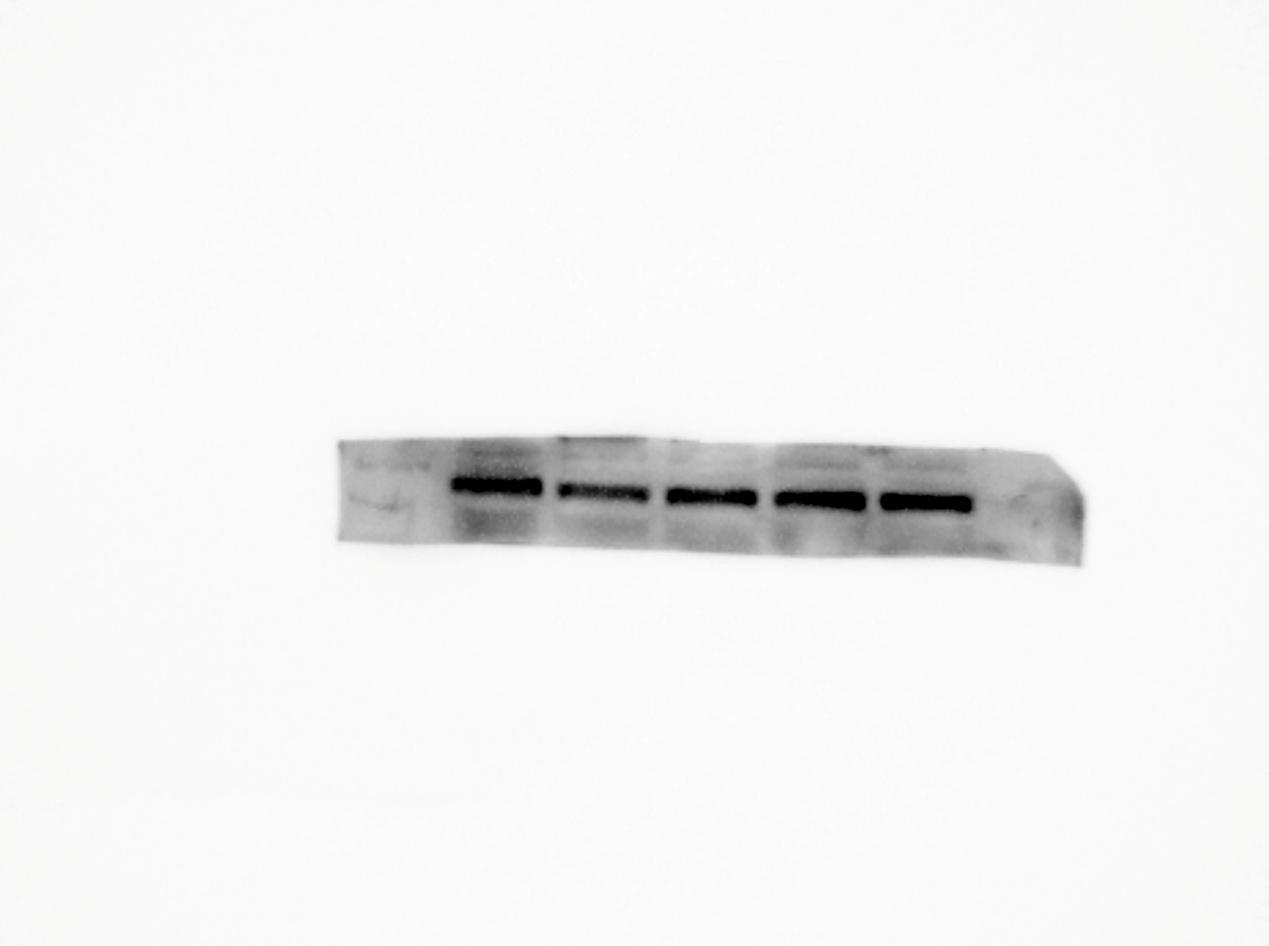


LXRα
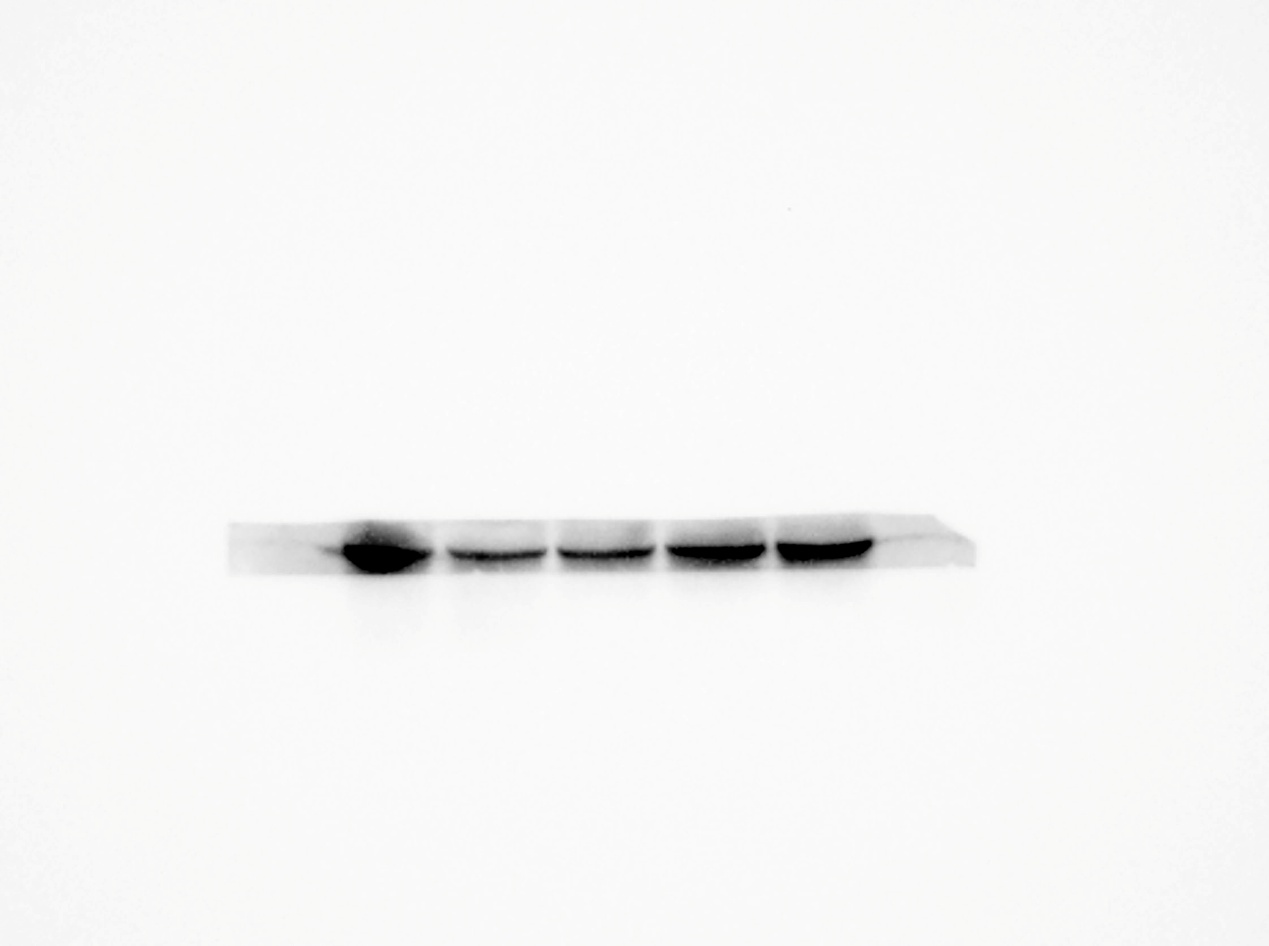


HMGCR
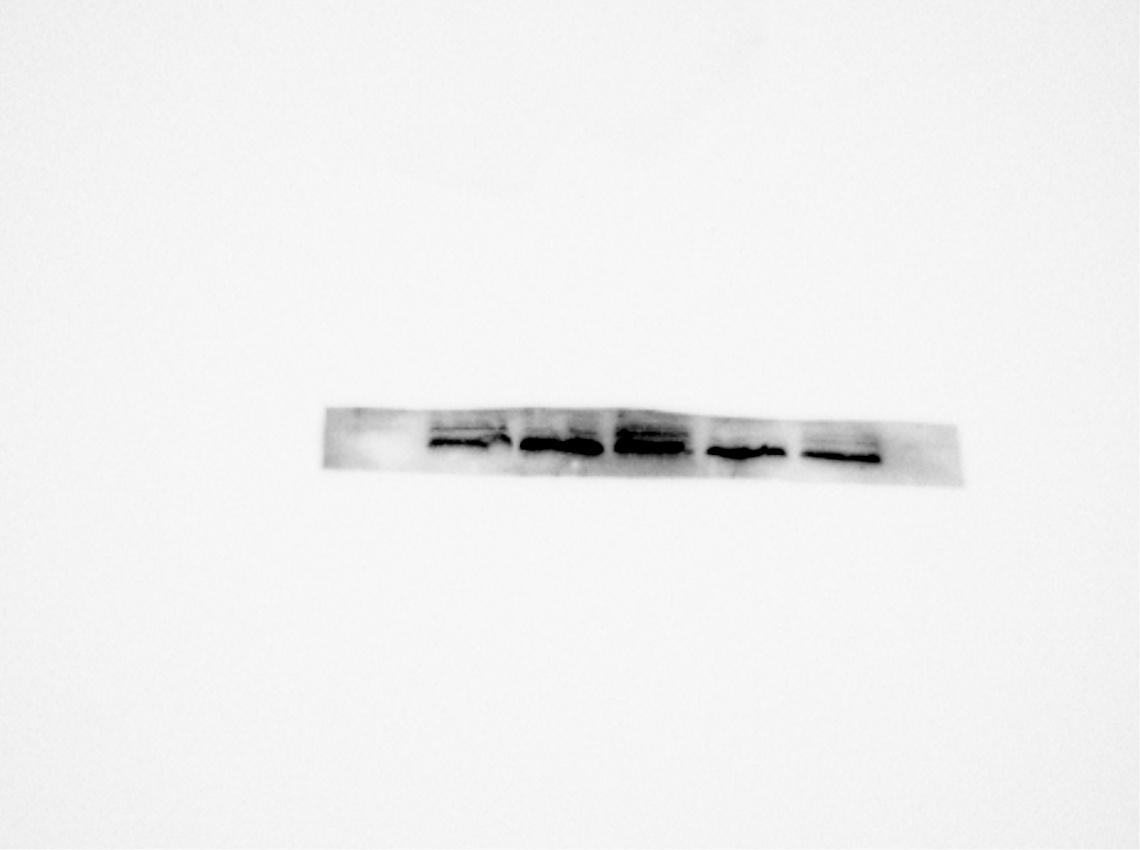


ABCG5
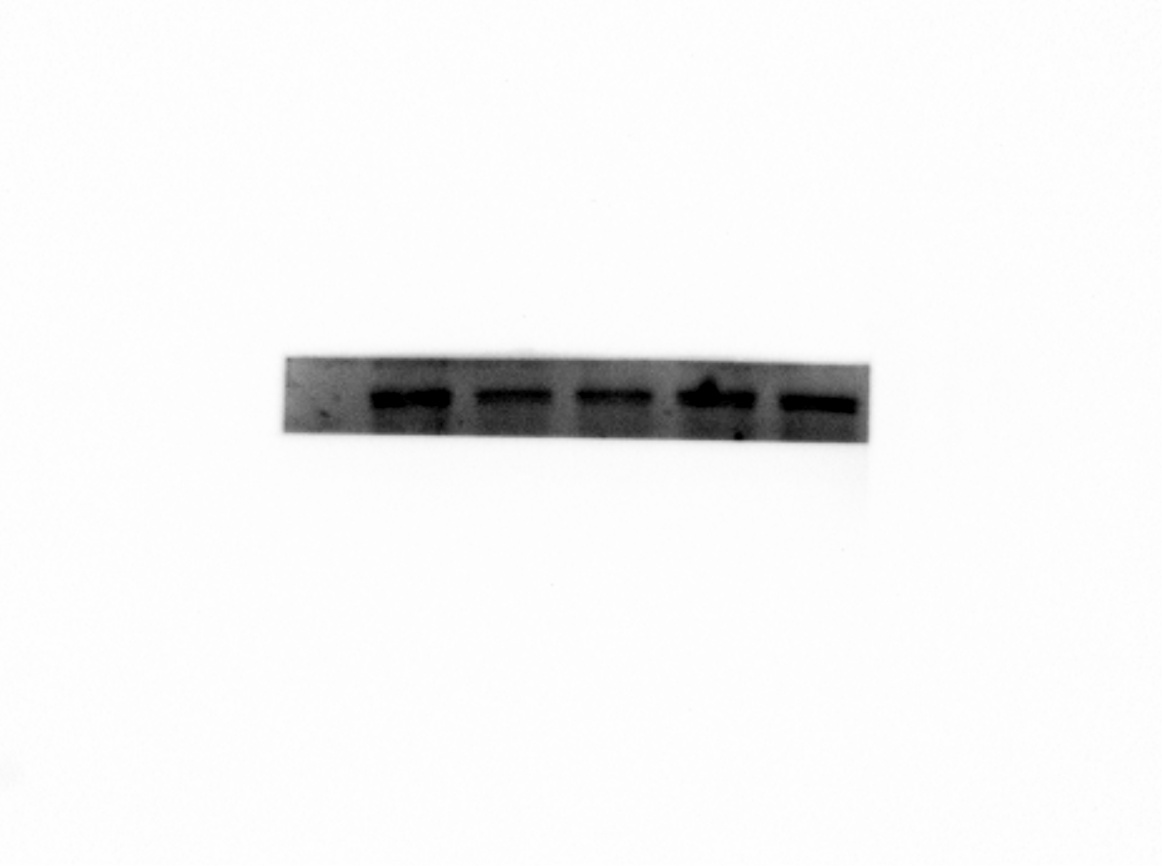


ABCG8
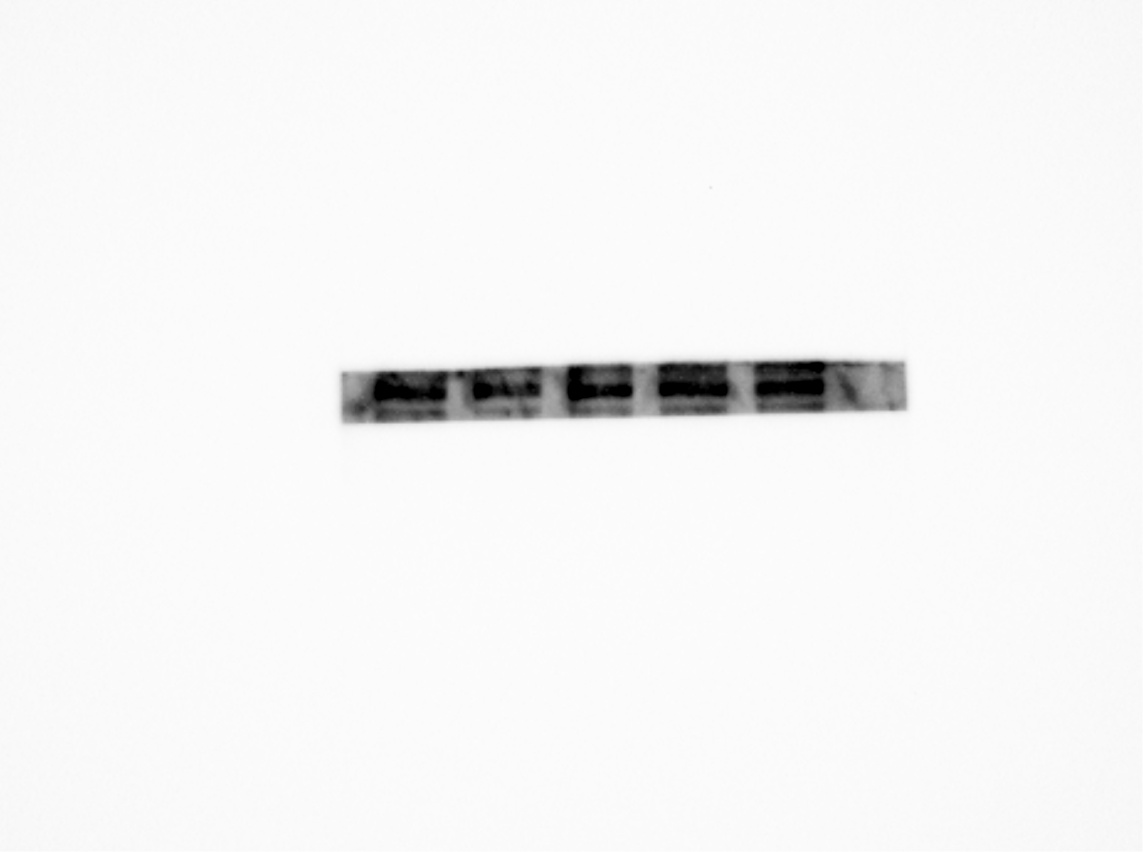


CYP7A1
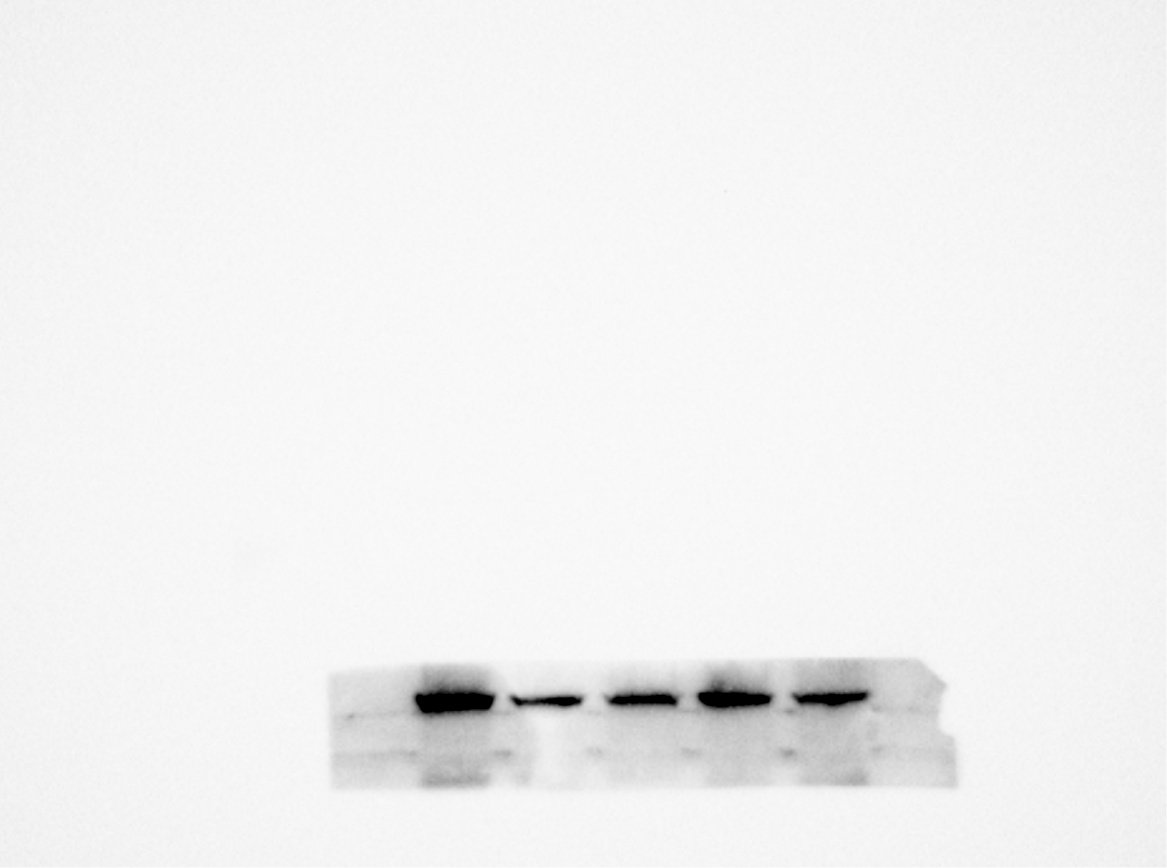


β-Actin
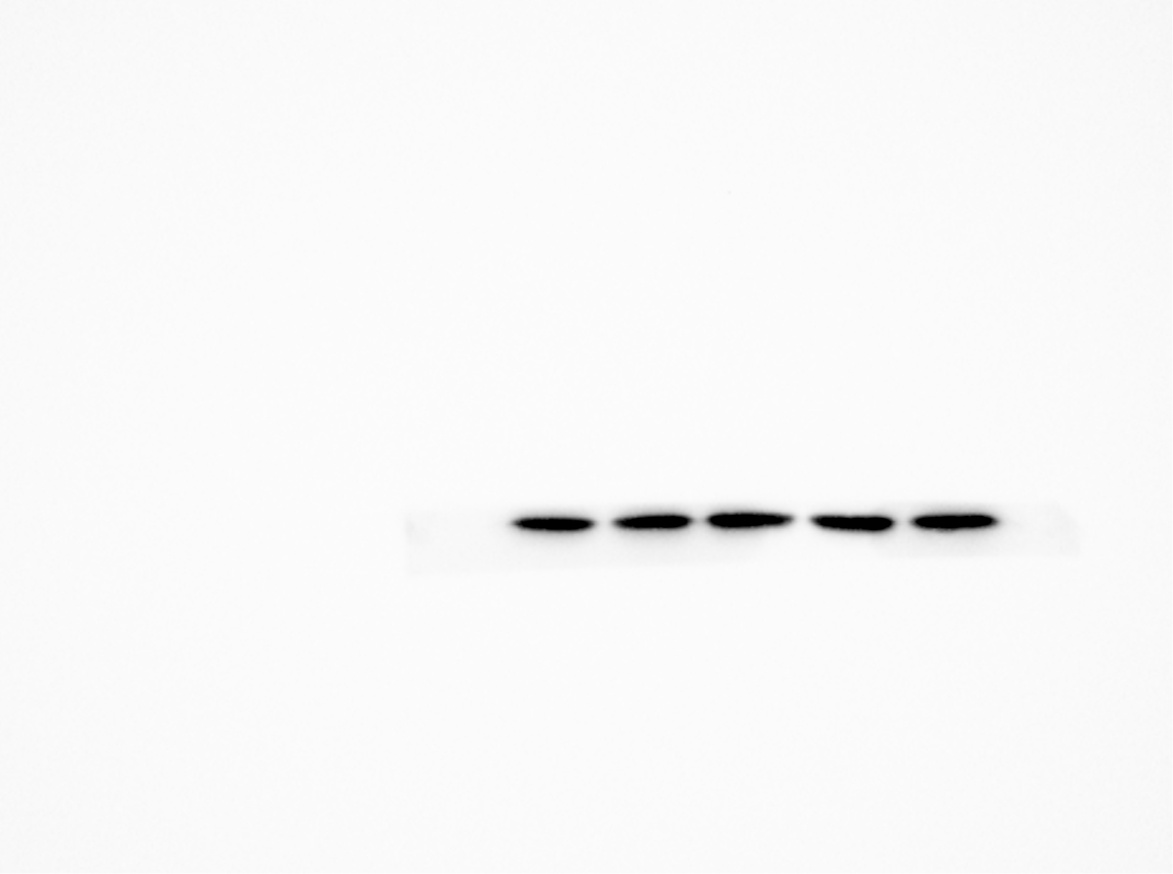


Fig. 4F

ABCA1
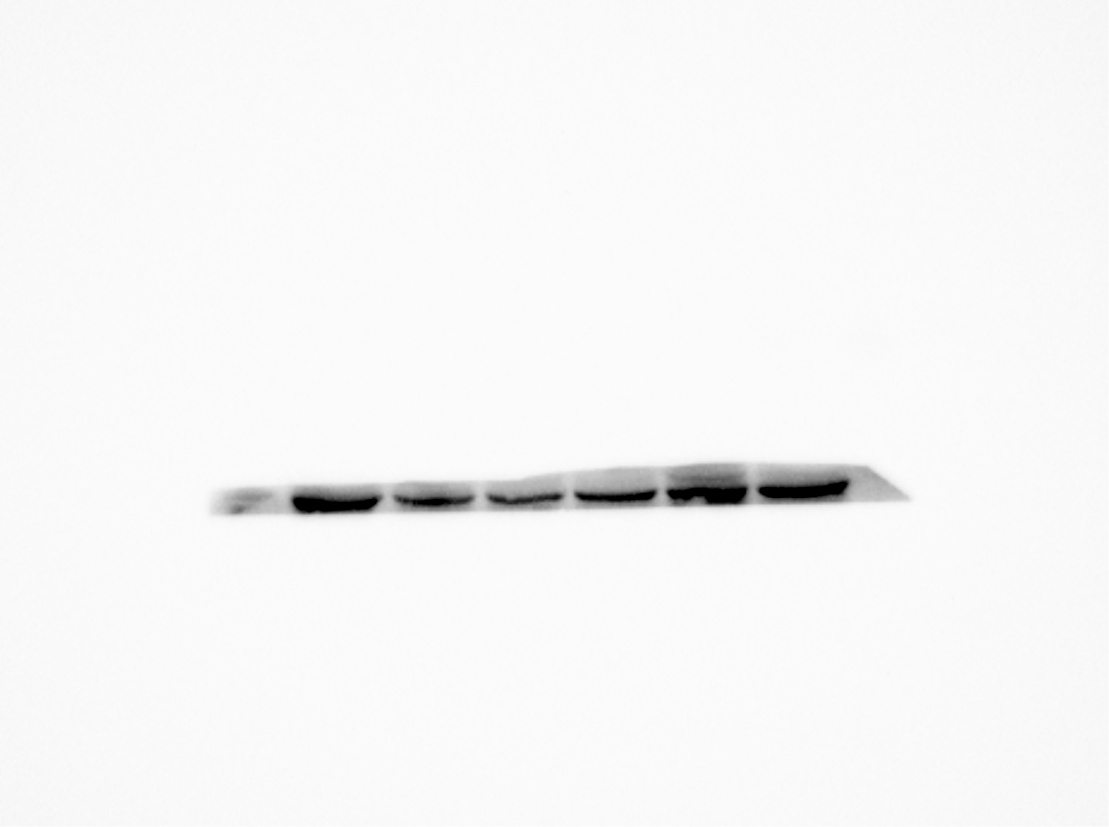


LDLR
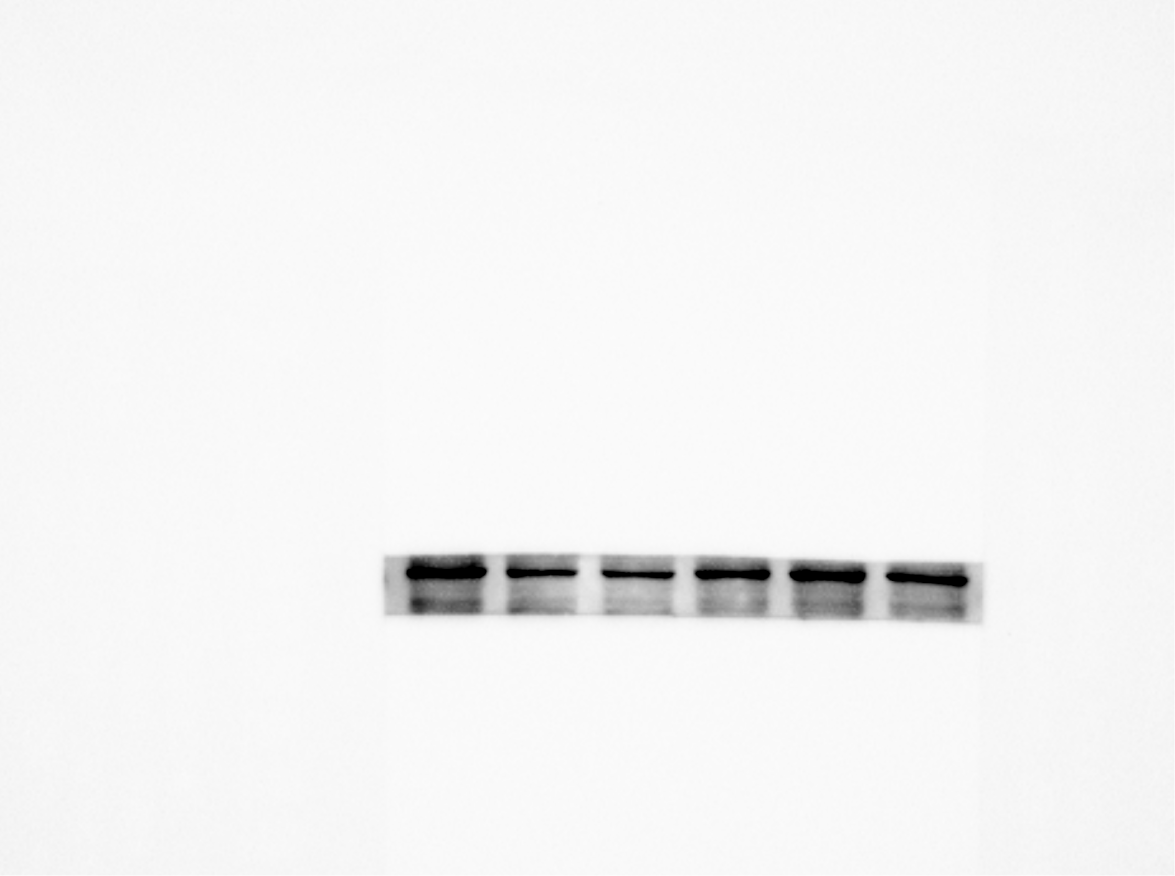


SR-B1
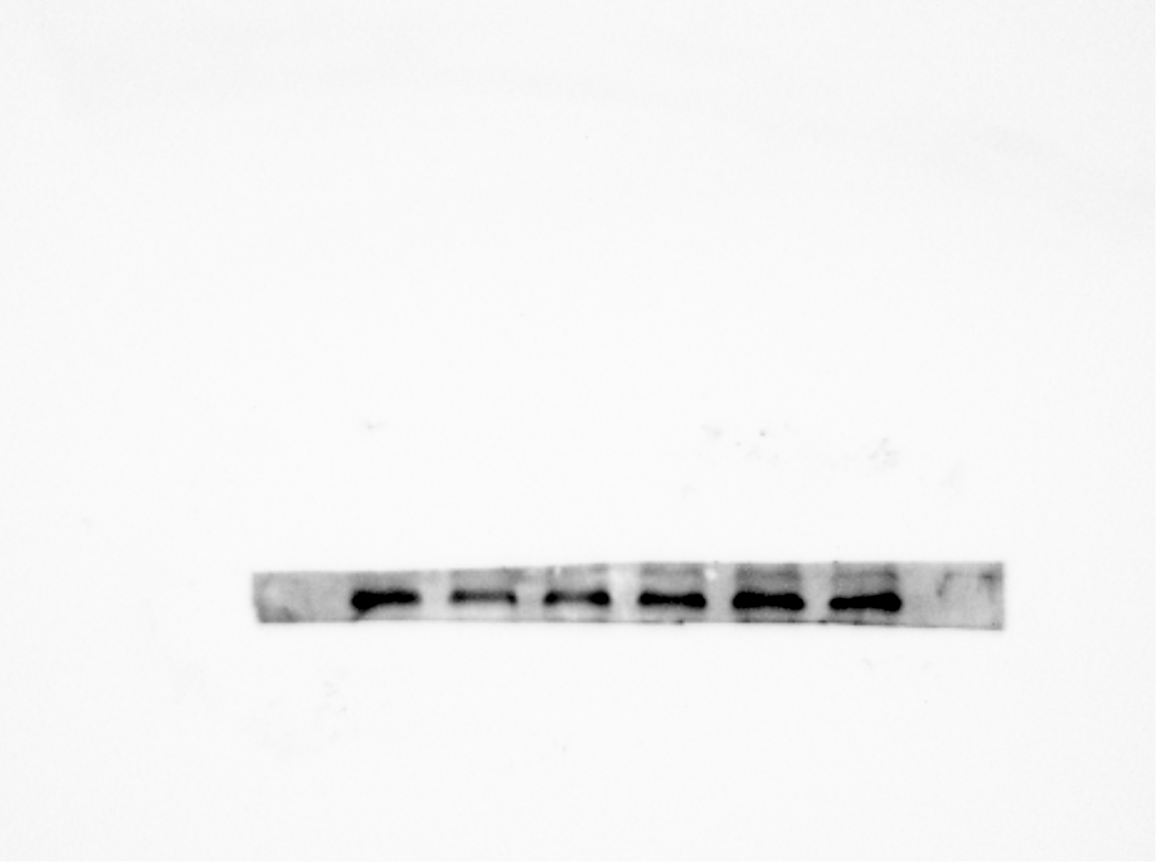


LXRα
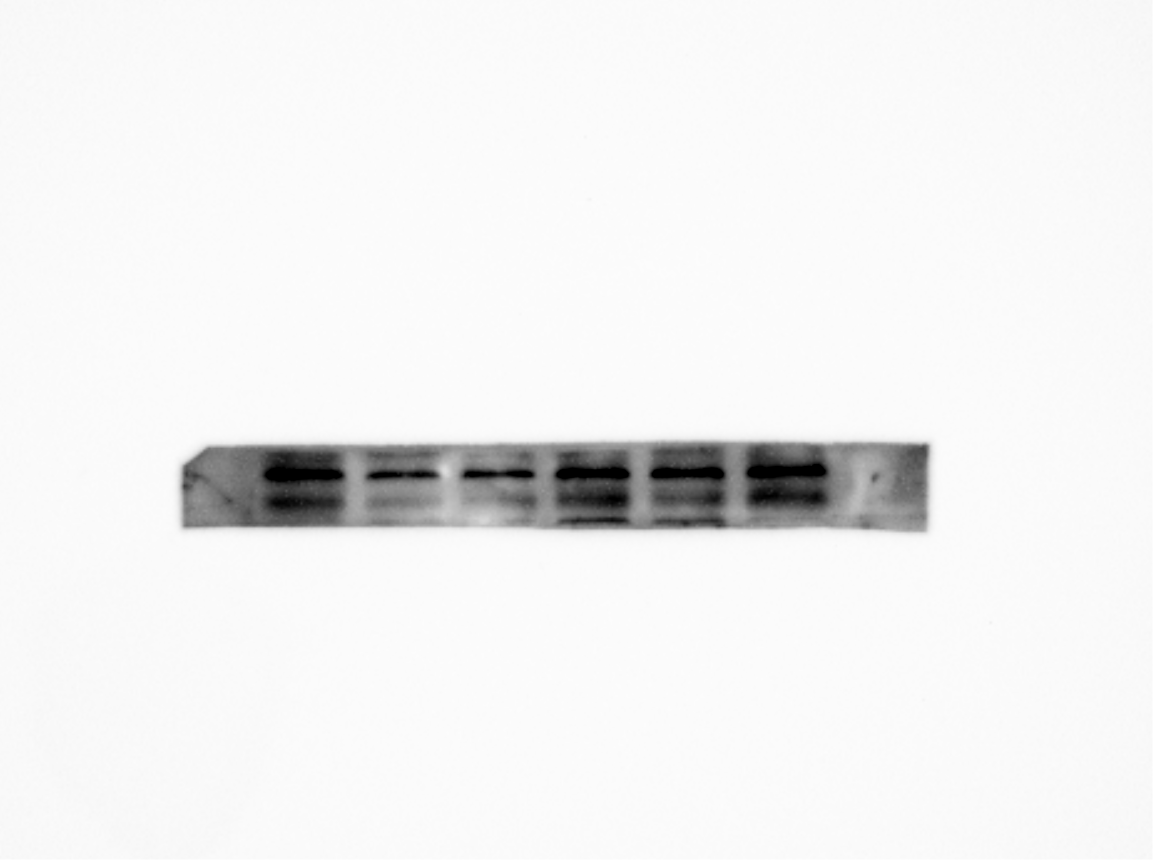


HMGCR
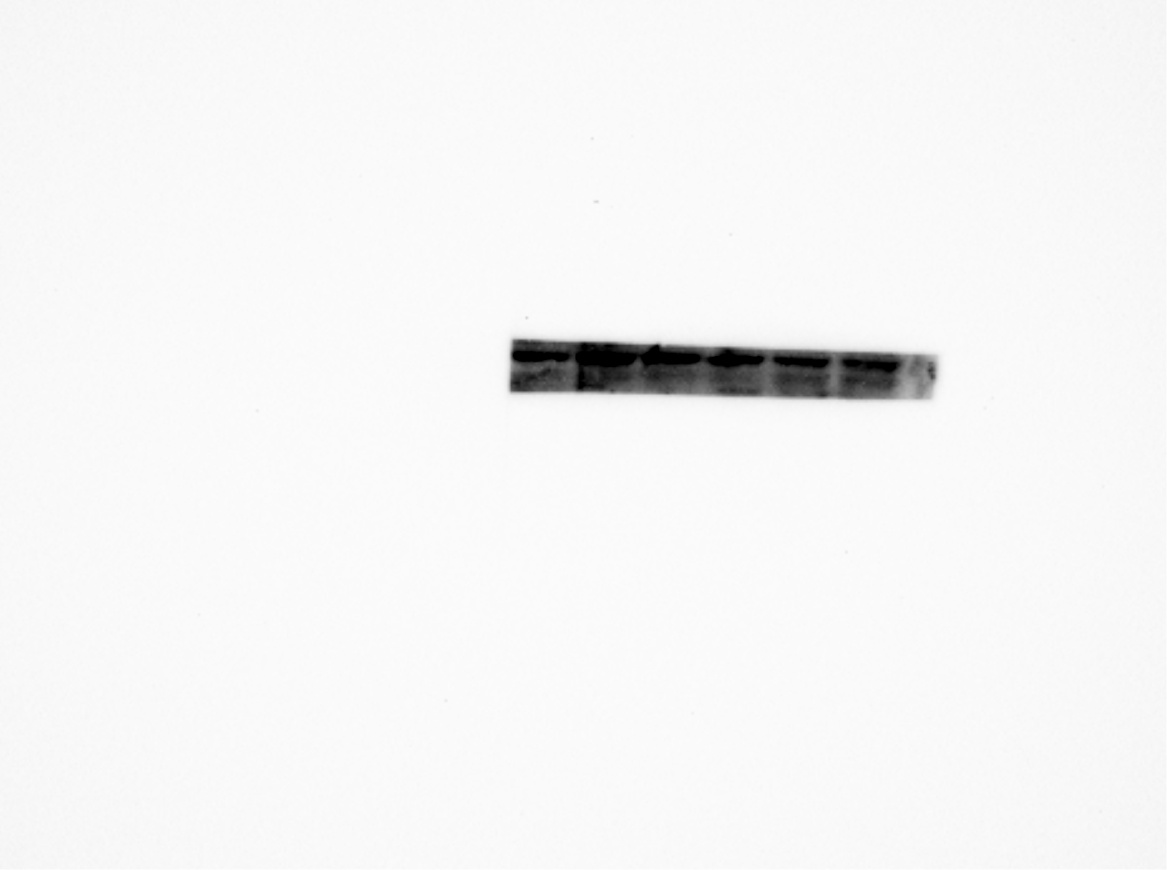


ABCG5
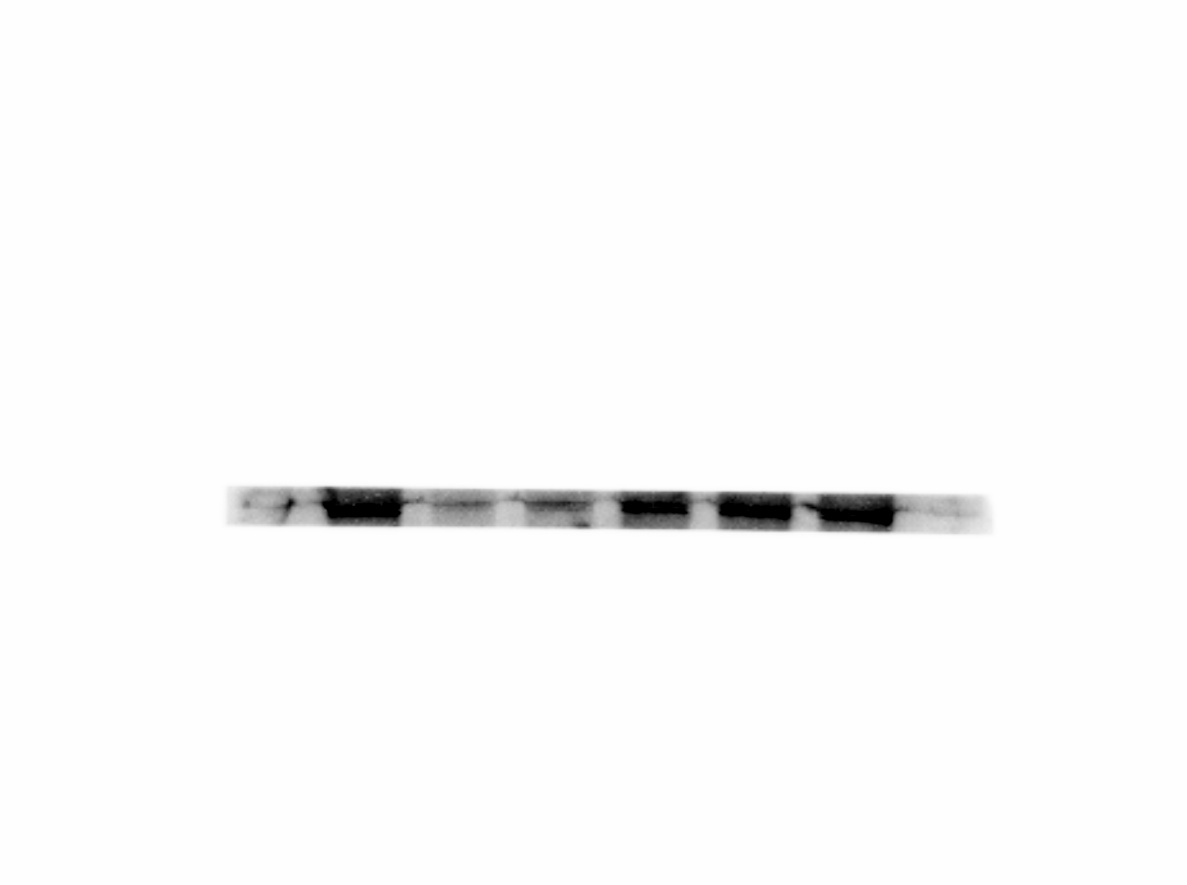


ABCG8
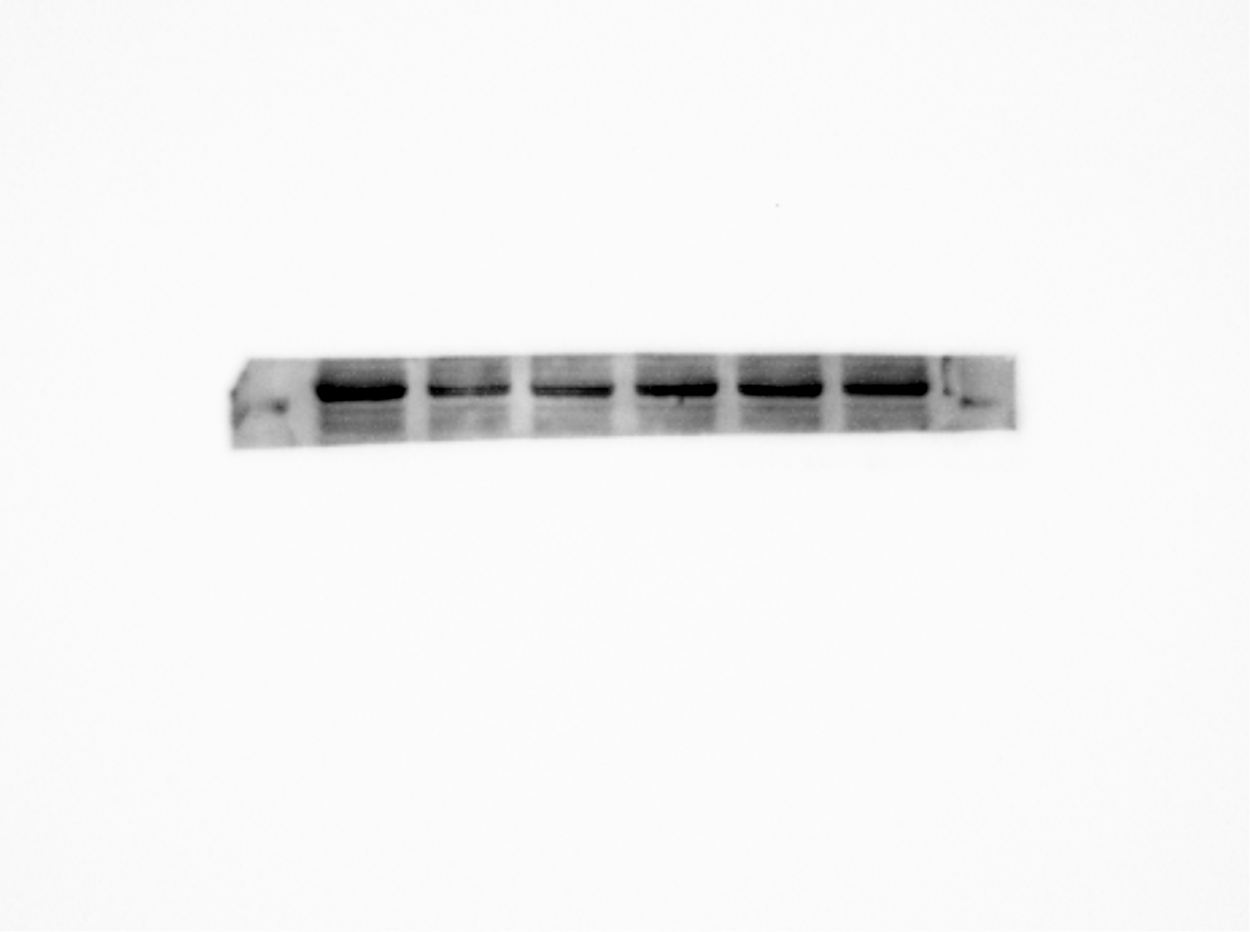


CYP7A1
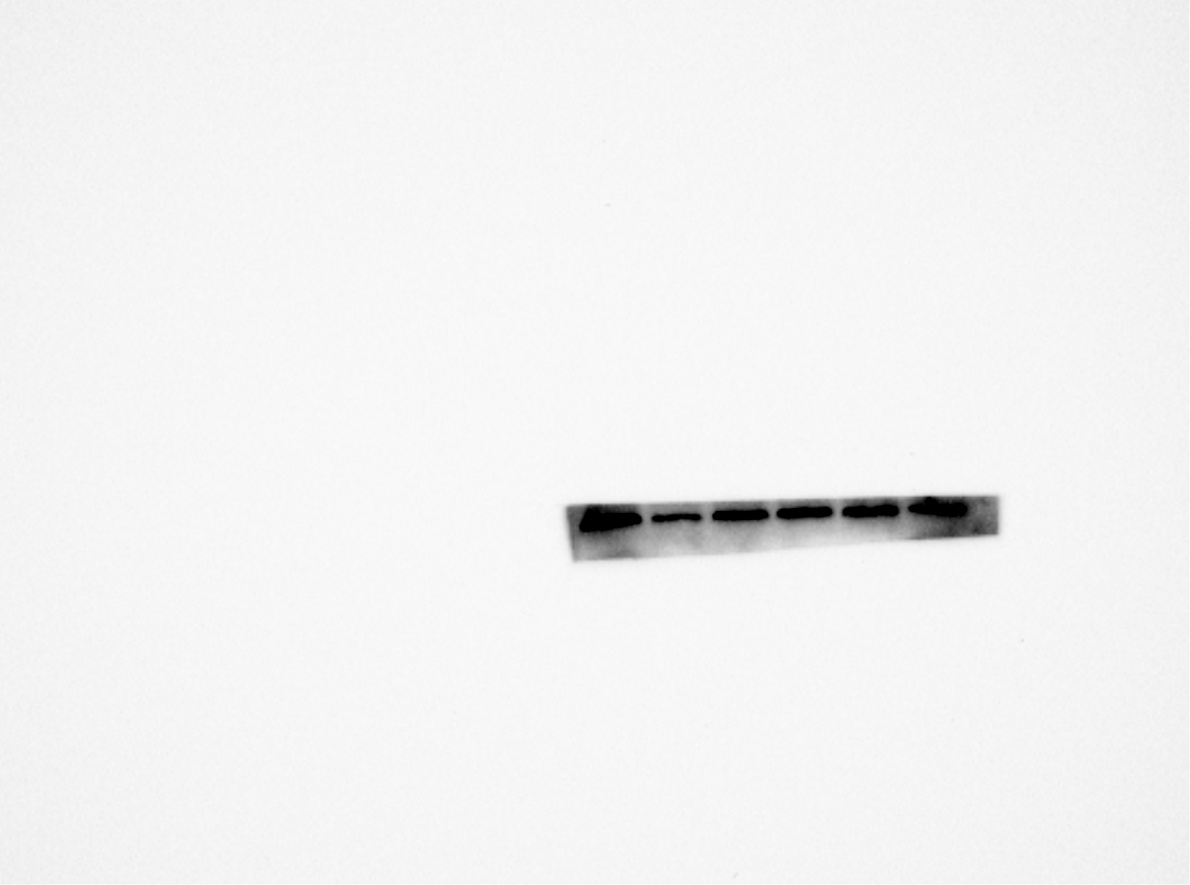


β-Actin
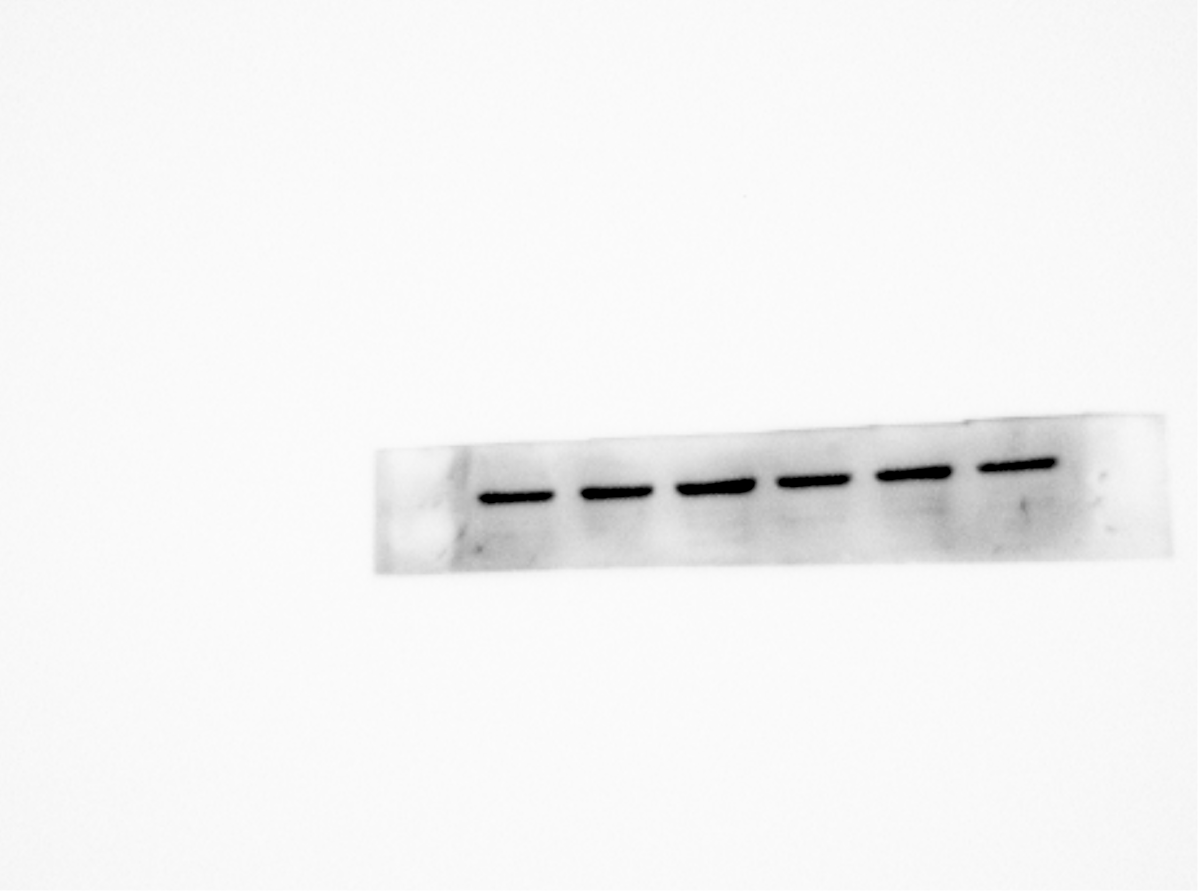


Fig. 5B


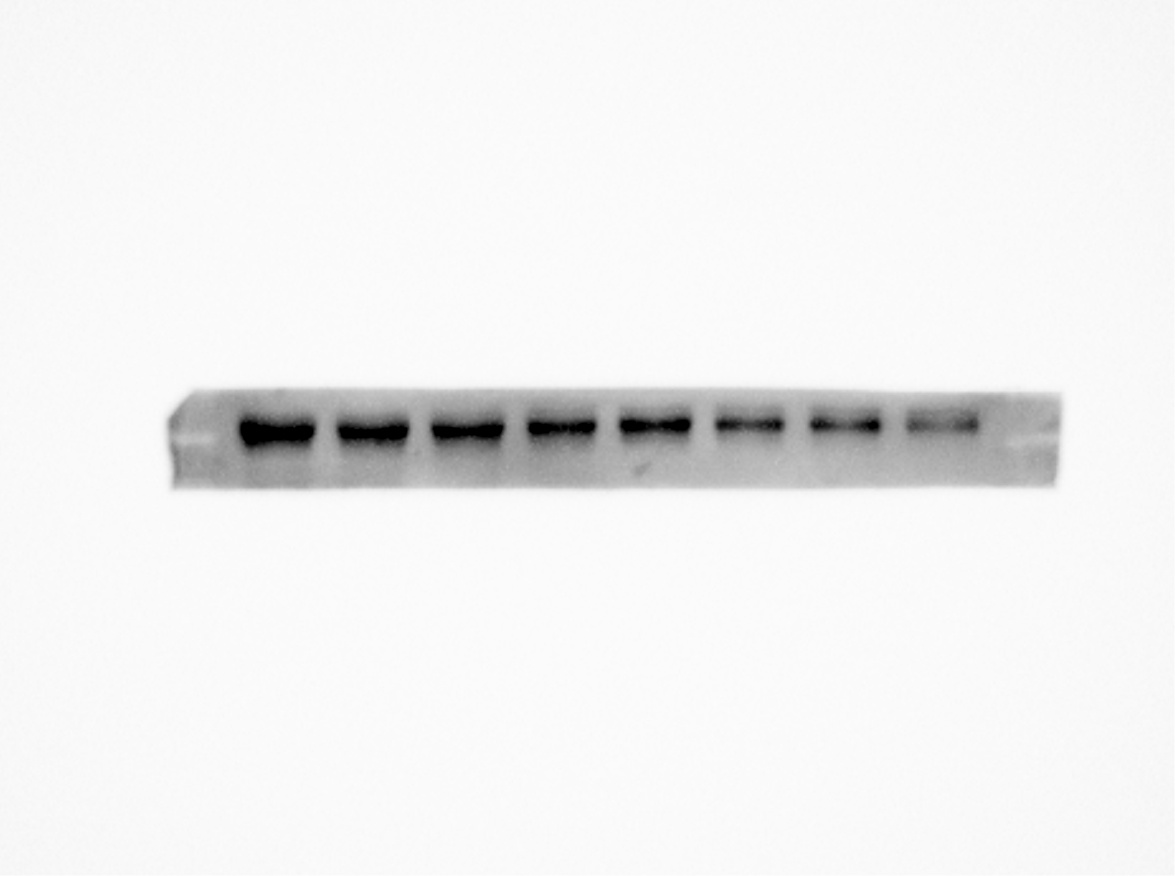
ASGR1 (control)

ASGR1 (Fisetin)
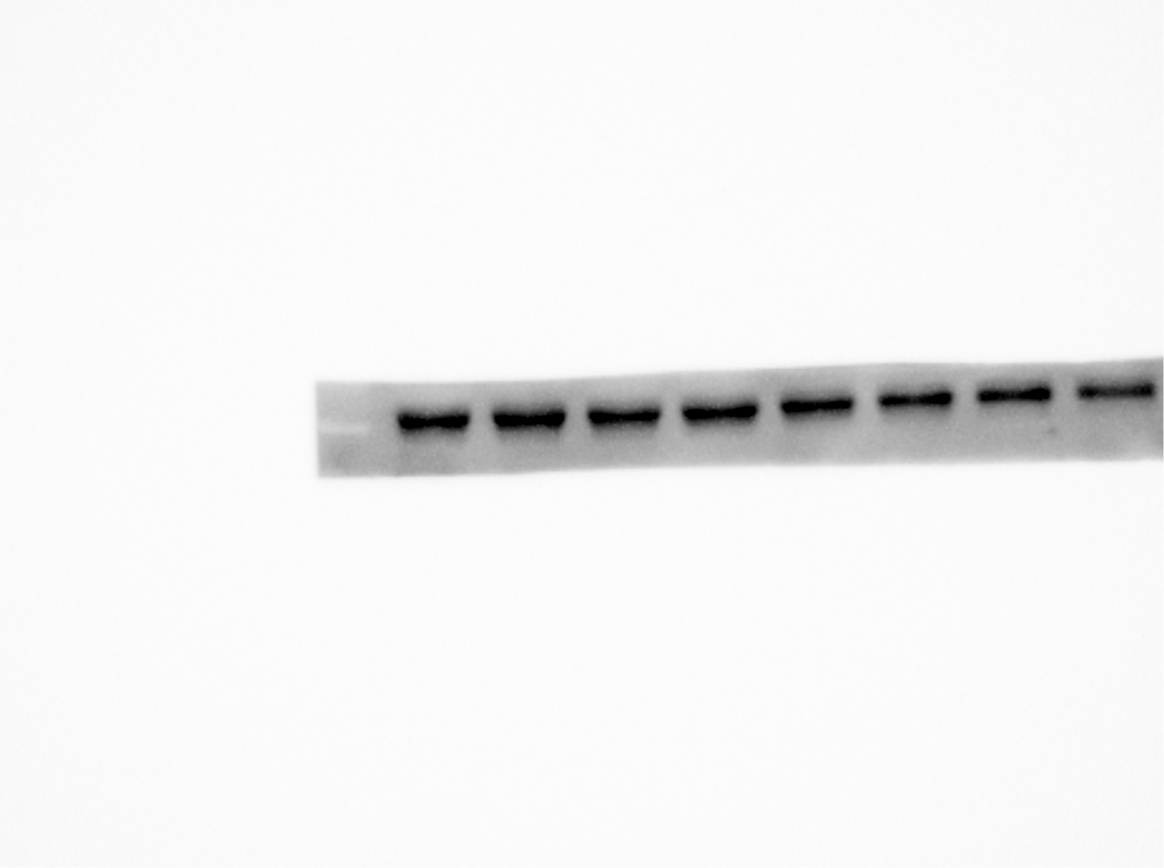


Fig. 5E

ASGR1
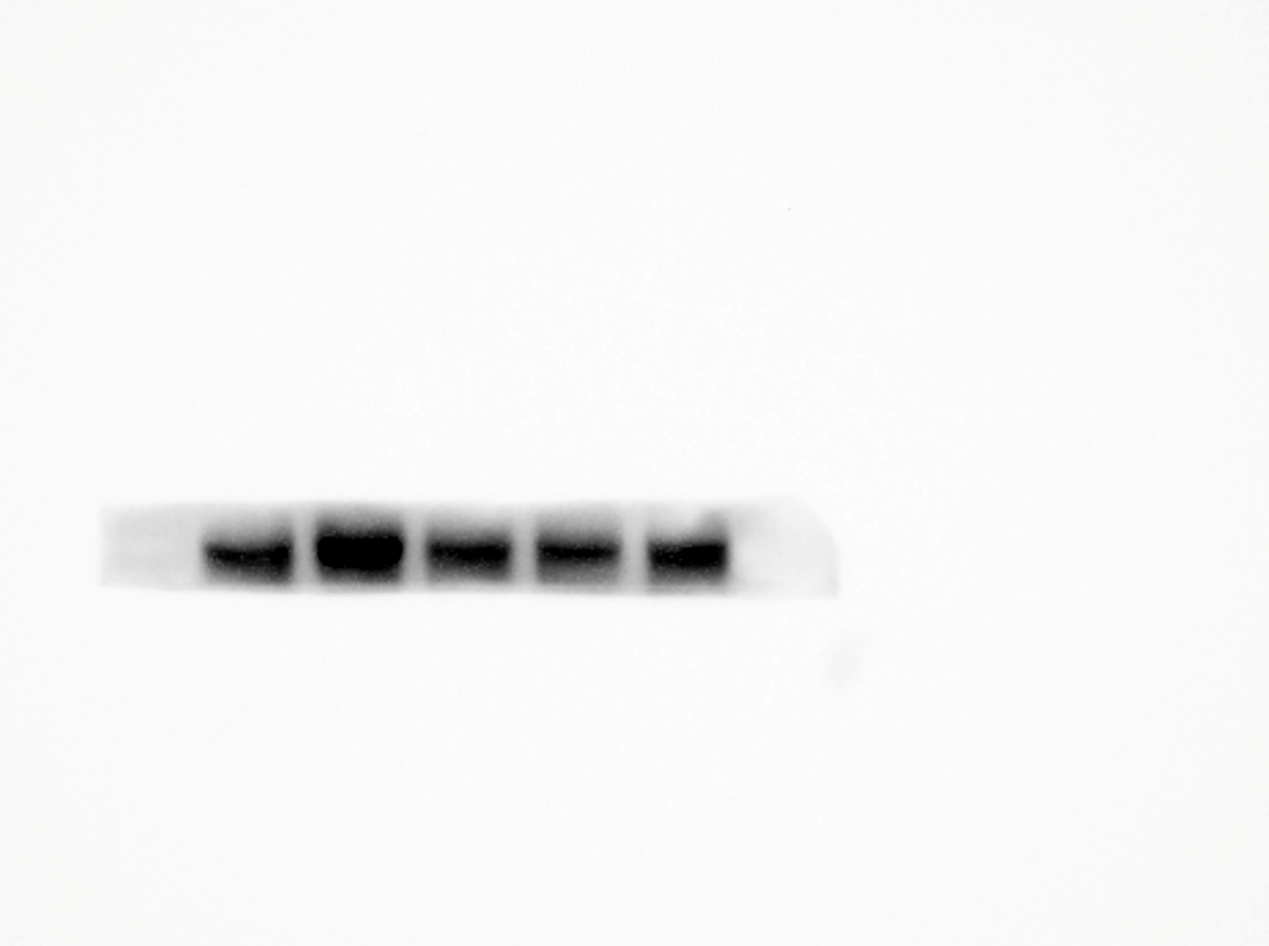


β-Actin
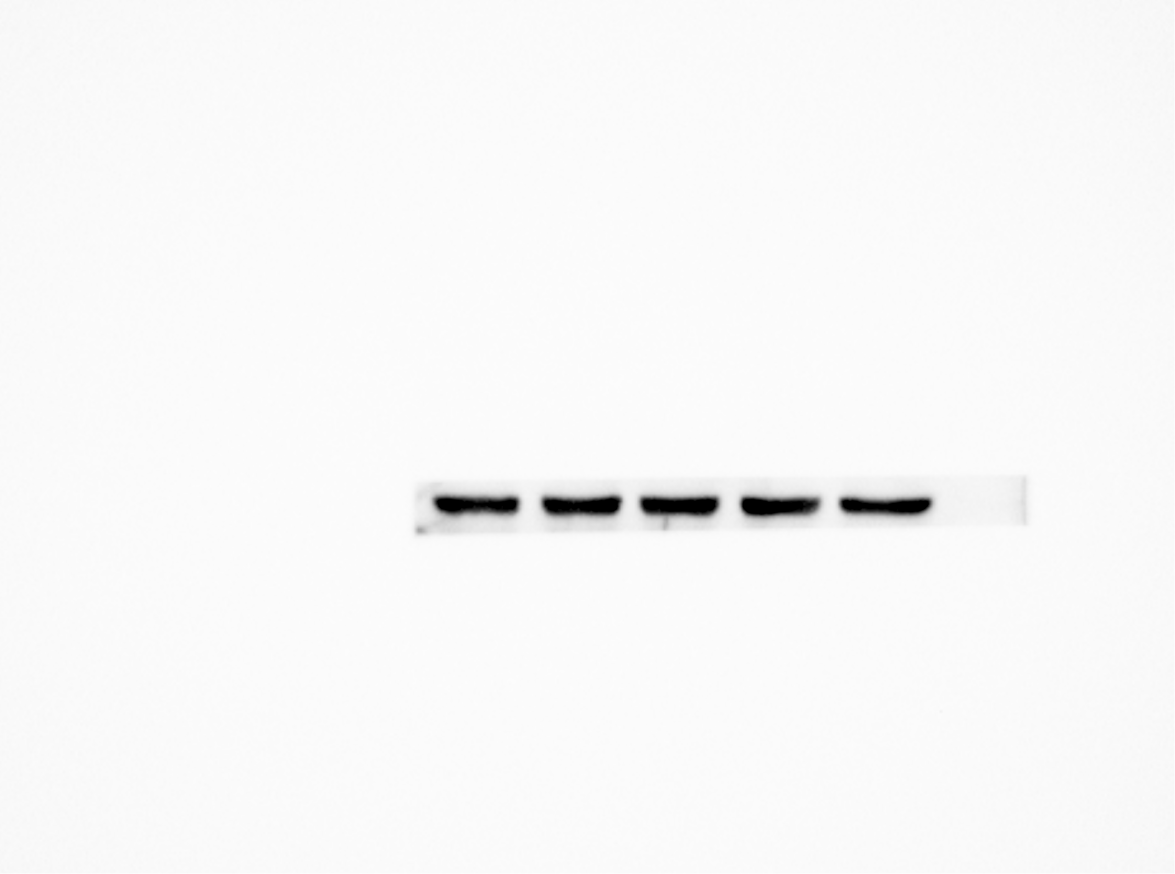


Fig. 5F

ASGR1
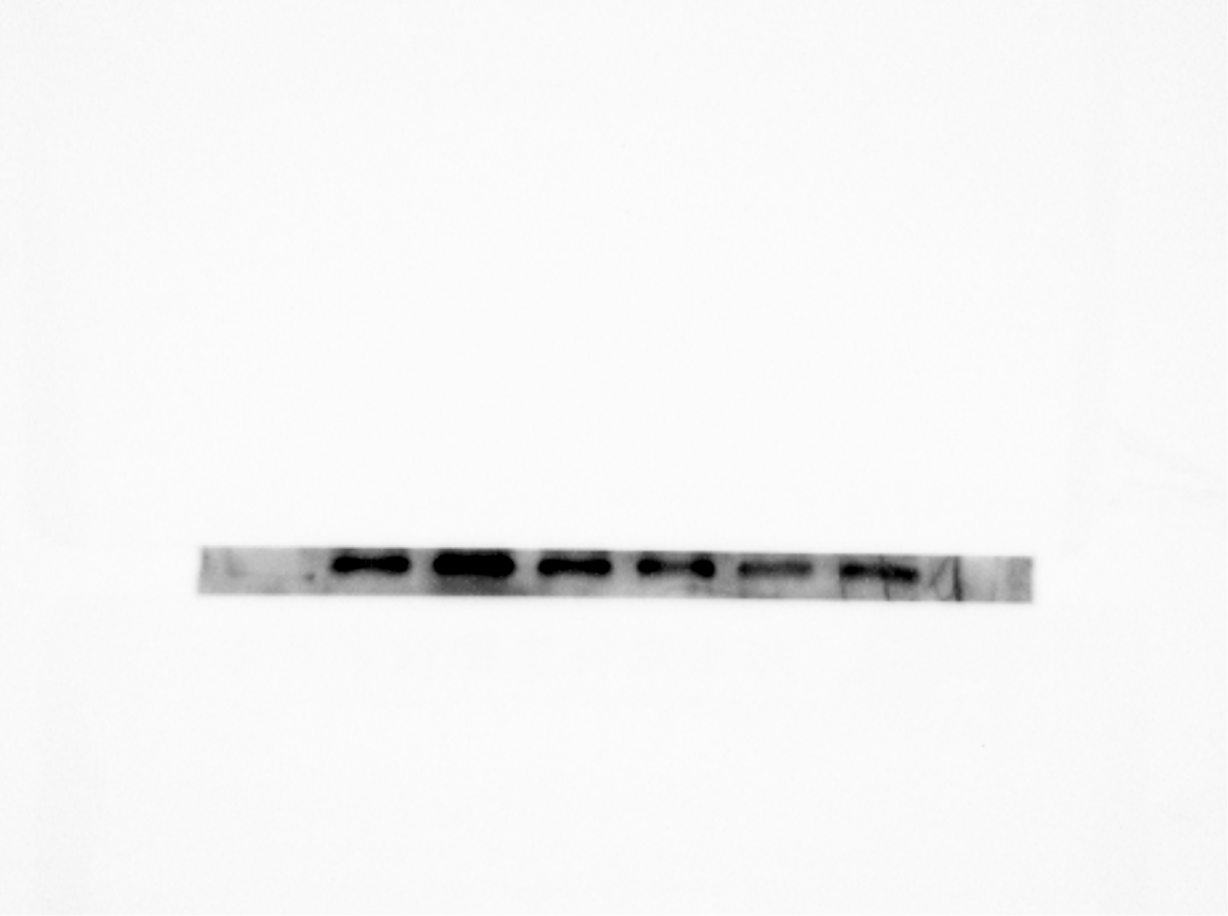


β-Actin
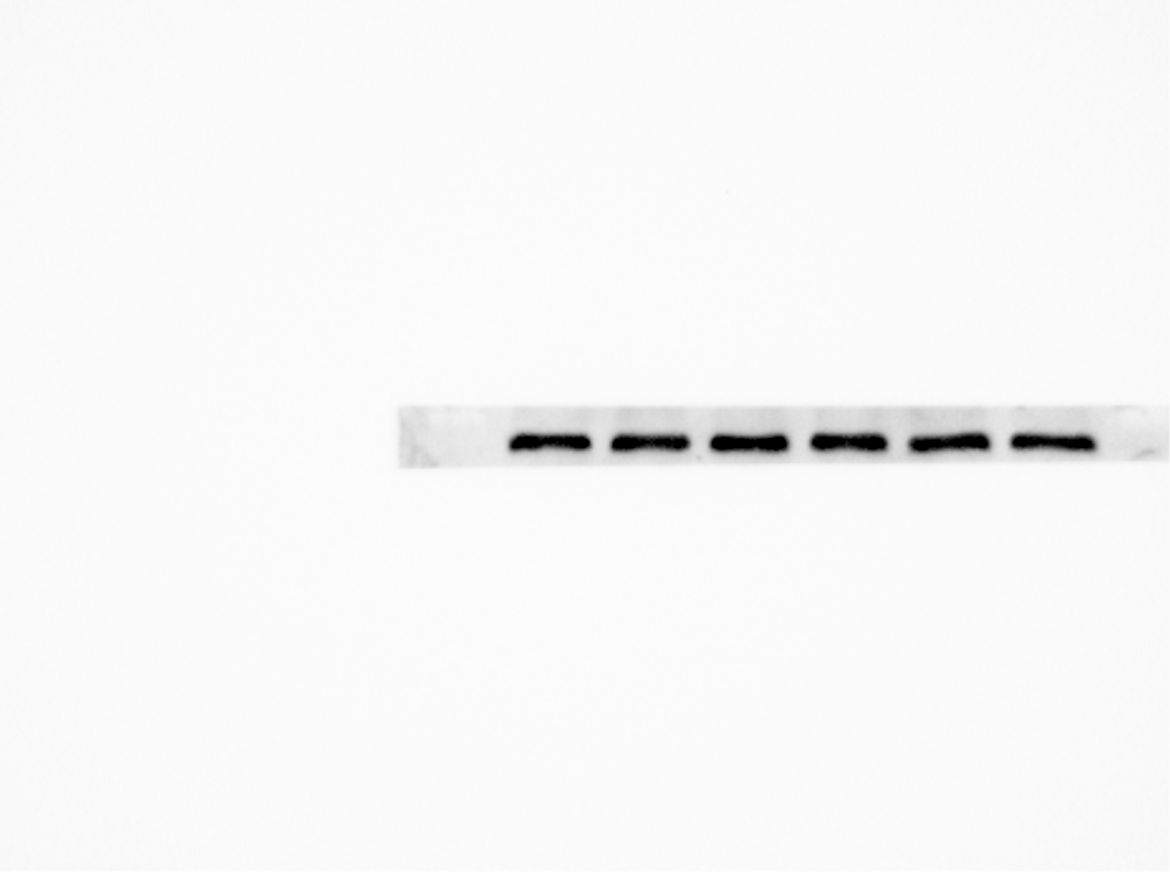


Fig. 6B

p-S6K
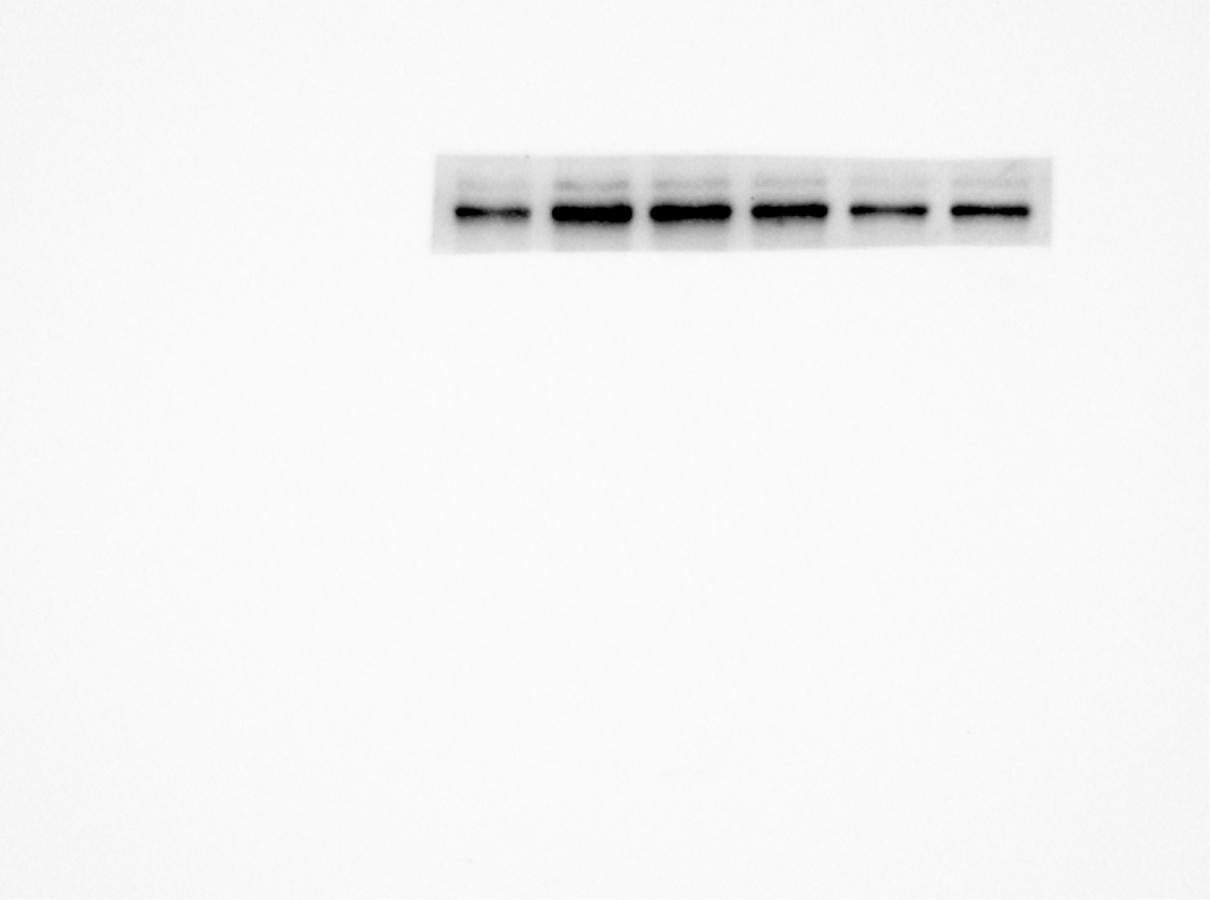


S6K
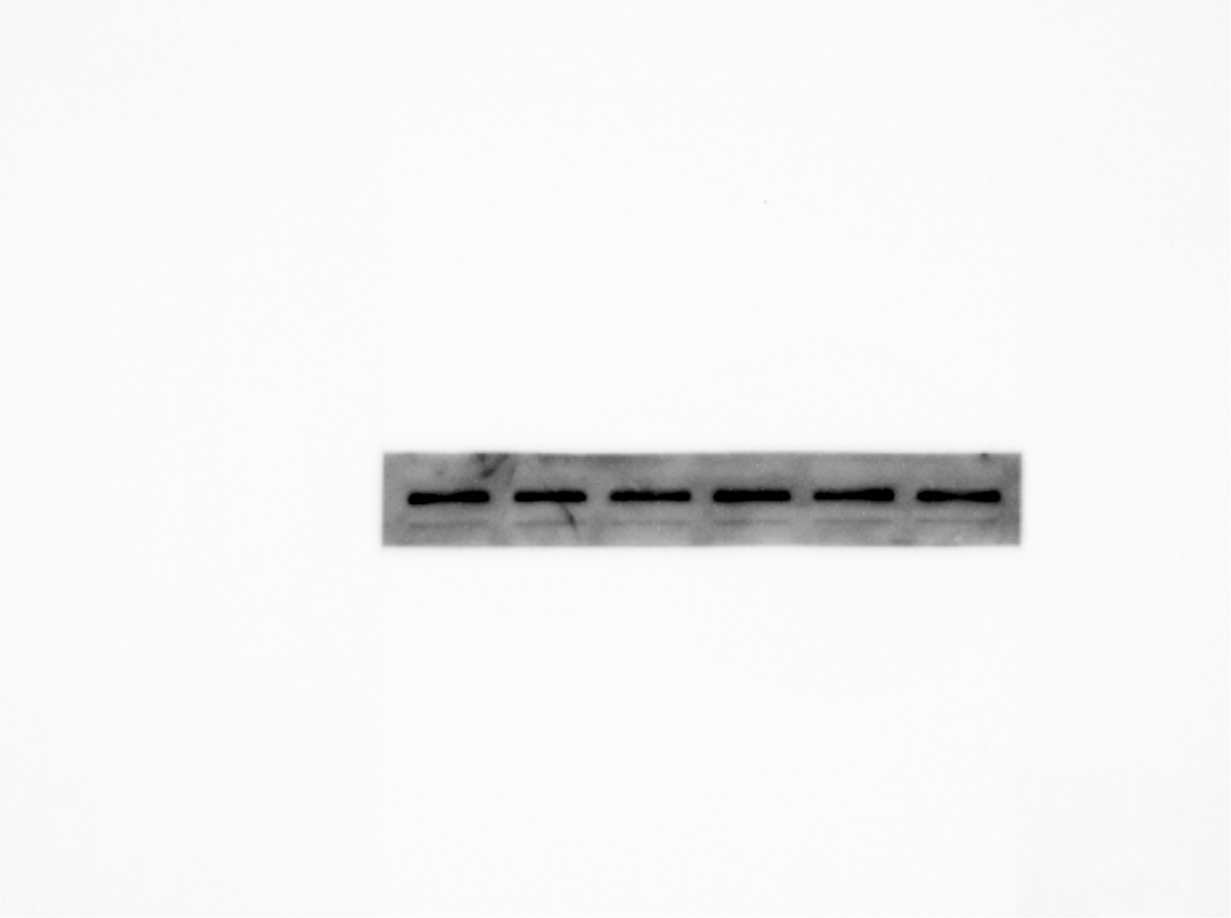


p-mTOR
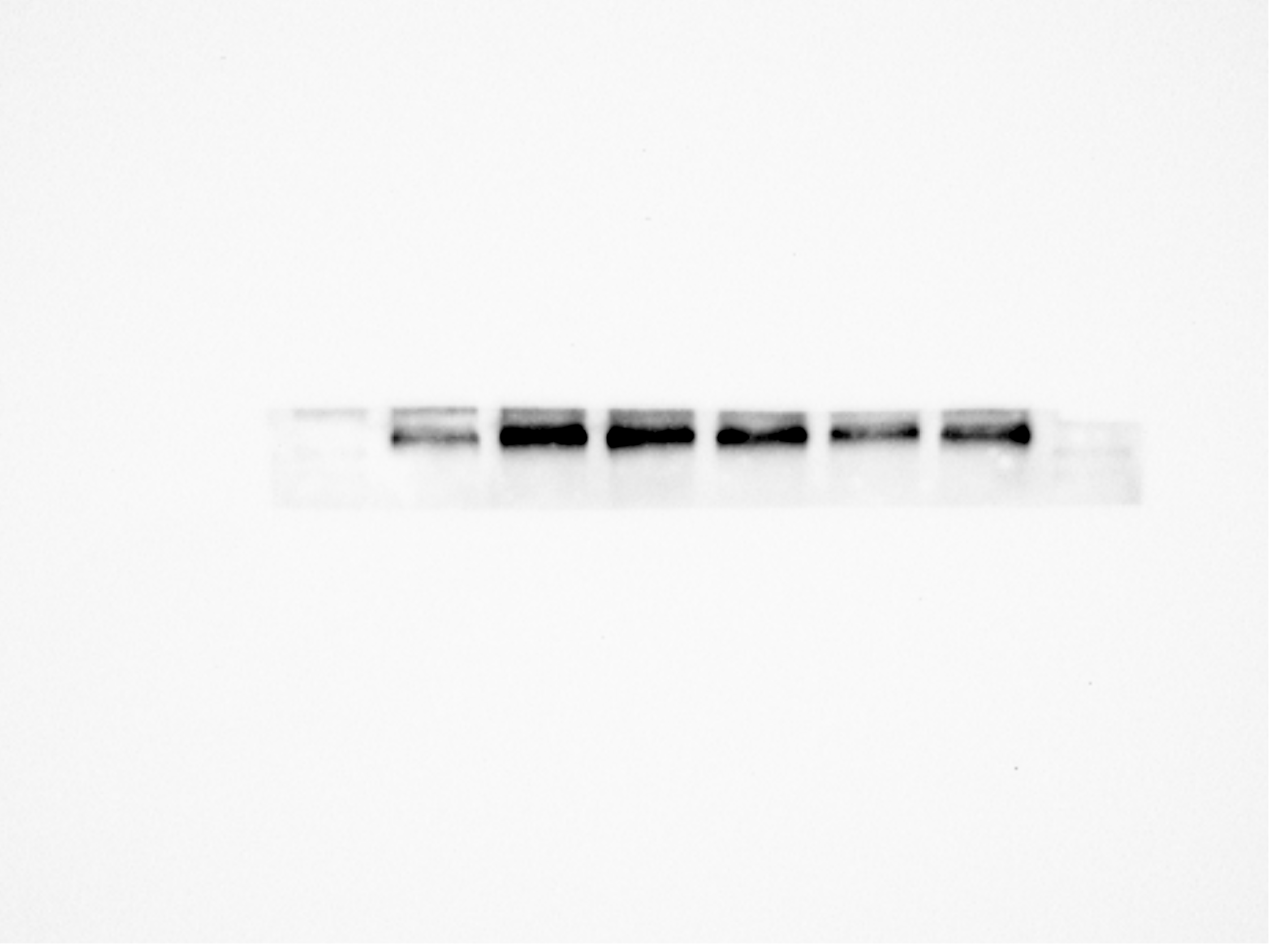


mTOR
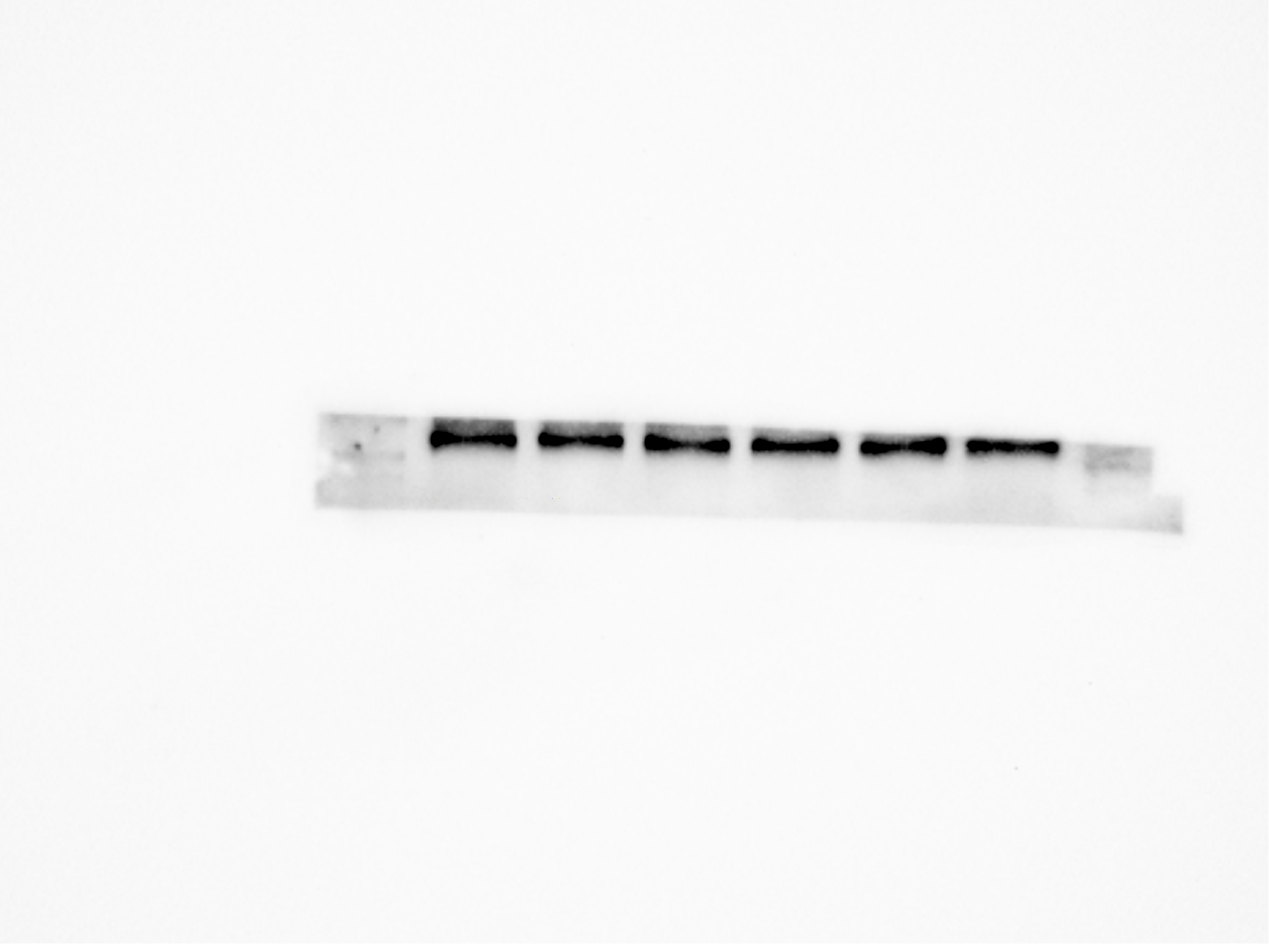


p-AMPK


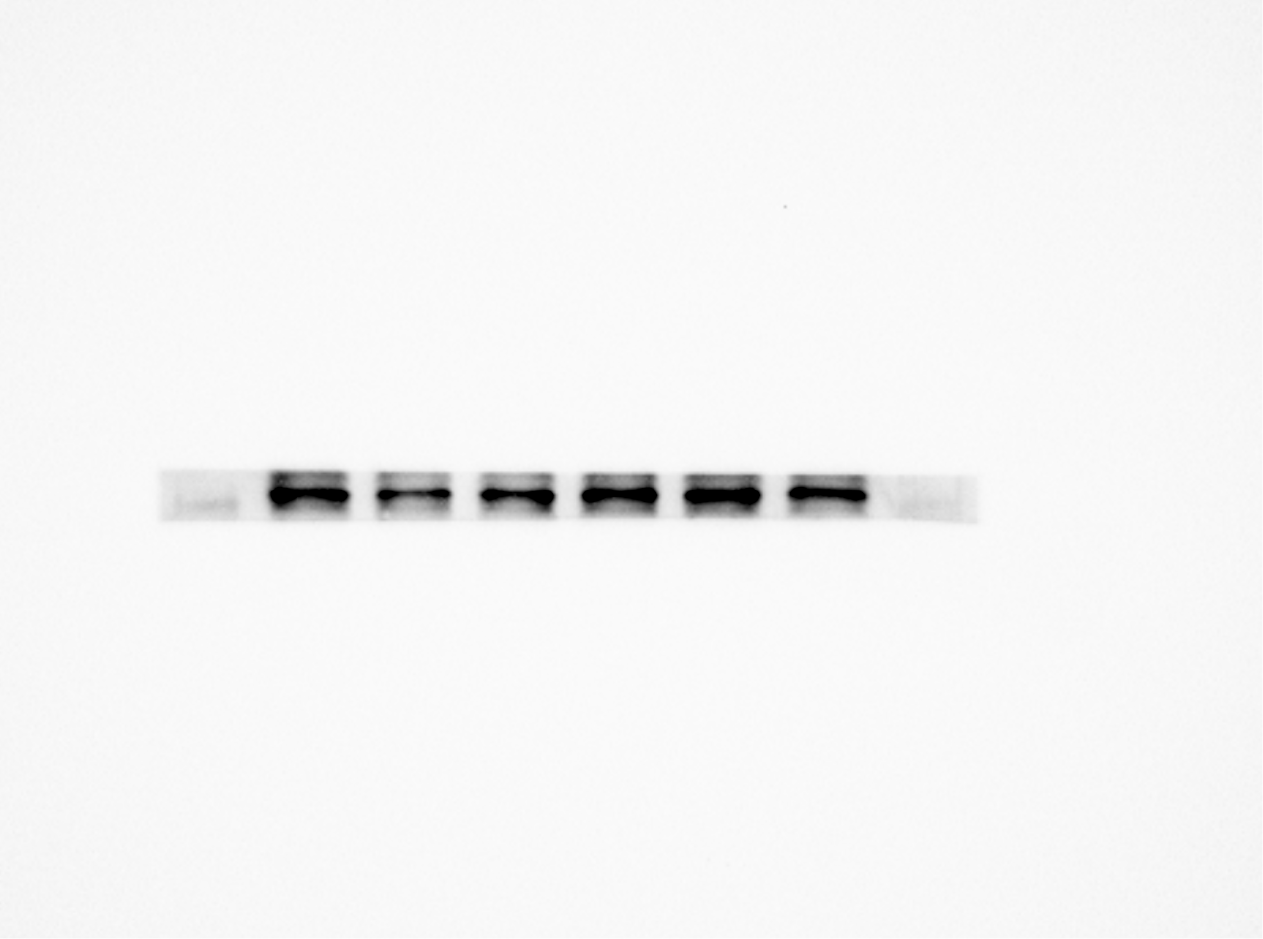


AMPK


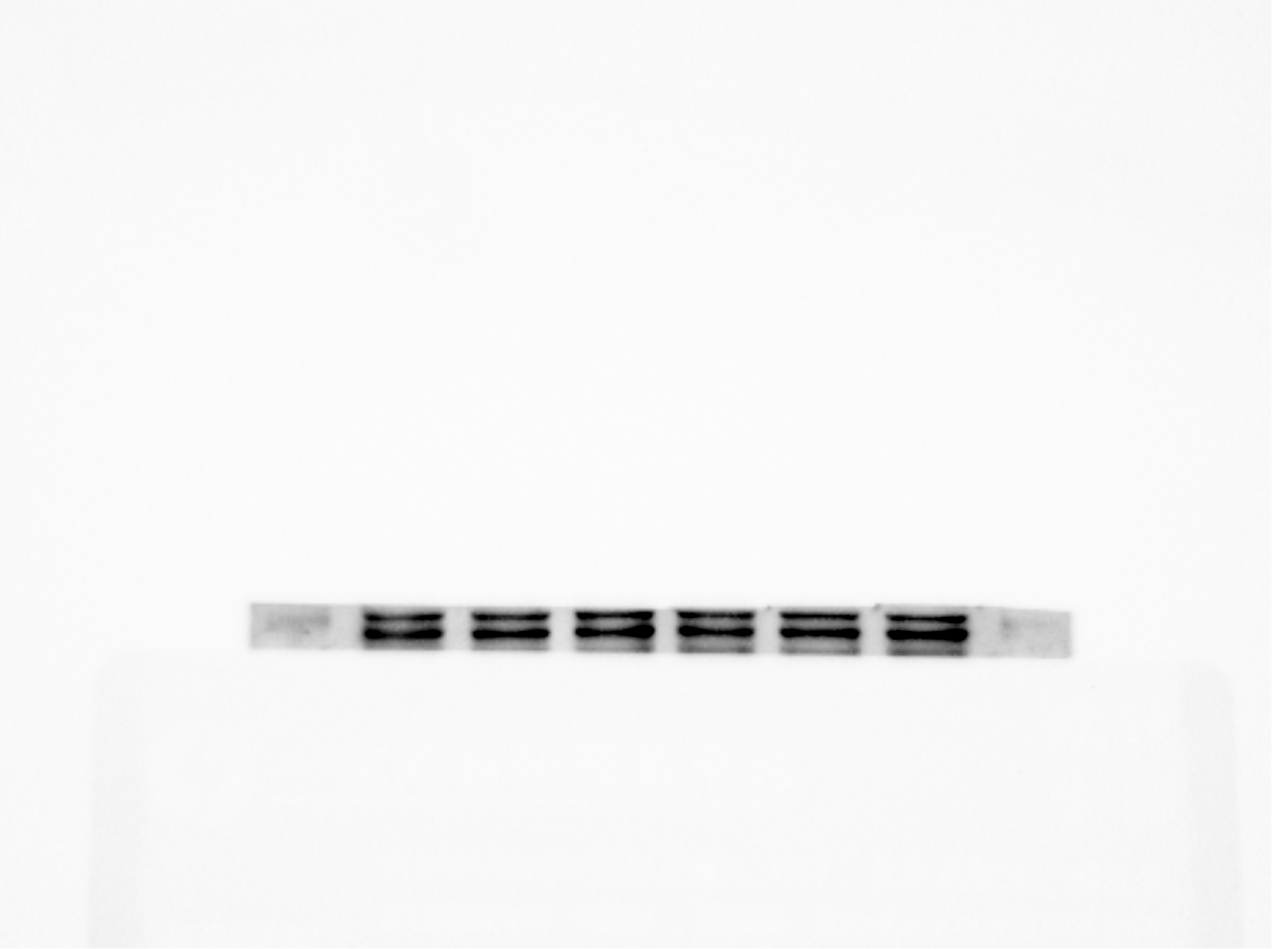


BRCA1


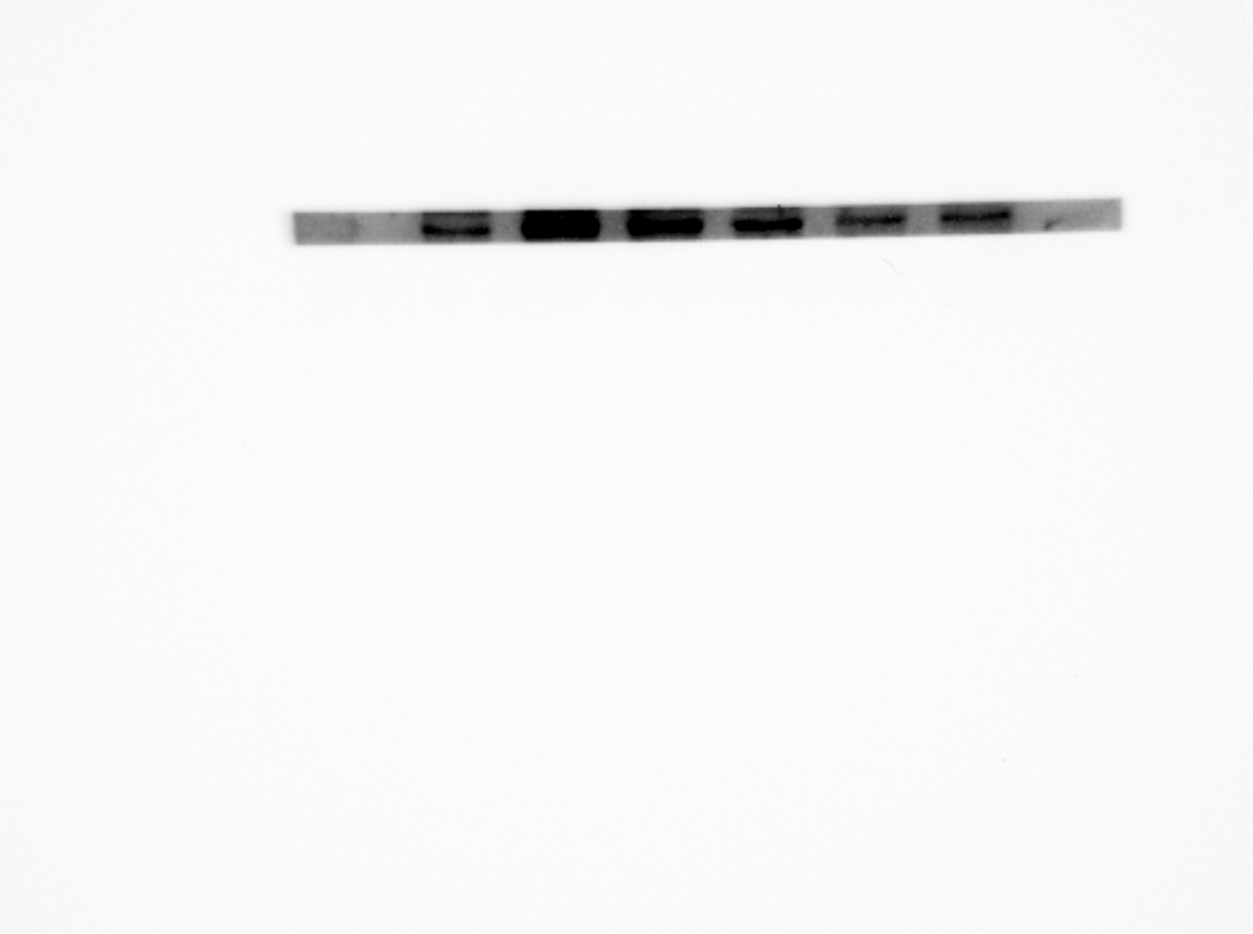


BARD1


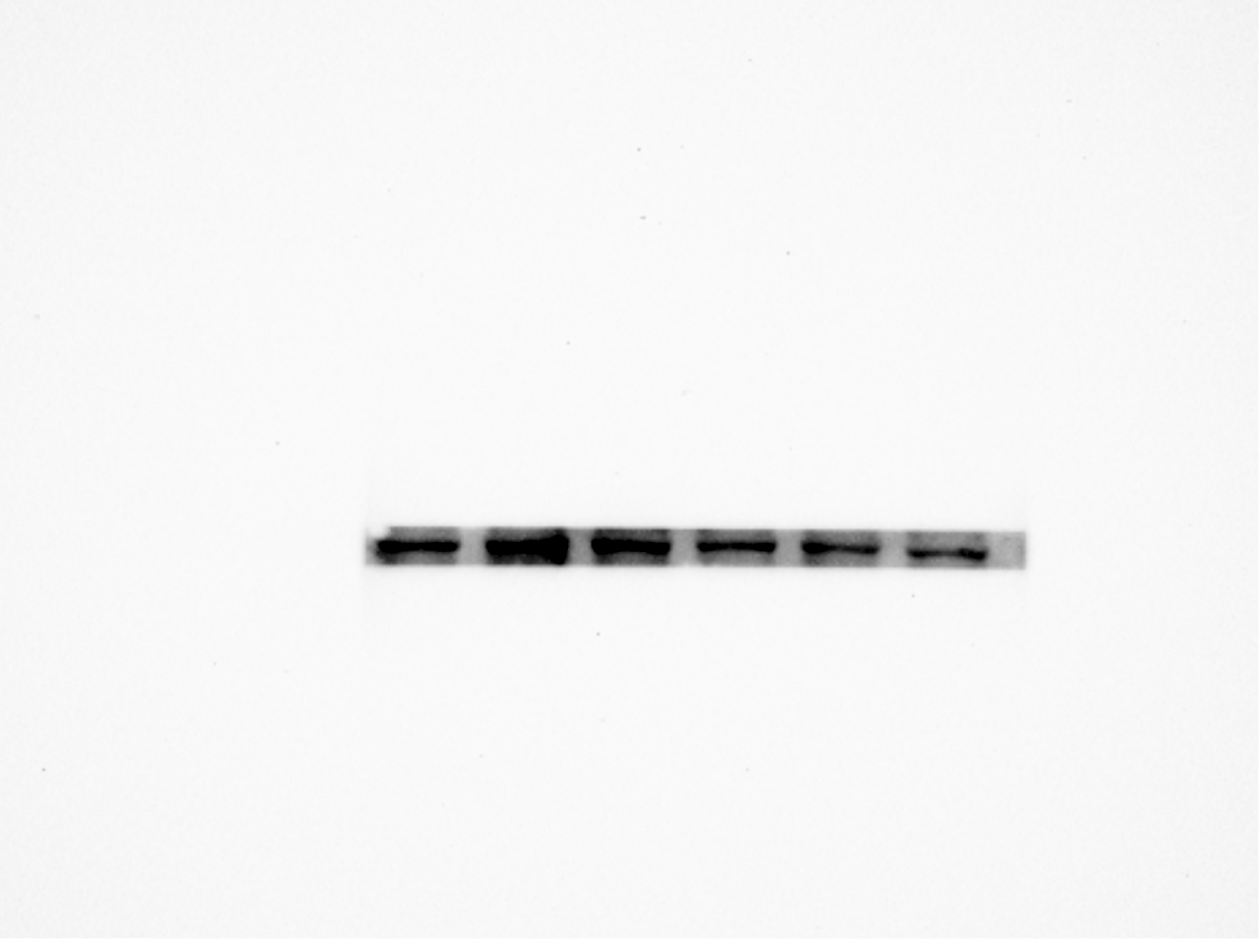


β-Actin


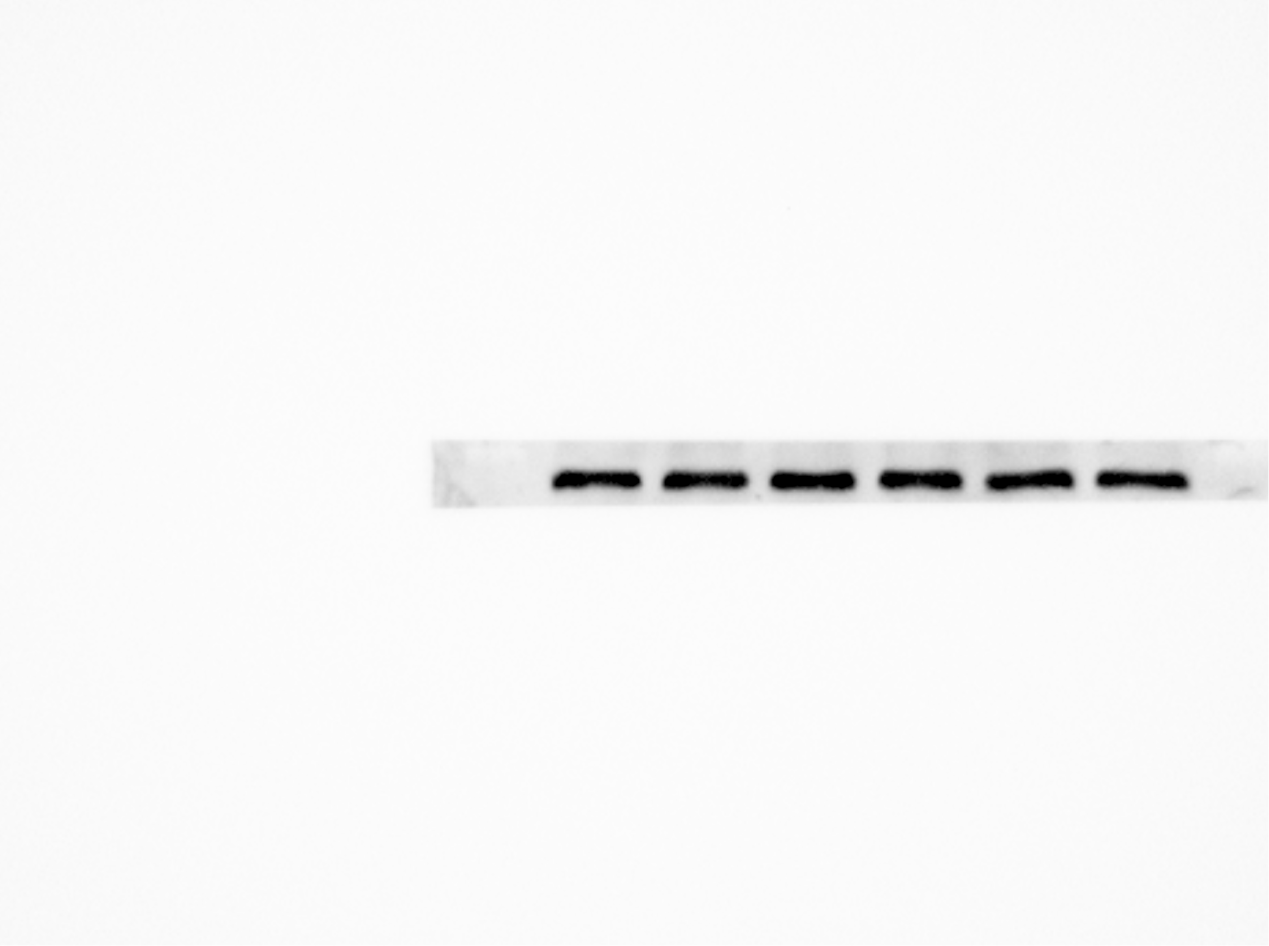


Fig.7A

p-S6K


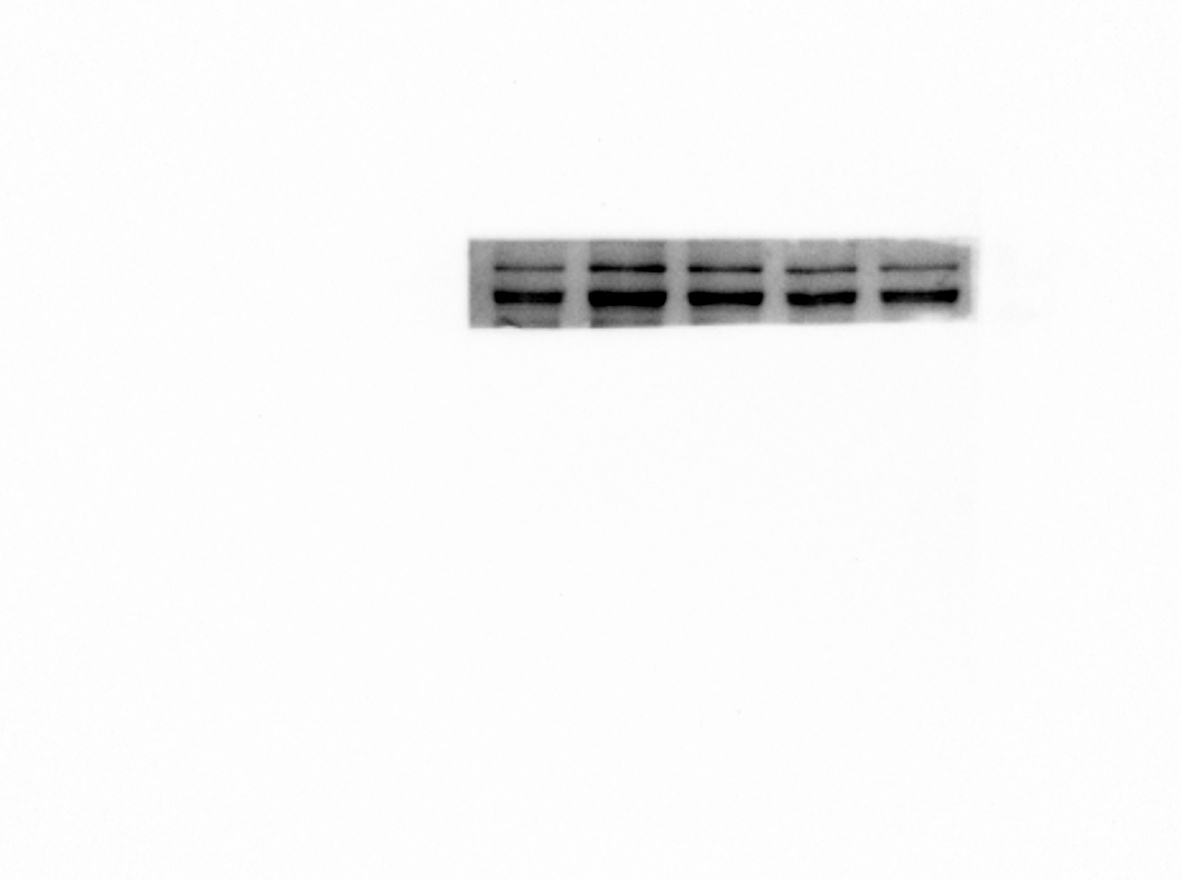


S6K


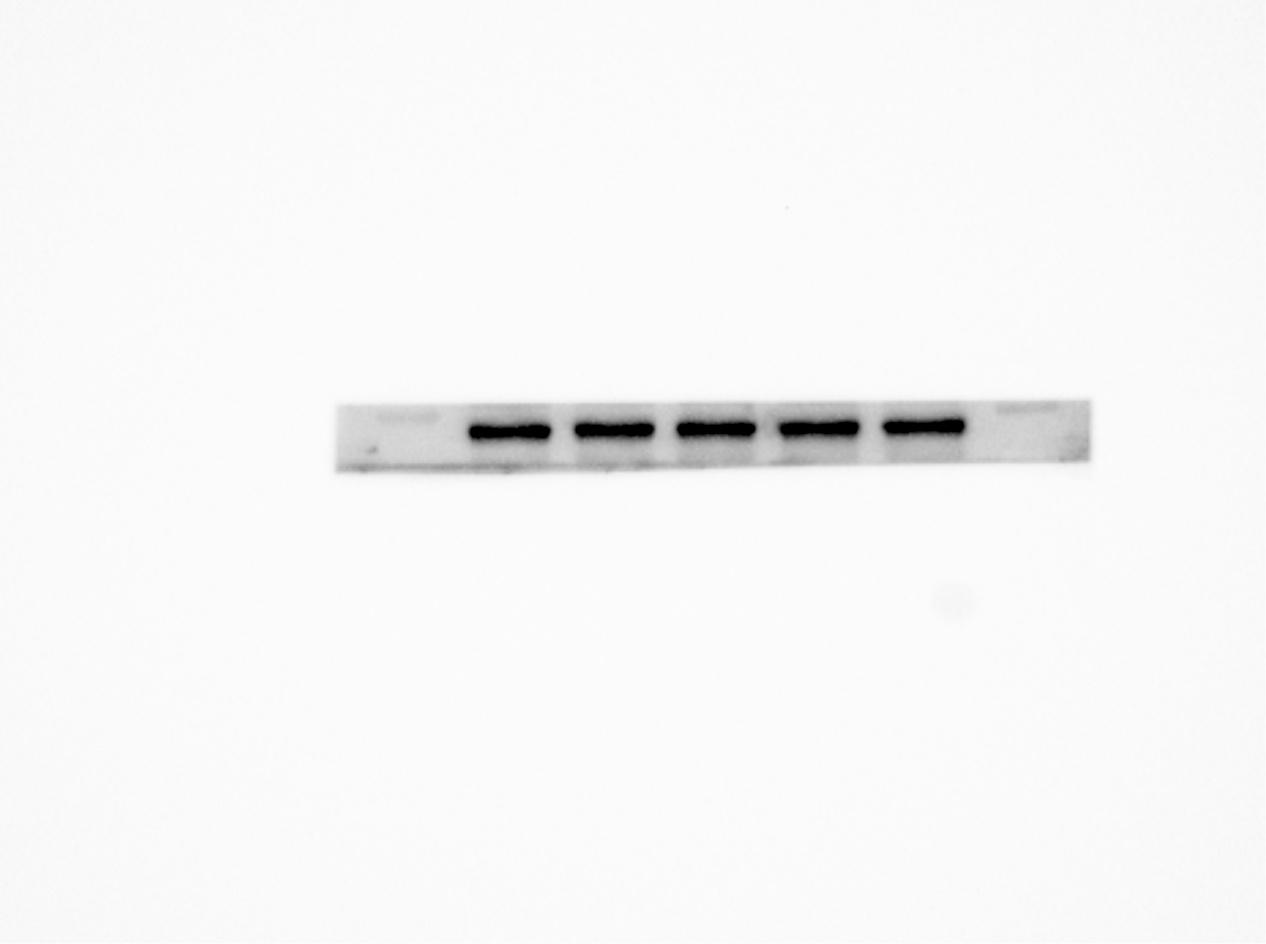


p-mTOR


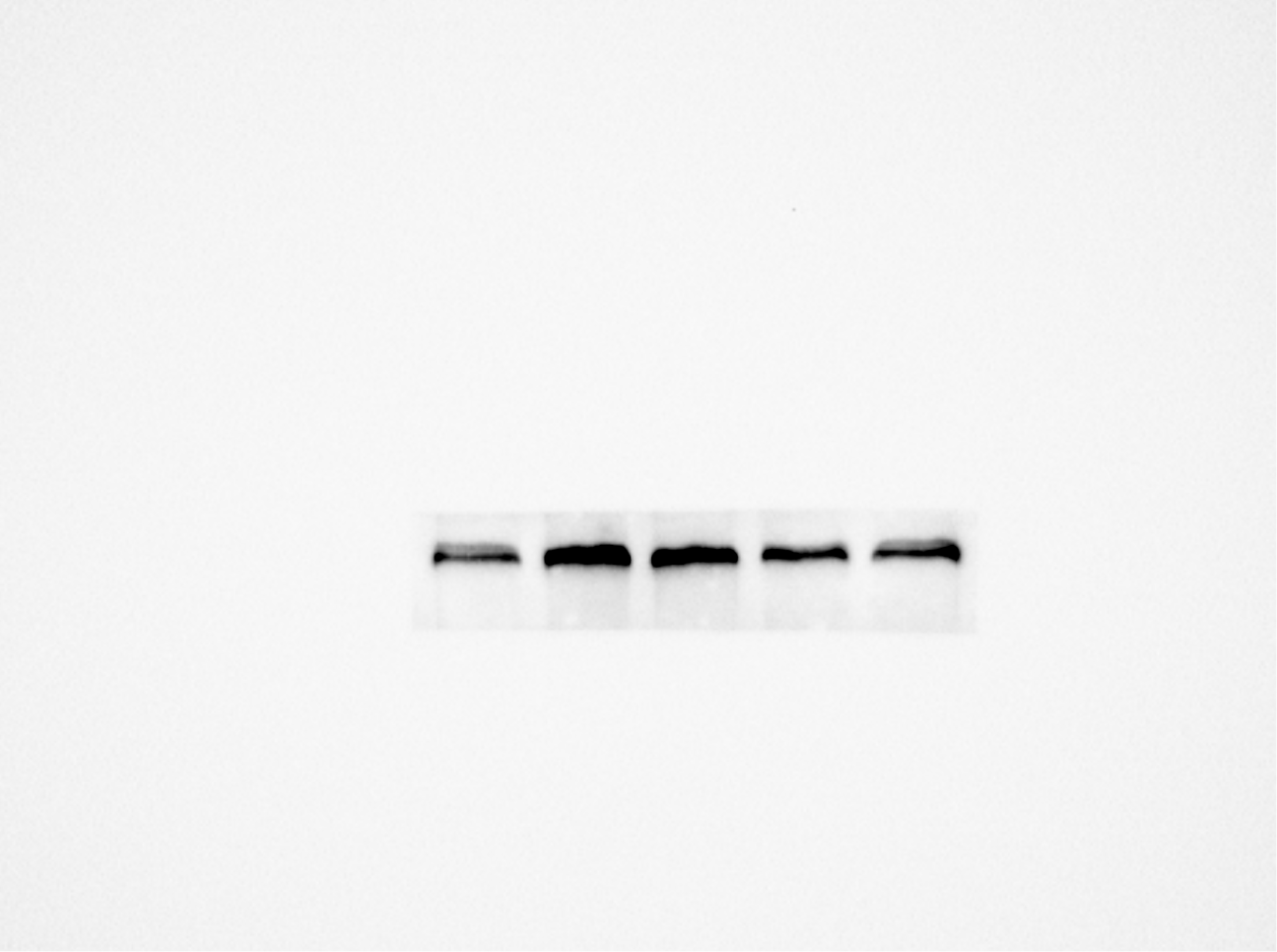


mTOR


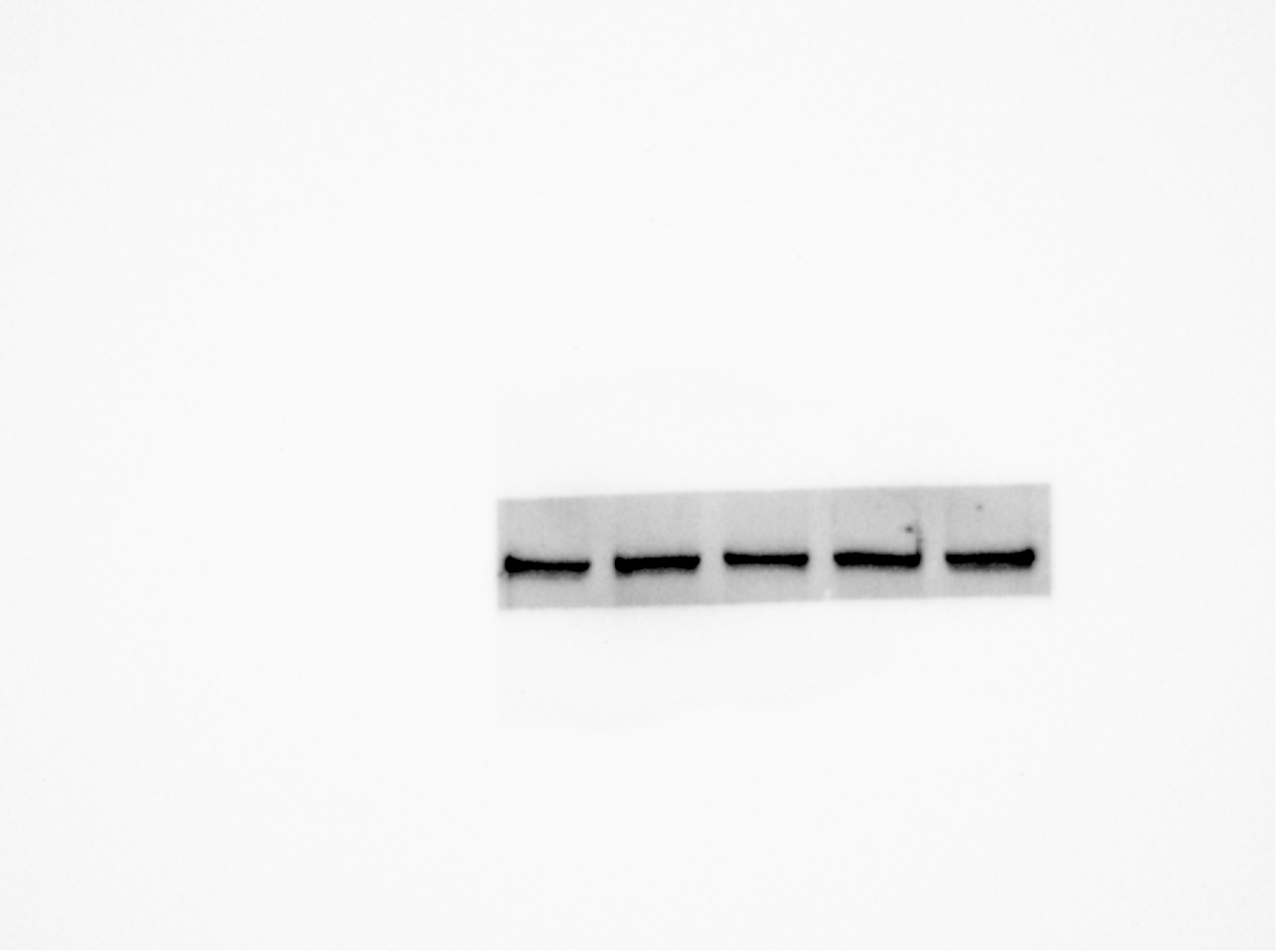


p-AMPK


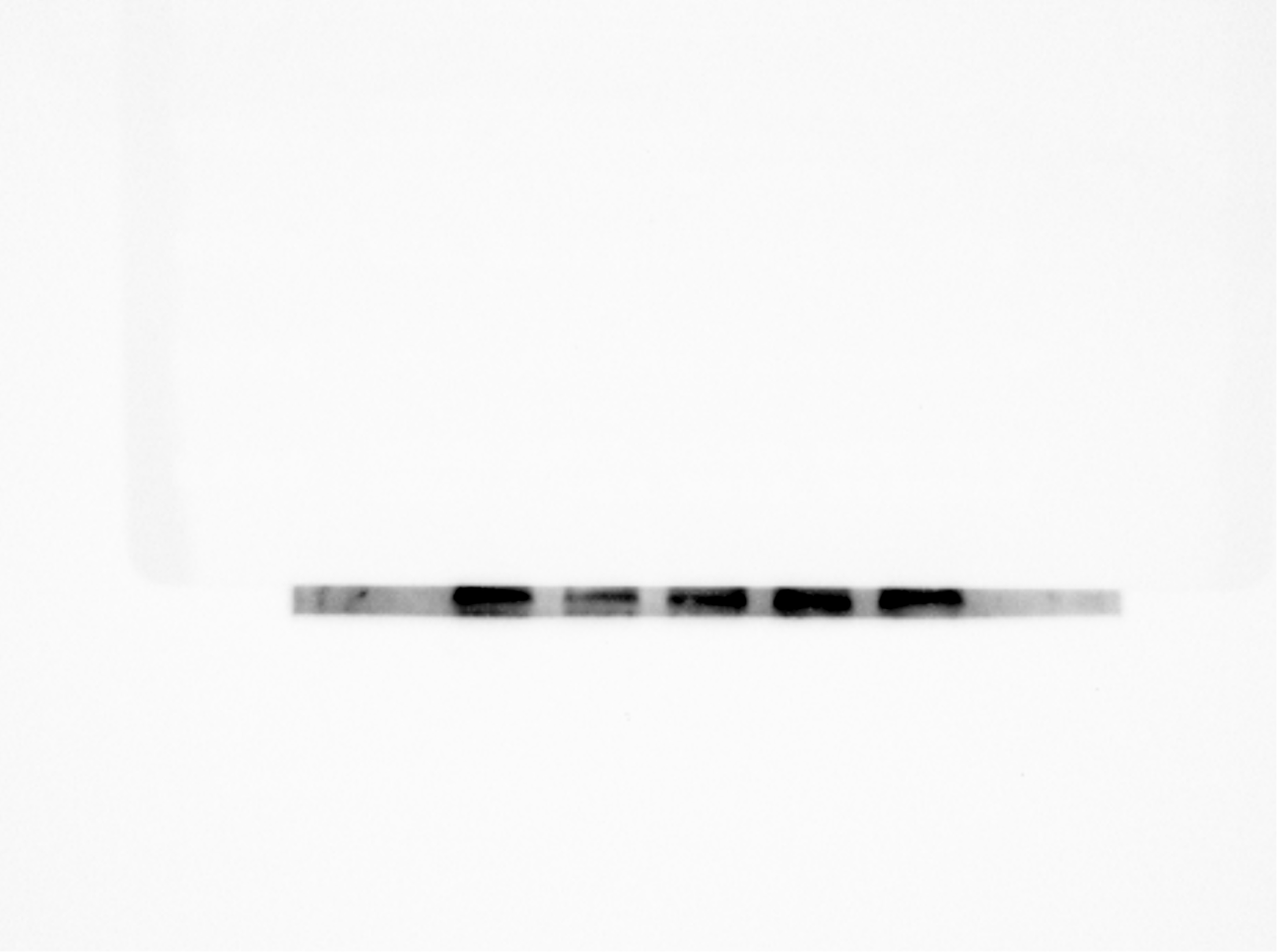


AMPK


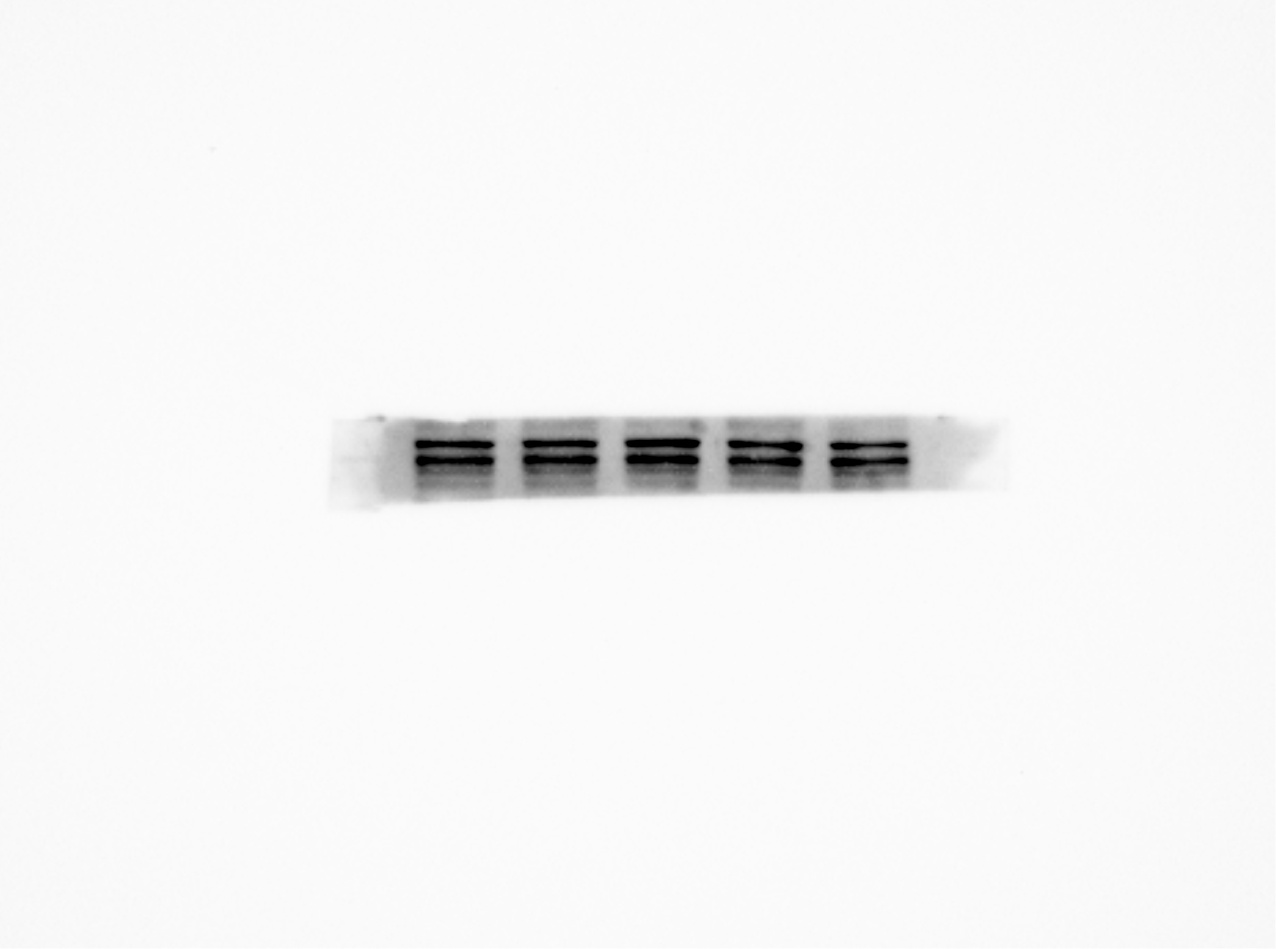


BRCA1


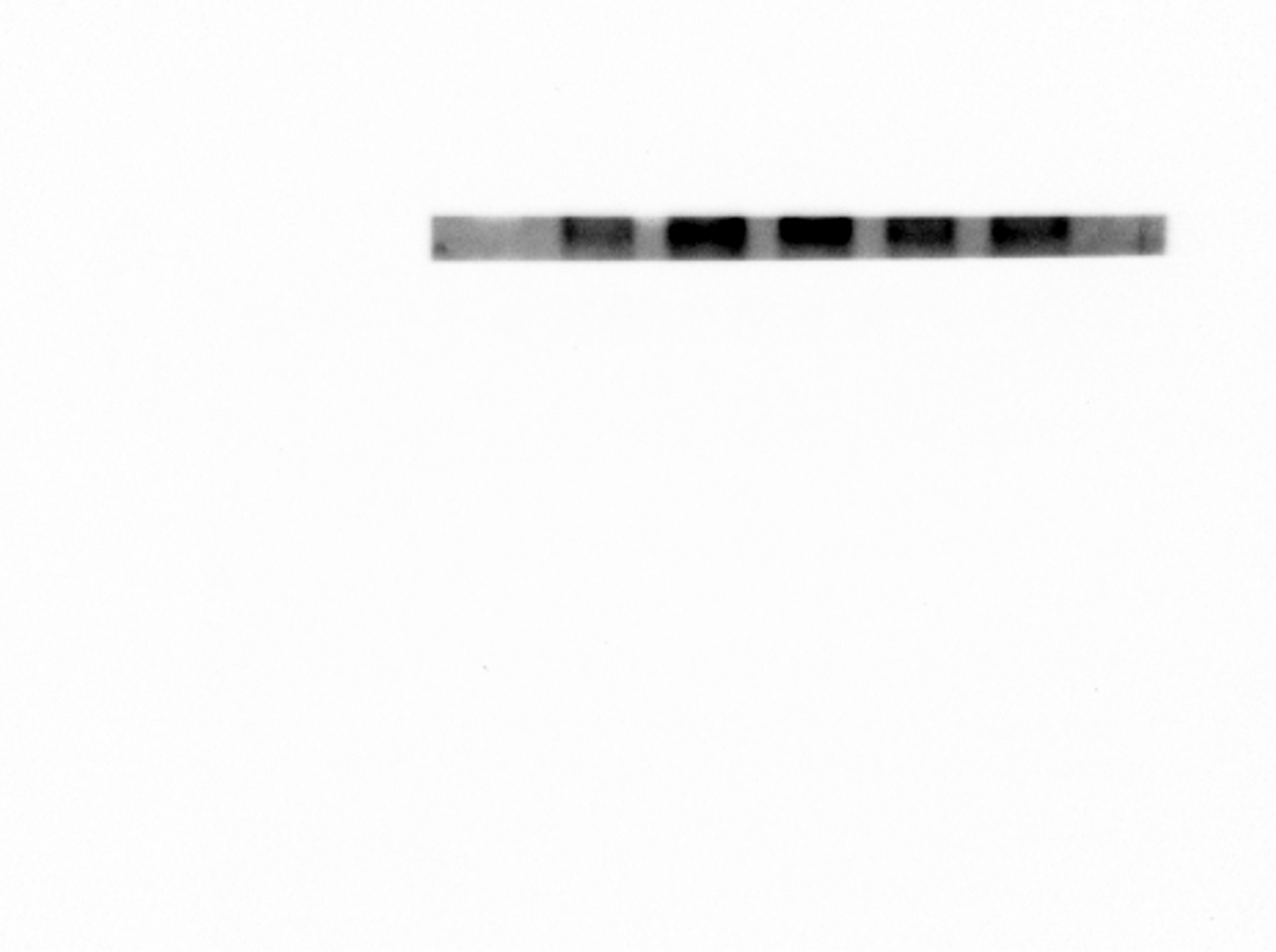


BARD1


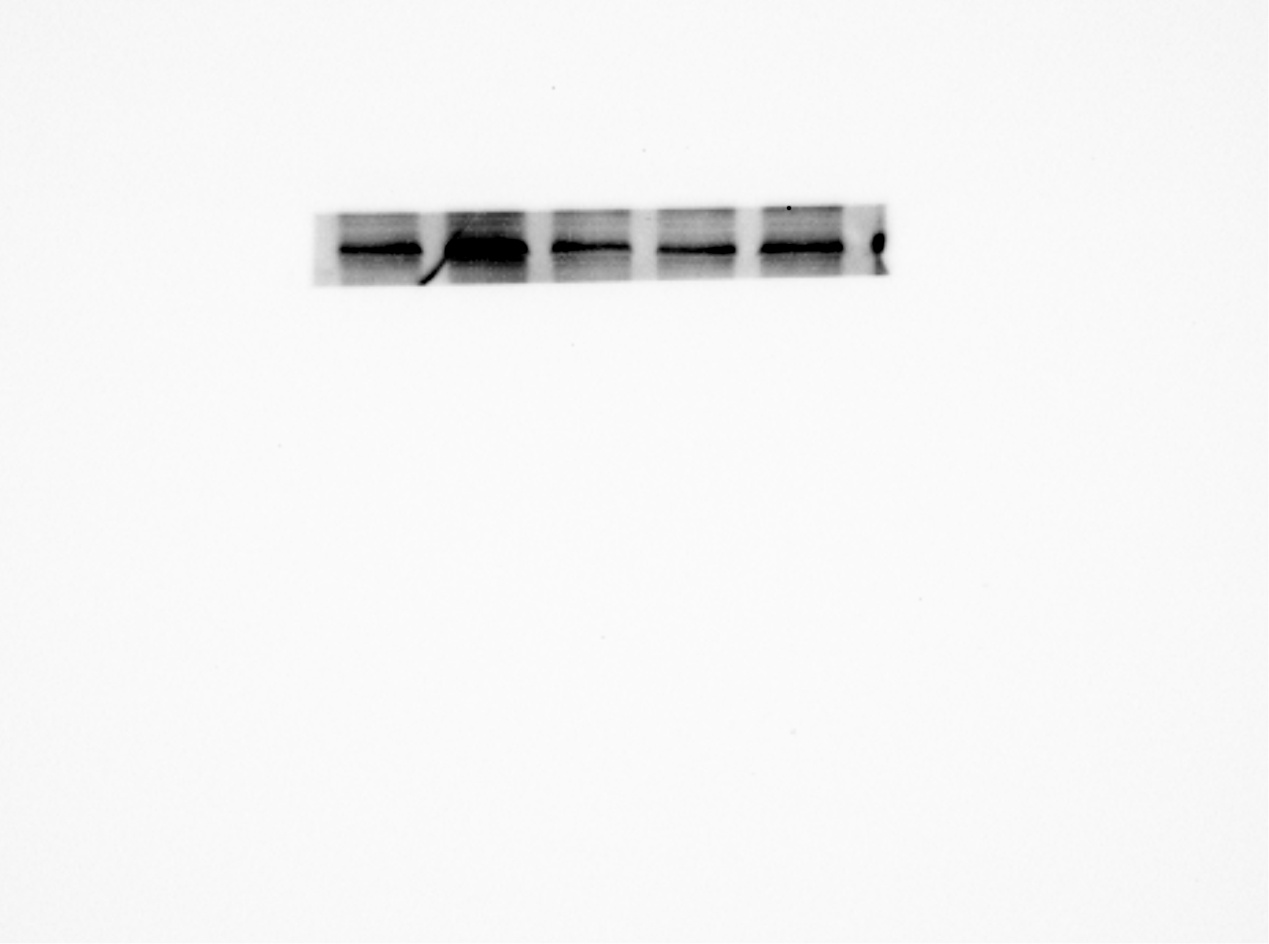


β-Actin


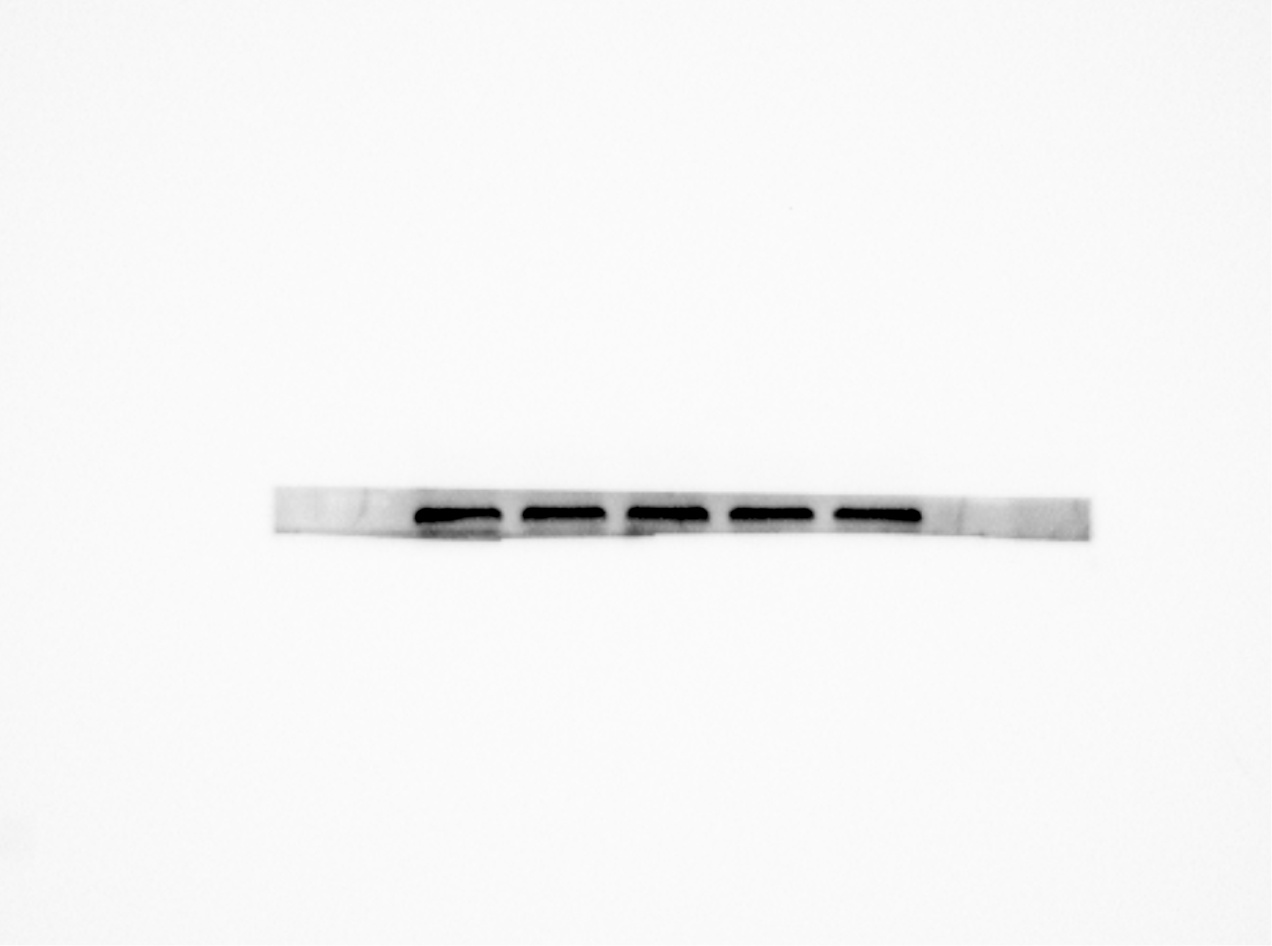


Fig. 8C

ASGR1


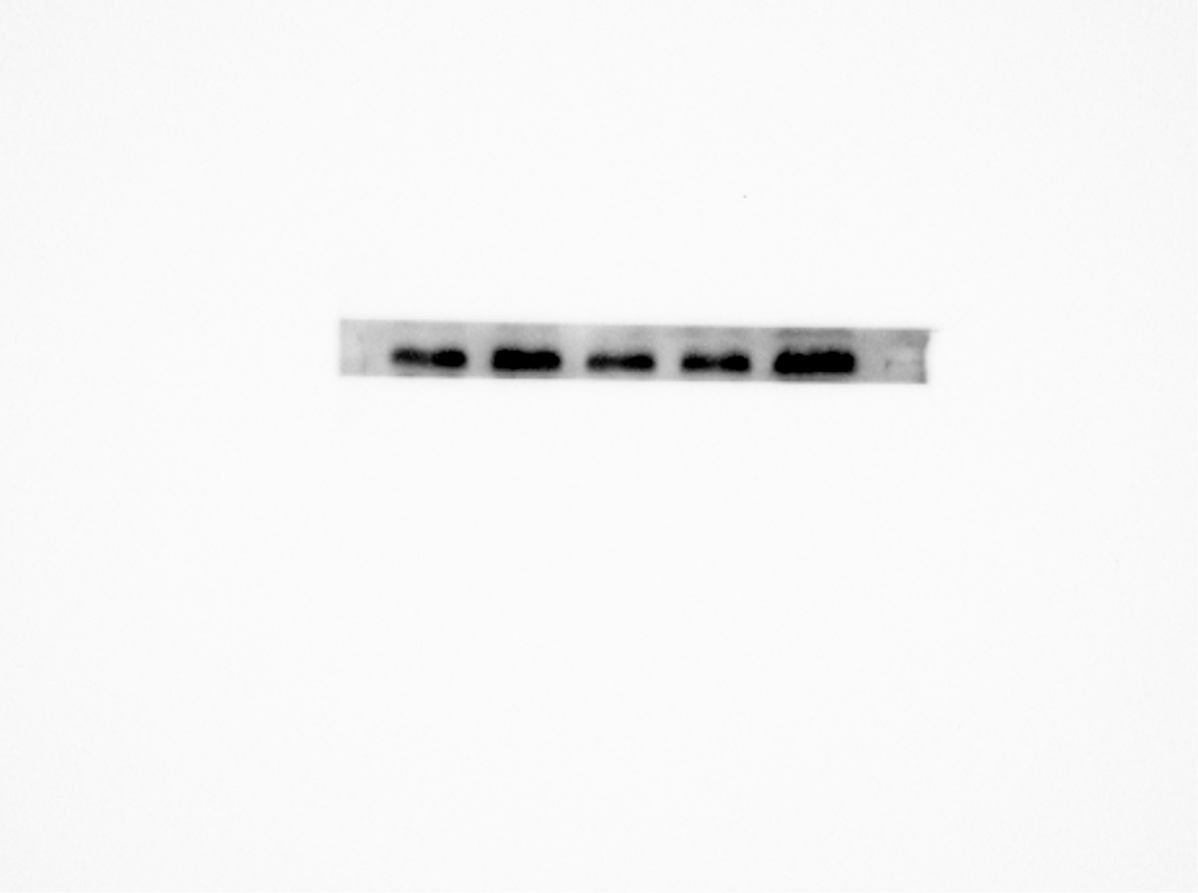


β-Actin


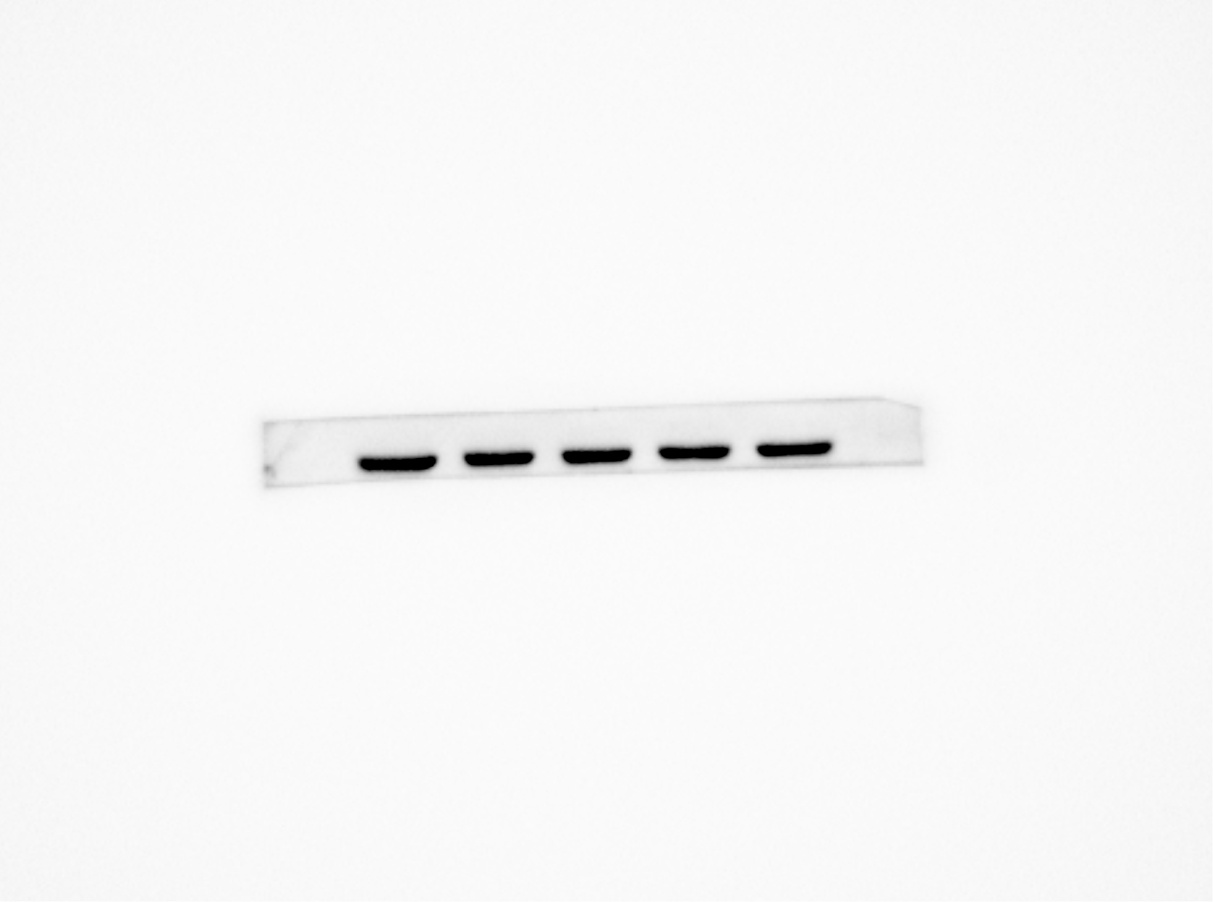


Fig. 8F

p-S6K


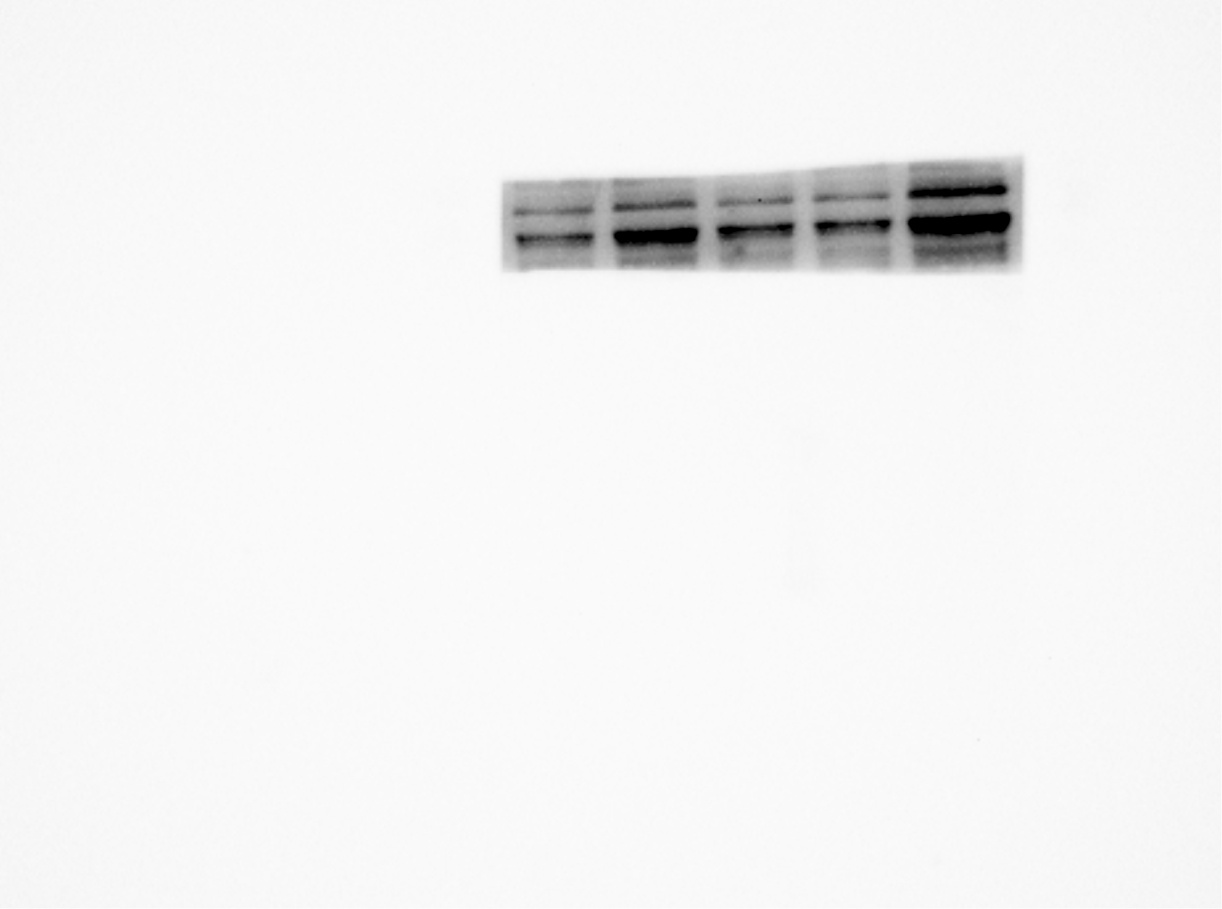


S6K


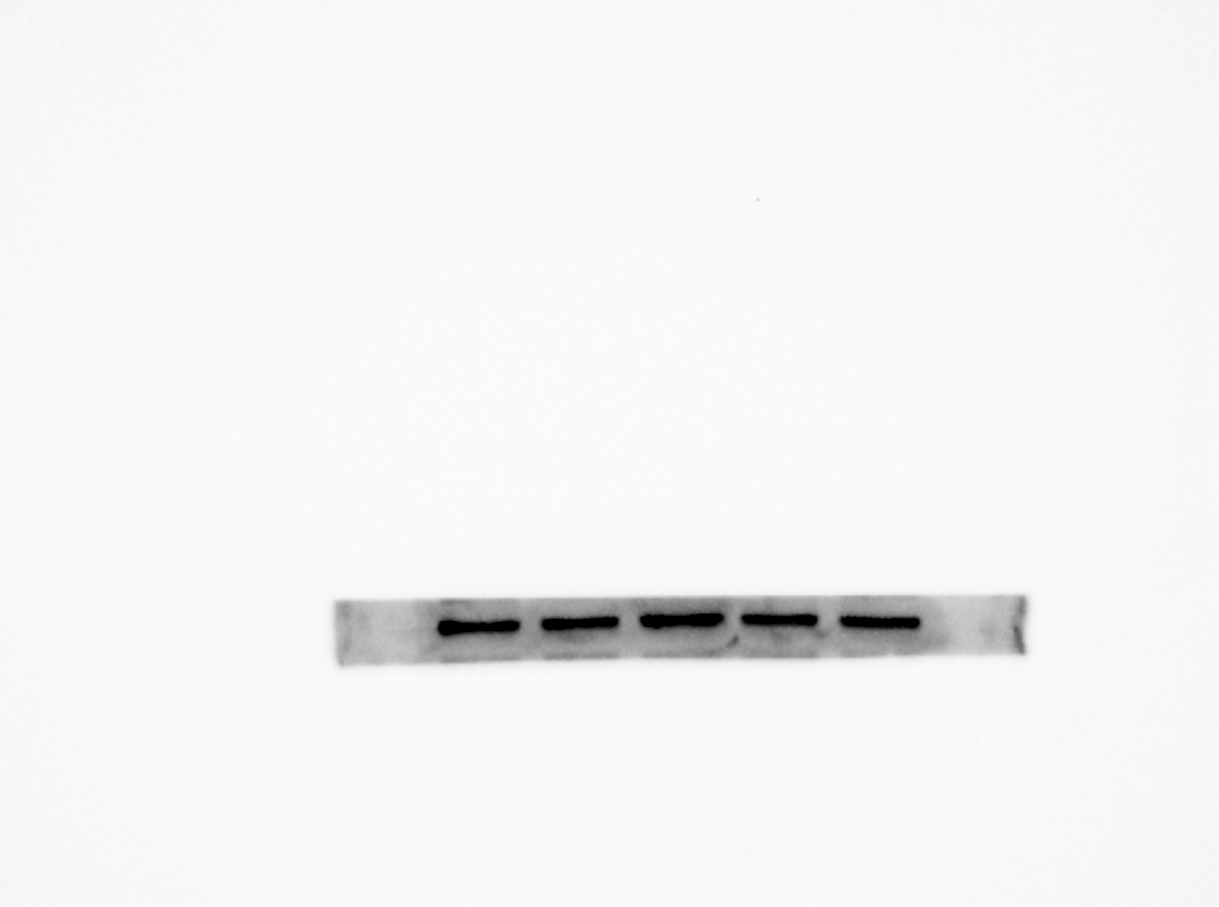


p-mTOR


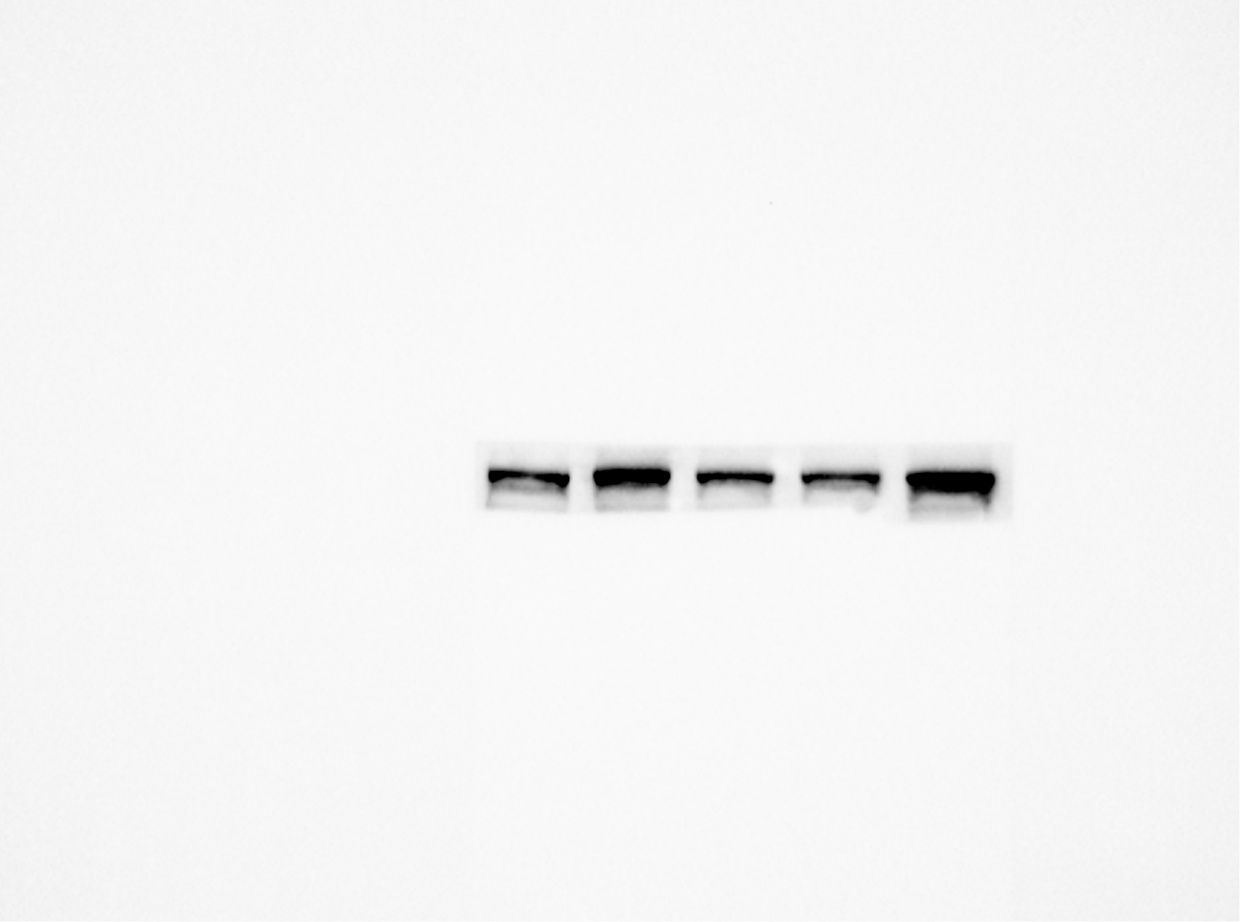


mTOR


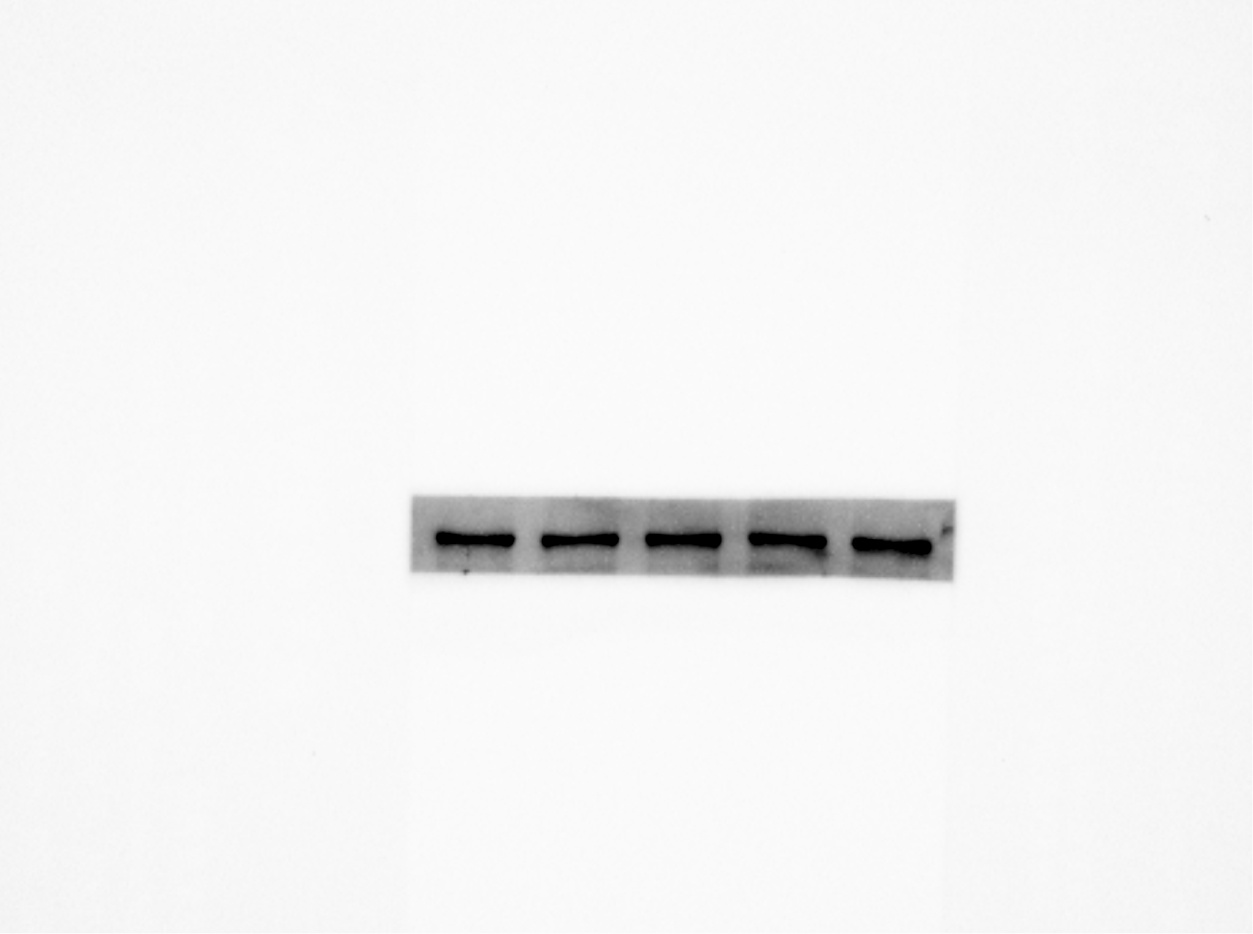


p-AMPK


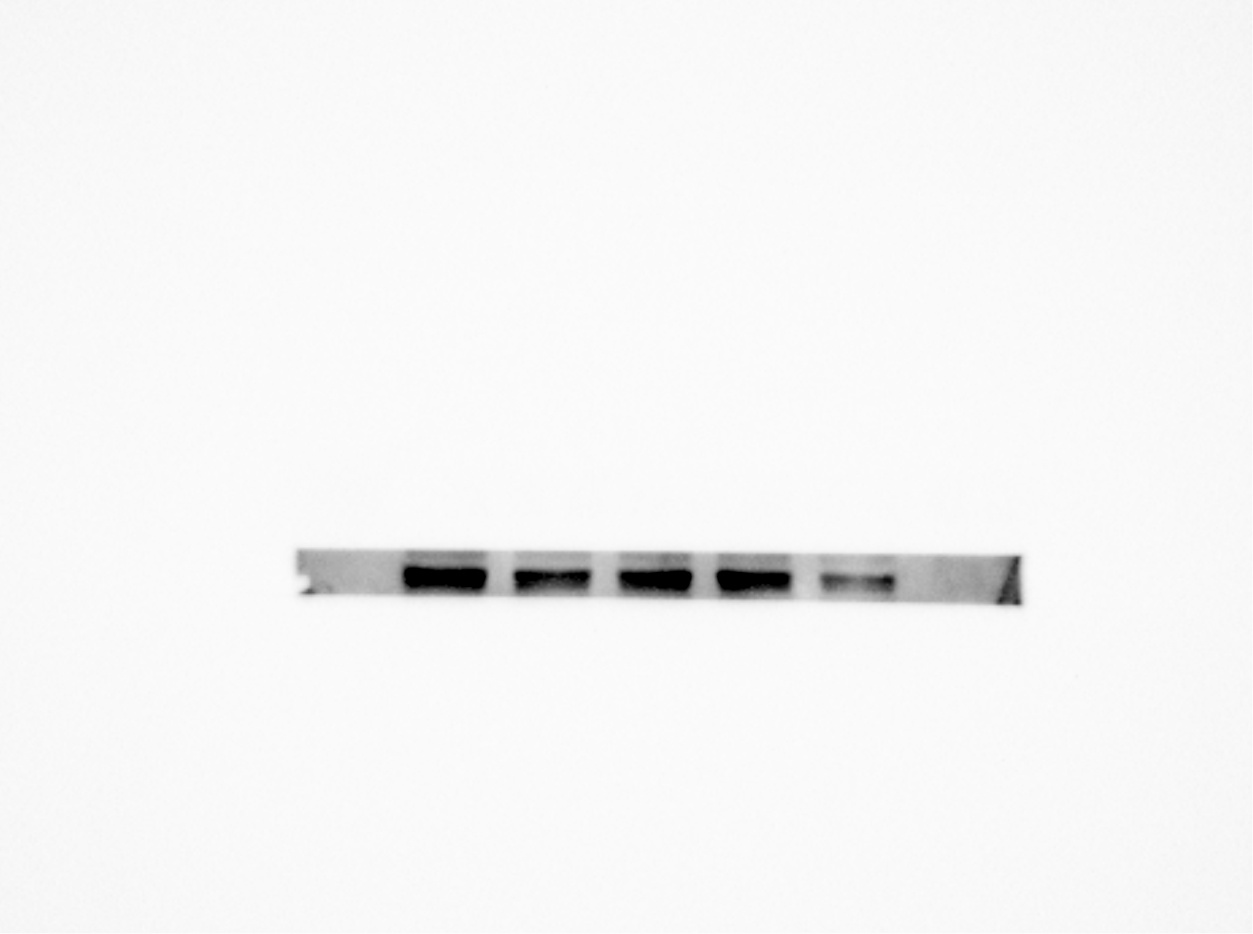


AMPK


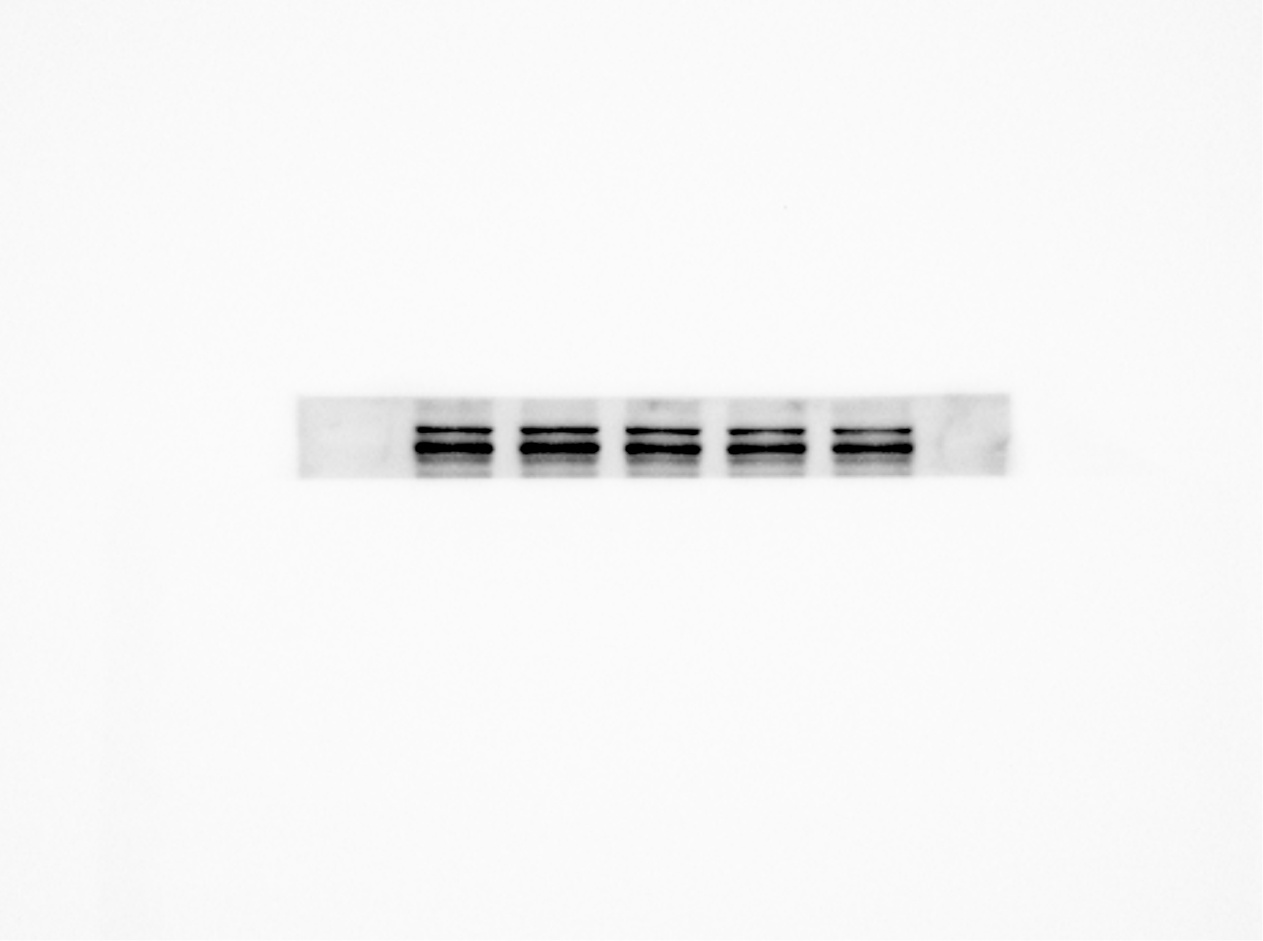


BRCA1


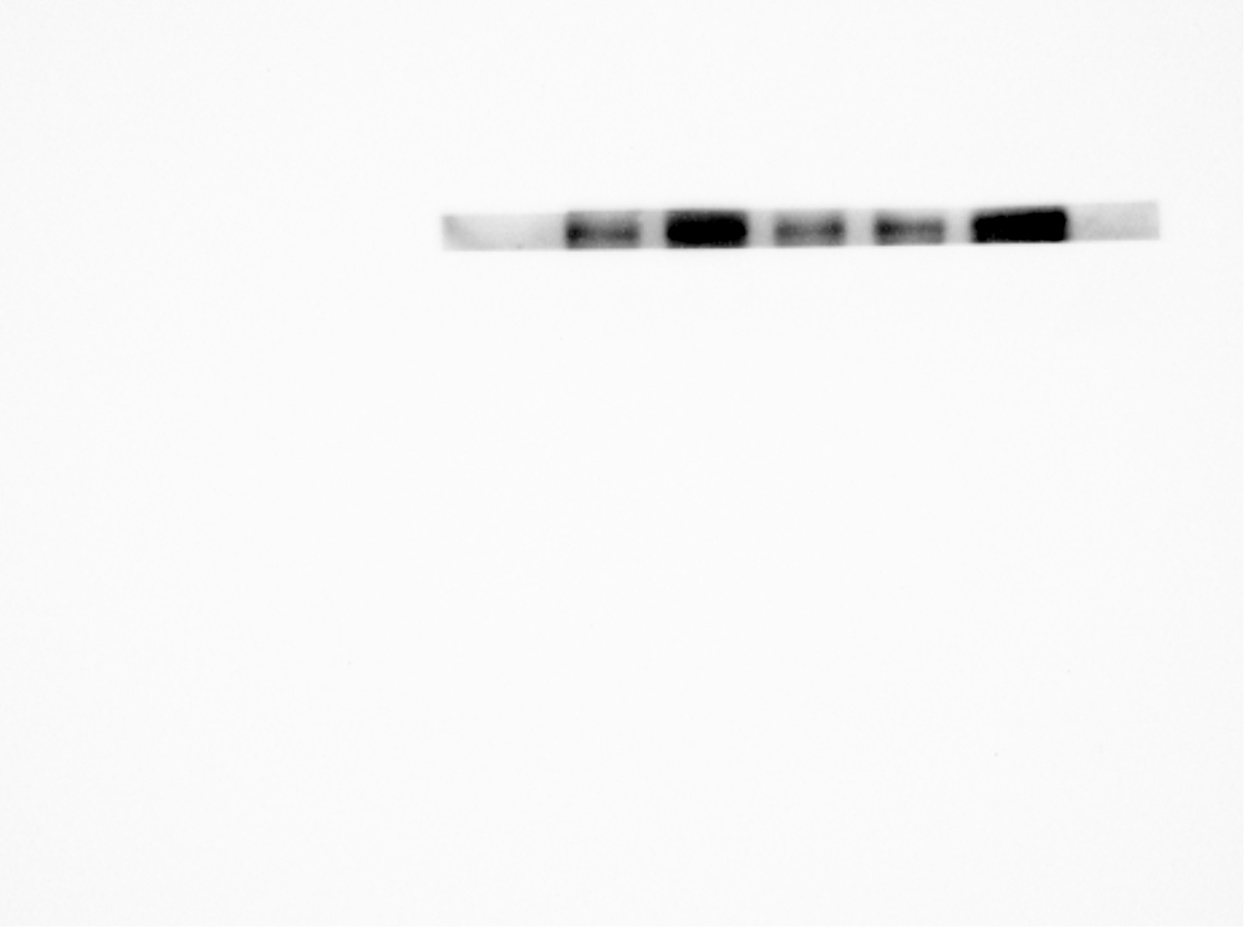


BARD1


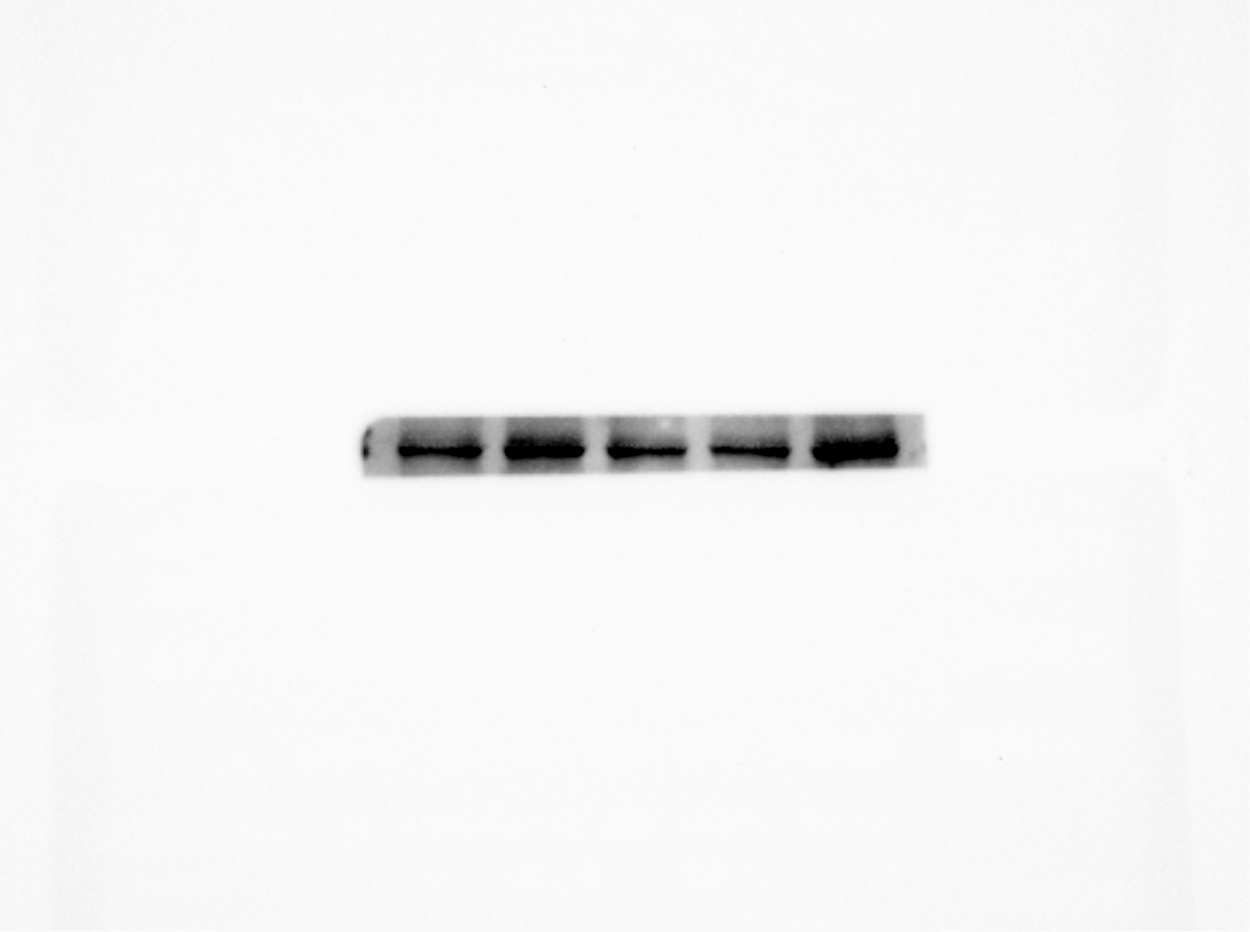


LXRα


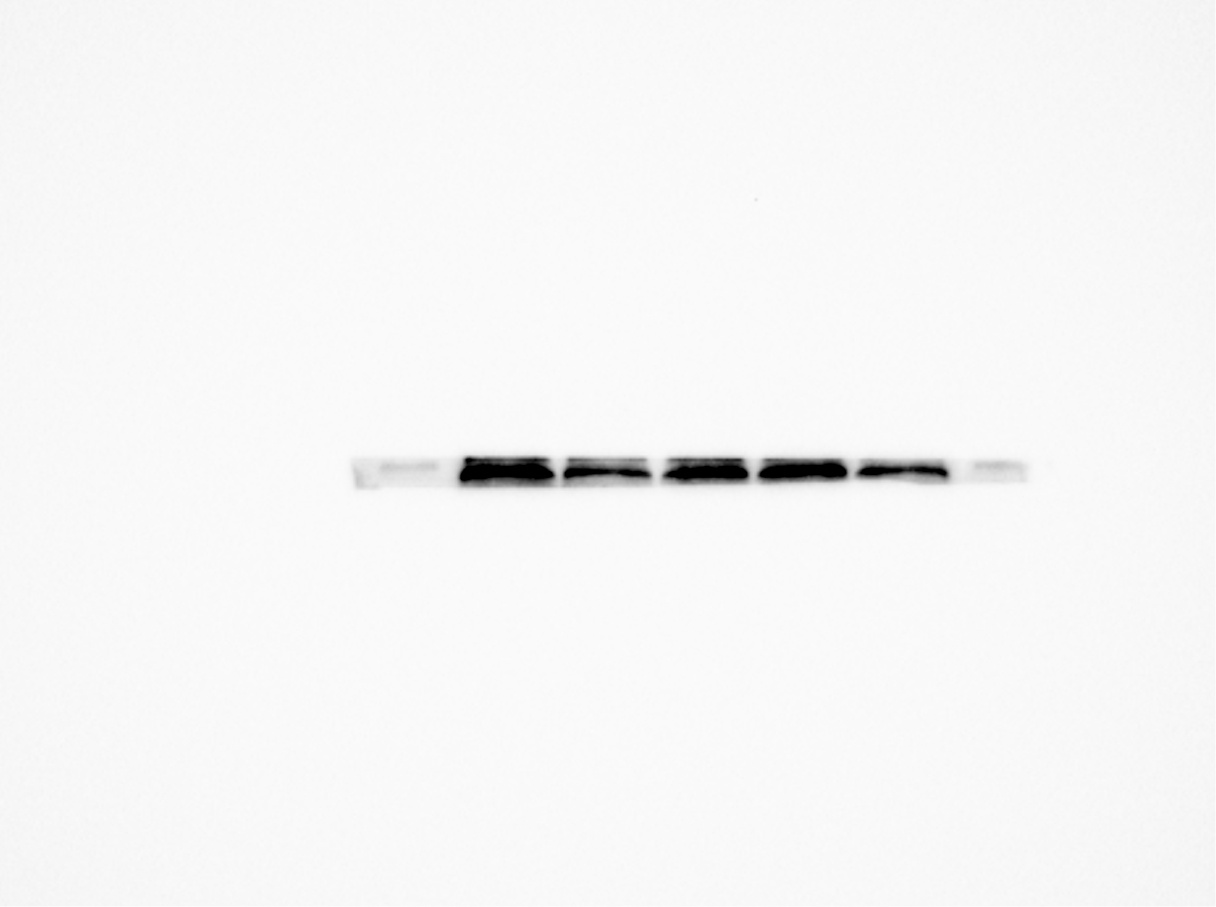


ABCG5


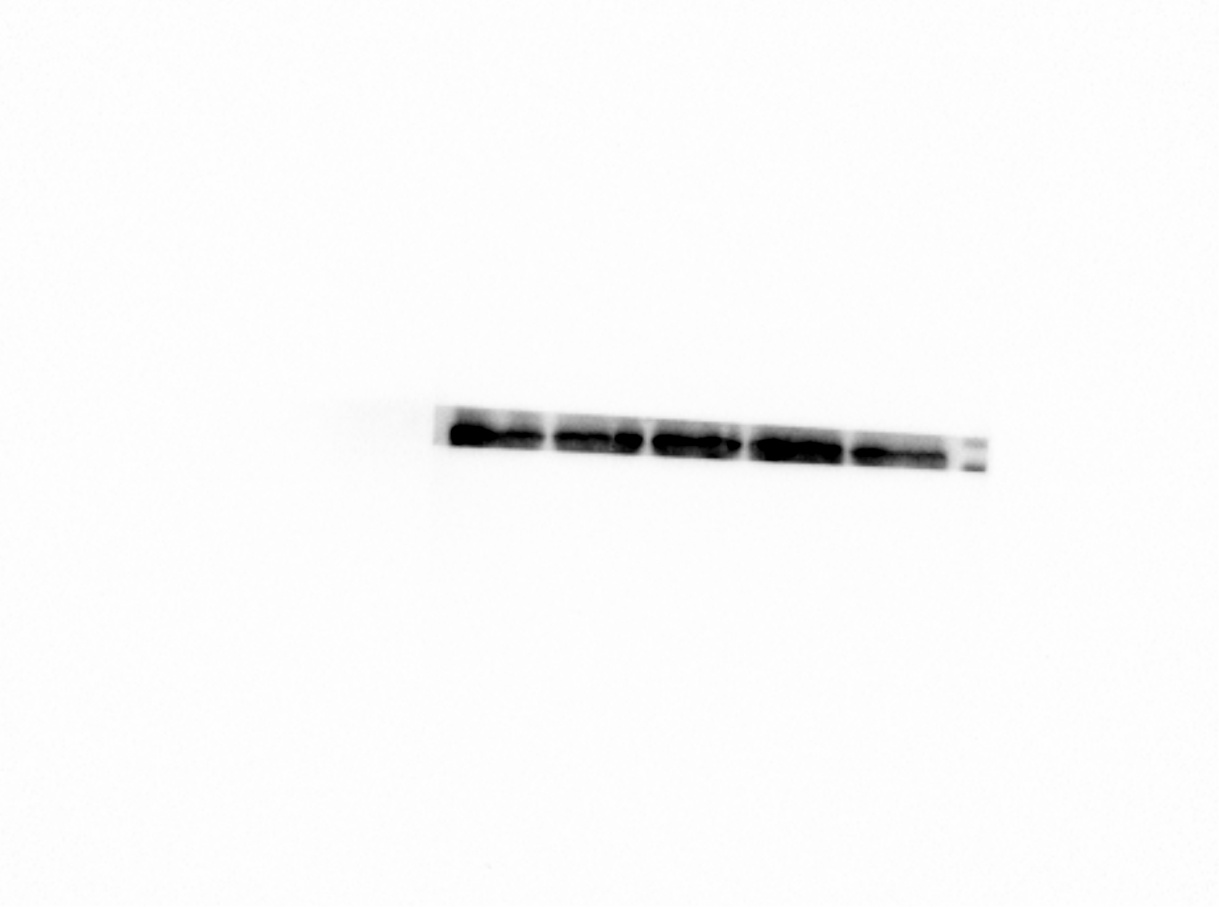


ABCG8


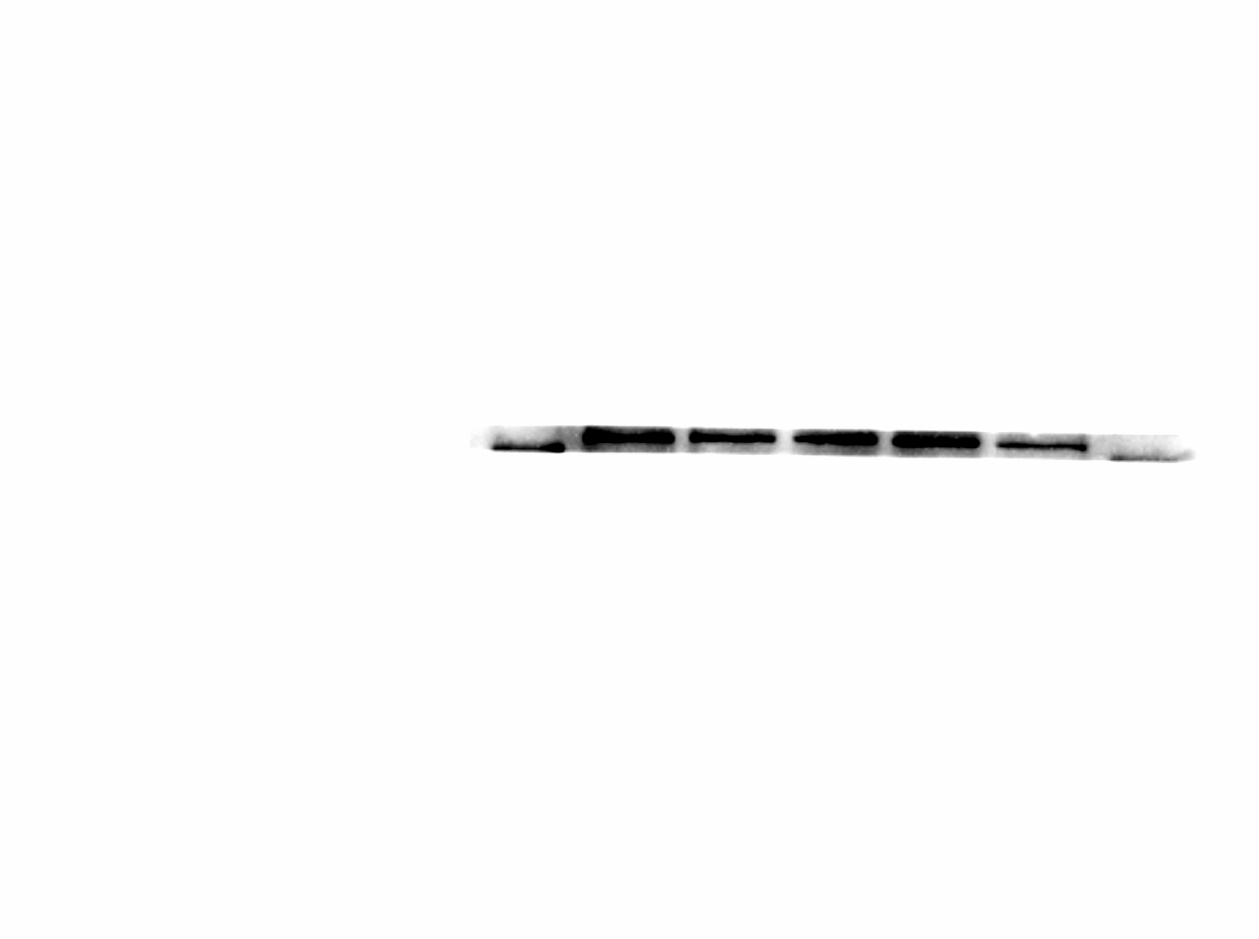


β-Actin


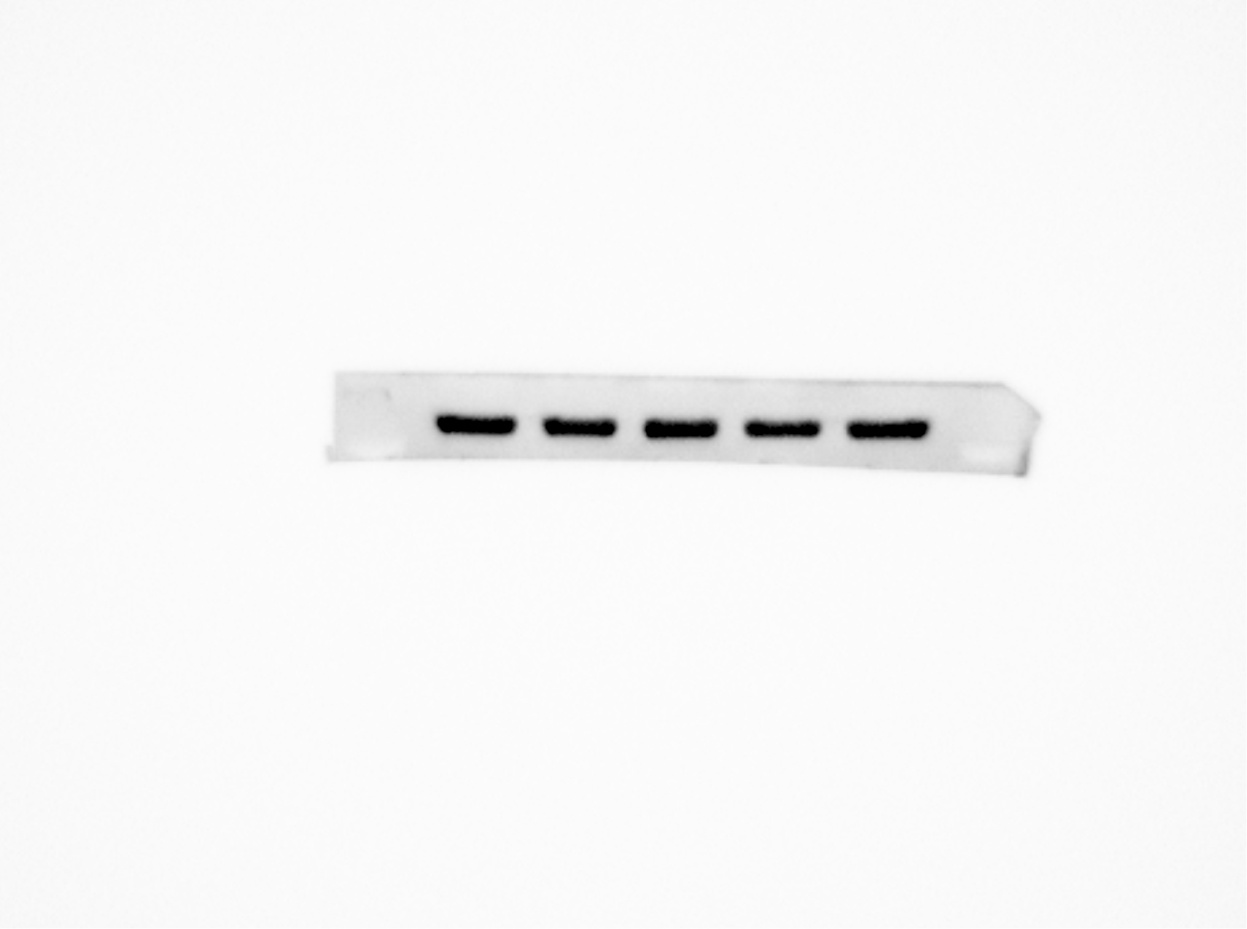

Supplement: Supplementary file 1 — Supplementary Material 1. [file 12951_2026_4181_MOESM1_ESM.docx]
